# Genome-based microbial ecology of anammox granules in a full-scale wastewater treatment system

**DOI:** 10.1038/ncomms11172

**Published:** 2016-03-31

**Authors:** Daan R. Speth, Michiel H. in 't Zandt, Simon Guerrero-Cruz, Bas E. Dutilh, Mike S. M. Jetten

**Affiliations:** 1 Department of Microbiology, Institute for Water and Wetland Research, Radboud University, Heyendaalseweg 135, 6525AJ Nijmegen, The Netherlands; 2 Theoretical Biology and Bioinformatics, Utrecht University, Padualaan 8, 3584CH Utrecht, The Netherlands; 3 Centre for Molecular and Biomolecular Informatics, Radboud University Medical Centre, Geert Grooteplein Zuid 26-28, 6525GA Nijmegen, The Netherlands; 4 Instituto de Biologia, Universidade Federal do Rio de Janeiro, Avenida Carlos Chagas Filho 373, Rio de Janeiro 21941-902, Brazil; 5 Department of Biotechnology, Delft University of Technology, Julianalaan 67, 2628BC Delft, The Netherlands

## Abstract

Partial-nitritation anammox (PNA) is a novel wastewater treatment procedure for energy-efficient ammonium removal. Here we use genome-resolved metagenomics to build a genome-based ecological model of the microbial community in a full-scale PNA reactor. Sludge from the bioreactor examined here is used to seed reactors in wastewater treatment plants around the world; however, the role of most of its microbial community in ammonium removal remains unknown. Our analysis yielded 23 near-complete draft genomes that together represent the majority of the microbial community. We assign these genomes to distinct anaerobic and aerobic microbial communities. In the aerobic community, nitrifying organisms and heterotrophs predominate. In the anaerobic community, widespread potential for partial denitrification suggests a nitrite loop increases treatment efficiency. Of our genomes, 19 have no previously cultivated or sequenced close relatives and six belong to bacterial phyla without any cultivated members, including the most complete *Omnitrophica* (formerly OP3) genome to date.

## Abstract (web summary)

ANaerobic AMMonium OXidation (ANAMMOX) combined with partial nitritation has been adopted for removal of ammonium from wastewater. Here, Speth *et al*. describe the bacterial metagenome of a partial-nitritation/anammox (PNA) reactor, and provide 23 draft genomes, 19 of which were previously uncharacterized/sequenced/cultivated.

## Graphical abstract

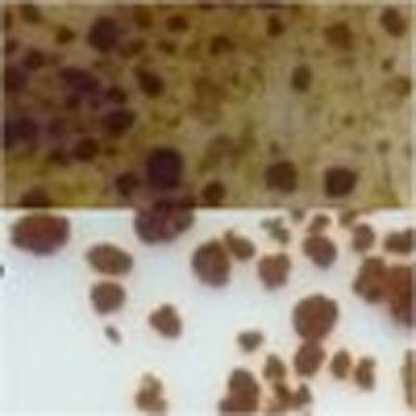

The vast microbial metabolic diversity is a rich source for industrial application and innovation. One large-scale example is wastewater treatment^1^,^2^, where microorganisms remove pollutants, including ammonium, from municipal or industrial wastewater. The paradigm for ammonium removal from wastewater is currently shifting, from removal based on conventional two-step nitrification/denitrification^3^ to a one-step system based on anaerobic ammonium oxidation (anammox)^4^.

In one-step anammox, also known as partial-nitritation/anammox (PNA), aerobic ammonium-oxidizing bacteria (AOB) oxidize part of the influent ammonium to nitrite. Subsequently, anammox bacteria convert the nitrite and remaining ammonium to dinitrogen gas in the absence of oxygen^5^,^6^. The niche differentiation required for these aerobic and anaerobic processes to occur in a single PNA reactor can be achieved in various ways, such as biofilms on a carrier or in granular biomass^7^,^8^. In all single-reactor PNA variants, an outer layer of aerobic organisms consumes the available oxygen, leaving the interior of the biofilm or granule anaerobic for anammox^9^.

PNA has several advantages over conventional ammonium removal via nitrification/denitrification. As no additional electron donors such as methanol are required and nitrous oxide (N_2_O) is not an intermediate of the anammox process, PNA emits less greenhouse gases than conventional systems^10^. In addition, PNA has a lower cost and energy requirement, because the process can take place in a single reactor with limited aeration^2^,^11^. In the decade since the first pilot plant, these benefits have resulted in the implementation of the PNA process for nitrogen removal in over 100 full-scale plants around the world^12^. However, despite the increasing global importance of PNA systems, a comprehensive study of the microbial community facilitating nitrogen removal is lacking.

Previous studies of the PNA microbial community reported on the organisms responsible for the key processes in PNA systems: AOB and anammox bacteria (examples include refs 13, 14). In addition, fluorescence *in situ* hybridization and clone libraries revealed the presence of nitrite-oxidizing bacteria (NOB) in PNA systems and various studies showed that uncultured members of the phyla *Bacteroides*, *Chlorobi* and *Chloroflexi* are omnipresent in anammox bioreactors^13^,^15^,^16^,^17^. Moreover, four recent studies reported 16S rRNA gene amplicon sequencing on two lab-scale and two full-scale PNA reactors, allowing a more detailed insight in the community composition^18^,^19^,^20^,^21^. However, these insights into the microbial community were based on 16S rRNA gene inventories only, either through PCR or fluorescence *in situ* hybridization, and most of the detected organisms were only distantly related to cultured organisms. Thus, both the functional content of their genomes and their role in PNA systems remain unknown. To gain comprehensive insight in the function of the total community in a full-scale PNA reactor, we used a shotgun metagenomics approach followed by a genome-centred metagenome analysis pipeline to retrieve near-complete genome sequences from members of the microbial community. Based on these genome sequences, we present an ecological model of the PNA wastewater treatment system.

Our model system was the Olburgen reactor, a full-scale (600 m^3^) PNA reactor treating wastewater from a potato-processing plant in The Netherlands^22^. This reactor is of particular interest, as its sludge is used to inoculate PNA reactors in other wastewater treatment plants around the world (Paques b.v.). In the Olburgen PNA reactor, the required niche differentiation is achieved using granular sludge without carrier. In addition to the granular sludge, the Olburgen reactor and other granule-based PNA systems also contain less dense, flocculent biomass (Fig. 1). In our experimental design we enriched the anaerobic community in part of the samples by washing the granules before DNA extraction, thereby removing the floccular biomass. This enabled the identification of two distinct communities, the micro-aerobic community in the flocs and on the granular surface, and the anaerobic community in the granule core. Moreover, it enabled us to use differential coverage binning to identify contigs that were derived from the same microbial genome^23^,^24^,^25^. This approach, combined with sequence composition-based methods, enabled us to extract 23 draft genomes representing the majority (59% of the reads) of the microbial community in the Olburgen PNA reactor. Together, the obtained draft genomes provide a comprehensive, system-wide overview of the community in the PNA reactor.

## Results

### Community overview

Sequencing, assembly and binning of the metagenomic sequencing reads (Supplementary Table 1) resulted in 23 high-quality (estimated >80% completeness) draft genomes (Table 1 and Supplementary Fig. 1). These 23 draft genomes accounted for 59% of the original sequencing data from the untreated samples (Supplementary Table 2). This indicates that our sequencing effort provided sufficient resolution to obtain a comprehensive insight in the prokaryotic microbial community of the system.

As expected, three of the most abundant bacteria in the system are relatively well-studied nitrogen cycle organisms: anammox bacterium ‘Candidatus *Brocadia sinica*', aerobic AOB *Nitrosomonas europaea* and NOB *Nitrospira sp*. (Table 1). The remaining 20 genomes represent organisms of the phyla *Bacteroidetes* (five genomes), non-phototrophic members of the *Chlorobi* (four) and *Chloroflexi* (three), *Acidobacteria* (one), *Armatimonadetes*/OP10 (one) and candidate phyla *Omnitrophica*/OP3 (one), *Microgenomates*/OP11 (two), WS6 (two) and *Parcubacteria*/OD1 (one). For convenience, we will hereafter refer to these 23 organisms by their identifiers listed in Table 1.

**Figure 2:**
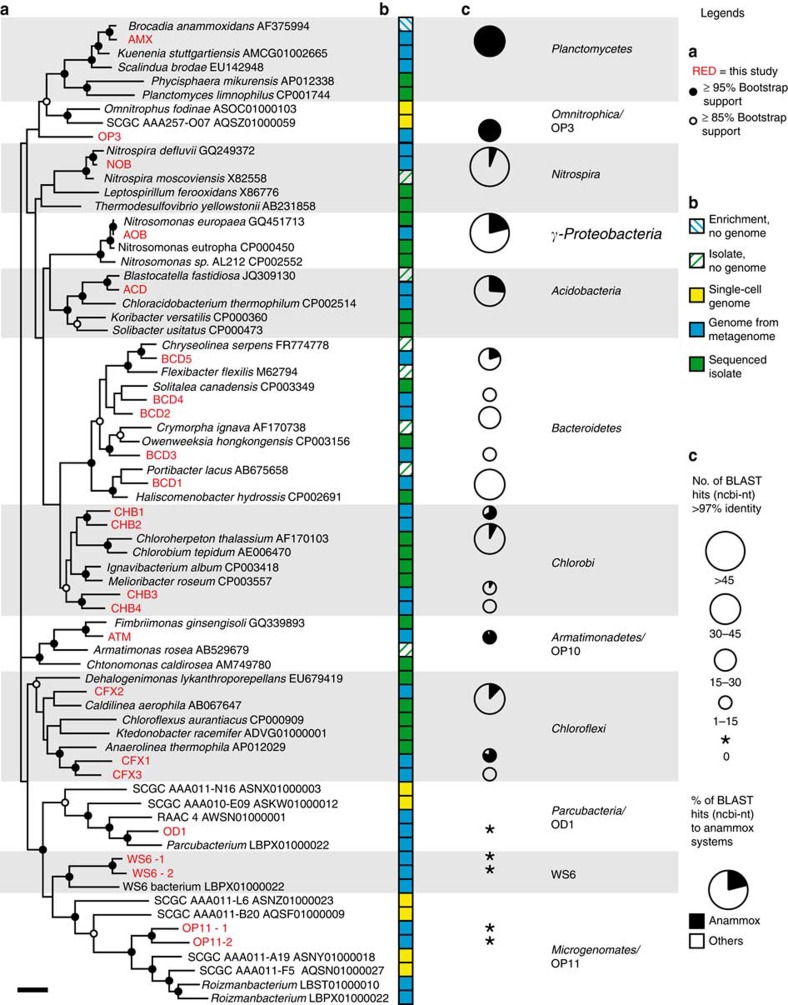
Phylogeny and previous detection of the 23 dominant organisms. (**a**) 16S rRNA gene phylogeny of the obtained genomes and selected related organisms. Organisms in red were obtained in this study. Phylum level lineages (and the class *Gammaproteobacteria*) are indicated by background shading. Scale bar represents 10% sequence divergence. (**b**) Availability of isolate and/or genome for each organism in the tree. (**c**) Previous detection of the organisms obtained in this study using BLAST against the NCBI-nt and their occurrence in other aerobic or anaerobic anammox systems. Data are available in Supplementary Table 3.

Analysis of the 16S rRNA genes from the draft genomes revealed that most of these organisms have been detected previously in other (partial nitritation) anammox systems, indicating that our work has broad relevance for understanding the community and processes in PNA systems (Fig. 2c and Supplementary Table 3). However, with the exception of AMX, AOB and NOB, none of the organisms whose genomes were obtained here have close relatives that are cultured or previously sequenced (16S rRNA gene identity ranging from 81 to 93% and from 81 to 90%, to cultured or sequenced organisms, respectively, see Fig. 2b and Supplementary Table 3), emphasizing the lack of knowledge on the microbial community of PNA systems.

### Identification of distinct aerobic and anaerobic communities

**Figure 3:**
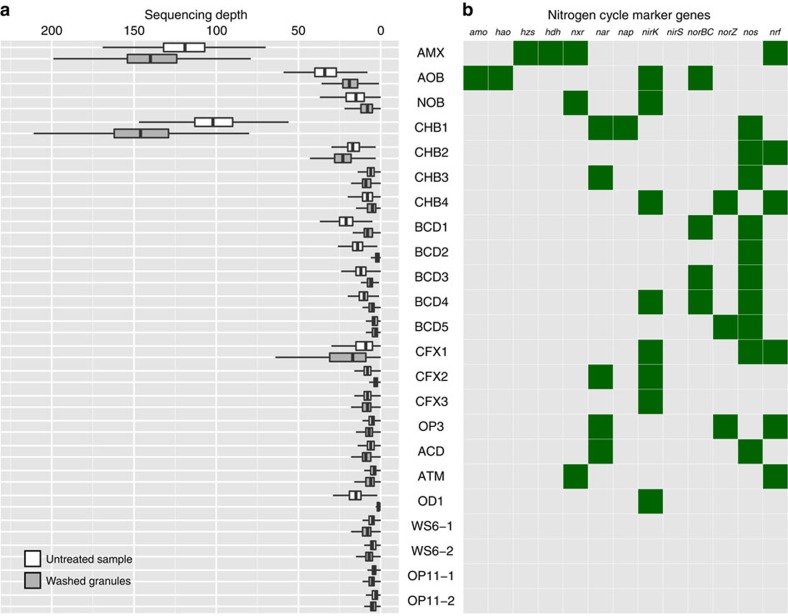
Abundance of the 23 dominant organisms in washed and untreated sample and their potential for nitrogen cycling. (**a**) Box plots of the per-base sequencing depth of each genome, representing the average of the organic extraction data from both sampling points. Light grey indicates untreated sample and dark grey indicates washed granules. Data are available in Supplementary Table 6 (**b**) The presence/absence analysis of key nitrogen cycle genes from the draft genomes. Green indicates the presence and light grey indicates the absence. *amo*, ammonium monooxygenase; *hao*, hydroxylamine oxidoreductase; *hdh*, hydrazine dehydrogenase; *hzs*, hydrazine synthase; *nap*, periplasmic nitrate reductase; *nar*, cytoplasmic nitrate reductase; *nirK*, copper containing nitrite reductase; *nirS*, cytochrome *cd*_1_ nitrite reductase; , *norB*, cytochrome *c*-dependent nitric oxide reductase; *norZ*, quinol-dependent nitric oxide reductase; *nos*, nitrous oxide reductase; *nrf*, pentaheme nitrite reductase; *nxr*, nitrite oxidoreductase. Although a gene cluster with high homology to *Nitrospira*/*Nitrospina nxr* is present in ATM, this organism is unlikely to be a chemolithoautotrophic nitrite oxidizing bacterium.

Our experimental design capitalized on the niche differentiation required for the coupling of aerobic and anaerobic processes in a single reactor, and provided insight into the location of the 23 organisms (Fig. 3a and Supplementary Table 2). The dense granules have a steep oxygen gradient, with a micro-aerobic outer layer surrounding a large anaerobic core^9^. As the flocculent biomass is both smaller and less dense than the granules, it is unlikely that the flocs harbour anaerobic pockets^17^,^26^. Thus, the hypothesis was that the ‘washed granule' fraction would be enriched for anaerobic organisms, whereas the untreated sample would contain a larger fraction of the micro-aerobic organisms in the system. Because of the presence of micro-aerobic niches on the granule surface, all organisms were expected to be present in both fractions. As we expected most organisms to be present in both fractions, and we expected that the granular biomass would be enriched for the community of interest for reactor performance, we decided not to extract and sequence DNA from the flocculent fraction separately.

This niche differentiation hypothesis was supported by the higher abundance of the aerobic organisms AOB and NOB in the untreated sample and the higher abundance of the anaerobic AMX in the washed granules fraction (Fig. 3a). In addition, most of the organisms capable of nitrate respiration (Fig. 3b) and the hydrogenase-encoding organisms (CHB2, CFX1 and OP3) were present in the anaerobic fraction, with the exception of CFX3, which could not be confidently assigned. For the interpretation of our results, we assume that the outer layer of the granules and the flocs face similar micro-aerobic (0–3 mg l^−1^ dissolved O_2_) conditions and, therefore, that the micro-aerobic organisms in the reactor may have a niche both in the flocs and on the granule surface.

### Potential for nitrogen conversions in the PNA system

Most organic carbon and phosphorus is removed from the wastewater in two upflow anaerobic sludge bed reactors and a struvite precipitation reactor, respectively, before the influent enters the Olburgen PNA reactor^22^. The concentrations of ammonium, nitrate and chemical oxygen demand (COD) in the influent and effluent over a 4-month period around the time of sampling are provided and indicate the reactor performance was stable (Table 2). The influent is rich in ammonium (200–320 mg l^−1^ NH_4_^+^-N) and contains residual (recalcitrant) organic carbon (150–225 mg l^−1^ COD) and dissolved carbon dioxide (CO_2_). Moreover, the reactor is aerated continuously, leading to a micro-aerobic environment (0–3 mg l^−1^ dissolved O_2_). Part of the ammonium flowing into the reactor is converted to nitrite, which is detectable at all times (typical range 1–11 mg l^−1^ NO_2_^−^-N). When nitrite builds up to levels higher than 11 mg l^−1^ NO_2_^−^-N, part of the spent air is recirculated, reducing the oxygen concentration and, consequently, the nitrite concentration in the system^22^.

As the PNA reactor is an ammonium-driven ecosystem, we first evaluated the genomes for marker genes encoding key enzymes relevant to the nitrogen cycle (Fig. 3b). The marker genes used were ammonium monooxygenase (*amo*) and hydroxylamine oxidoreductase (*hao*) for ammonium oxidation^27^,^28^, nitrate reductase (*nar* and *nap*) and nitrate oxidoreductase (*nxr*) for interconversion of nitrite and nitrate^29^,^30^, nitrite reductase (*nirK* and *nirS*), nitric oxide reductase (*norBC* and *norZ*) and nitrous oxide reductase (*nos*) for denitrification^30^, pentaheme nitrite reductase (*nrf*) for respiratory ammonification^31^, and hydrazine synthase (*hzs*) and hydrazine dehydrogenase (*hdh*) for anammox metabolism^6^,^32^.

In the aerobic community, nitrification is the predominant nitrogen metabolism. AOB and NOB encoded the key enzymes for ammonia (*amo* and *hao*) and nitrite oxidation (*nxr*), respectively (Fig. 3b). AOB, NOB, BCD4, CFX2 and OD1 encode a copper-containing nitrite reductase (*nirK*), possibly used for detoxification, to cope with fluctuating nitrite levels in the reactor. All *Bacteroidetes* species, BCD1-5, encoded a nitrous oxide reductase. Although nitrous oxide is not an intermediate of the anammox process^6^, it can either be produced chemically^10^ or by other members of the community (Fig. 3b). Whether the nitrous oxide reductases are expressed and functional and thus play a role in mitigation of nitrous oxide emissions from this PNA reactor remains to be investigated.

**Figure 4:**
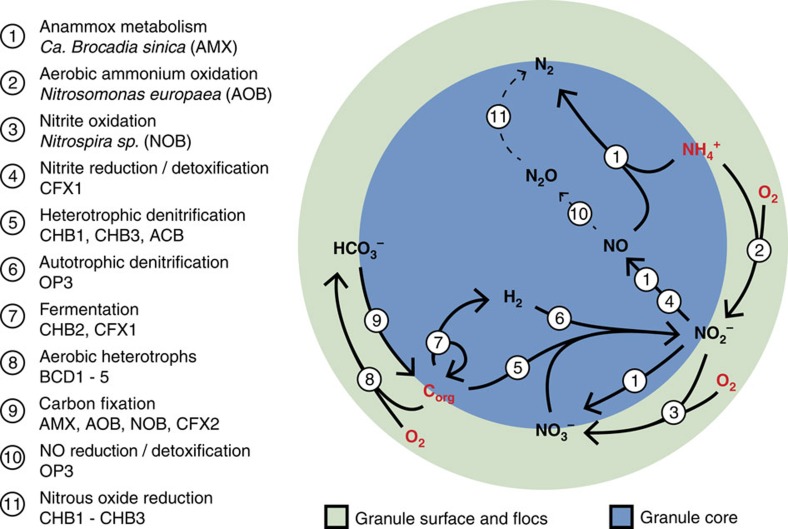
Schematic overview of nitrogen conversions in the Olburgen PNA reactor. Metabolites indicated in red, ammonium (NH_4_^+^), organic carbon (C-org) and molecular oxygen (O_2_) are supplied in the influent and through aeration/stirring, respectively. In the flocs and on the granule surface C-org is oxidized to carbon dioxide (CO_2_), which can be fixed by the autotrophs in the system. In the flocs and on the granule surface, NH_4_^+^ is oxidized to nitrite (NO_2_^−^). The formed NO_2_^−^ is either reduced to nitric oxide (NO) or further oxidized to nitrate (NO_3_^−^). NO_3_^−^ that is formed in the granules can be reduced again in the anaerobic core, either with C-org or molecular hydrogen (H_2_) as electron donor. H_2_ can be formed through fermentation of organic carbon by CHB2, and CFX1. This cyclic feeding will result in additional C-org removal from the system. NO formed from NO_2_^−^ will be combined with NH_4_^+^ and converted to dinitrogen gas (N_2_) by AMX.

In the anaerobic community, the genome of AMX contained the core genes for hydrazine metabolism (*hzs* and *hdh*) and the gene cluster for nitrite oxidation, all essential for the anammox process. The potential for nitrate respiration is more widespread, as CHB1, CHB3, OP3 and ACD encode a nitrate reductase. Interestingly, none of these organisms encode a known nitrite reductase, suggesting they extrude the formed nitrite, potentially allowing cyclic feeding (Fig. 4 and discussed below). In addition to NOB and AMX, the organisms known to oxidize nitrite to nitrate^5^,^33^, ATM encoded a gene cluster closely related to nitrite oxidoreductases of the *Nitrospira*/*Nitrospina*/anammox clade^34^ (Supplementary Fig. 2). It remains to be investigated whether this organism is indeed capable of nitrite oxidation, as the ATM genome lacks genes required for chemolithoautotrophic growth. Interestingly, organisms closely related to ATM have almost exclusively been detected in anammox systems (Fig. 2c and Supplementary Table 3).

None of the genomes retrieved encoded a complete denitrification pathway from nitrate (or nitrite) to dinitrogen gas (Fig. 3b), suggesting that partial denitrification and exchange/transfer of nitrogen cycle intermediates could play an important role in the system. A similar fragmentation of the denitrification pathway was also observed in genome-resolved metagenome studies of aquifer and estuary sediments^35^,^36^, illustrating the added value of this type of genome-resolved analysis over methods only based on gene or pathway presence.

### Aerobic respiration and fermentation and carbon fixation

In addition to the conversion of nitrogen compounds, organisms can play important roles in the system via removal of organic matter, maintaining granule integrity, or by providing growth substrates for other community members. Besides nitrifiers AOB and NOB, most of the aerobic community consisted of aerobic heterotrophs (BCD1-5 and CHB4). Inside the granule core, fermentative organisms (CHB2, CFX1 and CFX3) and denitrifiers (CHB1, CHB3, ACD and OP3) potentially remove organic carbon and nitrate. A known pathway for carbon fixation could not be identified in any of these organisms. Even though PNA systems are thought to be autotrophic^8^, only AOB, NOB, AMX and CFX2 seem to be capable of fixing carbon through one of the described pathways. In addition, the majority of the microbial community was found to be auxotroph for one or more amino acids, as determined by the absence of at least two genes from the biosynthetic pathway. Although the requirements for organic carbon could be satisfied through the influent, it seems likely to be that the amino acid requirements are met through primary production by autotrophs in the reactor.

### Integrating inferred niches and activities in a system model

Based on the functions derived from the assembled draft genome sequences, we propose an ecological model of the nitrogen cycle reactions catalysed by the microbial biomass in the Olburgen PNA reactor (Fig. 4). In the flocculent biomass that is dominated by nitrifiers (AOB and NOB) and *Bacteroidetes sp*. (BCD1-5), the main processes are the aerobic oxidation of organic carbon to CO_2_ and complete nitrification of ammonium to nitrate (Fig. 4). The formed CO_2_ either escapes to the atmosphere or is used by the primary producers (AOB, NOB and CFX2) for growth and biosynthesis (Fig. 4). A small amount of nitrate may be reduced to nitrite coupled to the oxidation of organic matter by CFX2 (not shown in Fig. 4).

Similar to the flocs, the outer layer of the granules is also exposed to oxygen. We expect that similar processes are occurring there, although there will be more competition for the formed nitrite on the granule surface (Fig. 4). Most nitrite is used by AMX to oxidize ammonium but a part is oxidized to nitrate by NOB and AMX. Formed nitrate can be reduced to nitrite, using either organic matter or hydrogen as the electron donor, by CHB1/CHB3/ACD and OP3, respectively, making additional nitrite available for AMX (Fig. 4). The hydrogen required for autotrophic nitrate reduction can be formed through fermentation of organic matter by CHB2 and CFX1. The organic matter required for both nitrate reduction and fermentation can come from the substantial amount of electron donor, measured as COD, in the influent^28^ (Table 2), dead organic matter in the granule or extracellular granule matrix synthesized by autotrophs.

To what extent nitrite respiration contributes to the nitrite removal in the system remains to be investigated. It was clear that many organisms in the Olburgen PNA reactor have the potential to reduce nitrite to nitric oxide (Fig. 3b), but only AOB encodes both nitrite and nitric oxide reductases to further reduce the formed NO to nitrous oxide (N_2_O). This indicates that detoxification, rather than respiration, of nitrite could be the main purpose of the encoded nitrite reductases, releasing the formed NO into solution where it will be removed by the aeration. Alternatively, AMX might metabolize the NO to N_2_ (ref. 37). In line with the latter, a previous study showed that NO and N_2_O emission in the Olburgen PNA reactor dropped when oxygen became limiting^38^. However, it should be noted that the effect of dissolved oxygen on nitrous oxide emissions is not well understood.

Our proposed ecological model offers an explanation for the effective performance of this PNA reactor and is consistent with the performance data of the reactor (Table 2). It also provides an ecosystem role for previously understudied organisms in this bioreactor. However, it should be noted that our model is based on genome content and differential abundance of the genomes across the biomass fractions, and awaits further validation using, for example, metatranscriptomics and metaproteomics, or enrichment/isolation of key organisms from this reactor. We note that one of the critical functions in this reactor, ammonium oxidation to nitrite, is carried out by an organism represented by only 2.9% of the reads, illustrating the need for activity-based follow-up work. Previous studies, such as the work by Ishii *et al.*^39^ on extracellular electron transfer in a lab-scale bioreactor or by Stewart *et al.*^40^ in the Eastern Tropical South Pacific oxygen minimum zone, have shown that the correlation between organism abundance and activity can be weak. The study by Ishii *et al.*^39^ further illustrates the power of metatranscriptomics in discovery of novel gene products with important roles in community functioning. We expect that our results will provide a valuable groundwork for such future studies.

### Nitrite loop enhances PNA performance

The presence of NOB in PNA systems is often viewed as detrimental to optimal N-removal, as these organisms compete with both types of ammonium oxidizers for their electron acceptors and produce nitrate, lowering the overall nitrogen removal efficiency. In the Olburgen system, NOB were nearly as abundant as AOB (2.3 and 2.9% of the reads respectively), without noticeable negative effect on reactor performance. Nitrate accumulates in the effluent (Table 2), but this is the amount expected based on the stoichiometry of the anammox process, which produces 0.3 mole of nitrate per mole of nitrite reduced^5^. Thus, excess nitrate accumulation in the system might be mitigated by the presence of (partial) denitrifiers that catalyse the conversion of nitrate back to nitrite. Such cyclic feeding was proposed previously to explain a high NOB:AOB ratio in lab-scale, aerobic sequencing batch reactors with granular sludge, where it was termed the ‘nitrite loop'^41^. *Chlorobi* species CHB1 is probably the biggest contributor to this process. It is the second most abundant organism in the system and preferentially present in the granules (Fig. 3a). An organism with an identical 16S rRNA gene sequence was previously detected in other anammox systems by clone libraries or amplicon sequencing, where it was mislabelled as a *Planctomycete*^16^,^20^. Interestingly, this organism was identified as the most dominant organism in a recent amplicon study of a different full-scale PNA reactor^20^.

A closer look at the metabolic capabilities of CHB1 indicated that it is probably a heterotrophic denitrifier encoding an anion exporter of the *tauE* family at the same genomic locus as the nitrate reductase. Although the only characterized member of the extensive *tauE* family is a sulfite exporter, we predict a role in nitrite extrusion based on its genomic co-localization with the nitrate reductase. Consistent with this prediction, homologues of this protein are also present in CHB3 OP3 and ACD, which also encode a nitrate reductase but lack a nitrite reductase. As many other microorganisms could perform partial denitrification to recycle nitrate to nitrite, this alone is unlikely to account for the abundance of CHB1 in this PNA reactor and similar systems. The organism could be maintaining granule integrity, as was previously suggested for another full-scale PNA reactor^20^ or possibly supply AMX with an essential nutrient.

### Candidate phyla OP11 and OD1 and WS6

In addition to the organisms described above, our work yielded high-quality draft genomes of organisms from the candidate phyla OD1, OP11 and WS6 (refs 42, 43, 44). These phyla belong to the ‘candidate phyla radiation' (CPR) proposed by Brown *et al.*^45^, who recently reported on nearly 800 draft genomes of CPR organisms. The CPR genomes reported here have 16S rRNA genes 78–84% identical to the closest relative described by Brown *et al.*^45^, which further illustrates the large diversity of the CPR (Fig. 2a and Supplementary Table 3). As observed previously for CPR organisms^45^,^46^,^47^, the genomes are very small (0.9–1.4 Mb, Table 1) and their known metabolic and biosynthetic capabilities are limited.

For the OP11 and WS6 organisms, their predicted location in the anaerobic granule core supports a fermentative lifestyle (Fig. 3a), as was suggested for related organisms^23^,^24^,^46^. In contrast, the OD1 organism was strongly depleted in the washed granules. A possible explanation for this could be a parasitic or symbiotic relationship with BCD2, which was equally abundant in the system and similarly depleted in the granules (Fig. 3a). In recent times, an organism from the related TM7 lineage was co-cultivated as (parasitic) epibiont of a strain of *Actinomyces odontolyticus*^48^, supporting this hypothesis. Further work to assess a potential relation between OD1 and BCD2 is required.

## Discussion

Using a metagenomic assembly approach, we retrieved 23 draft genomes from a full-scale PNA reactor, accounting for the majority of the sequenced DNA. Our work on the PNA reactor presents the first system-wide metagenomic characterization of a full-scale engineered system. At the lab-scale, an excellent previous study reported the system-wide characterization of a terephthalate degrading culture, using a combination of single-cell genomics and shotgun metagenomics^49^.

In addition to the obtained genomes, we could assign most of the organisms to niches in the reactor based on our experimental design and genome content. This allowed us to infer an ecological model of the microbial ecosystem, segregated into micro-aerobic and anaerobic compartments (Fig. 4). Our results provide a potential role for the dominant members of the microbial community, which we have integrated in a genome-based model of the Olburgen PNA reactor ecosystem.

In our model, NOBs are not detrimental to the system. Rather than causing build-up of excess nitrate, the nitrate they form can serve as electron acceptor for the degradation of organic matter and the oxidation of fermentation products, including hydrogen, in the granule core. The nitrate formed by NOB and AMX could thus partly be recycled and made available as nitrite for anammox bacteria, further improving effluent quality.

The flocculent biomass contains a large fraction of the nitrifying bacteria in the reactor. The physical separation of nitrifiers in the flocs and AMX in the granule core probably prohibits effective coupling of both processes. Therefore, a stronger selection towards granular biomass, where nitrifiers were present in the outer layer, might improve overall system performance and reduce nitrate in the effluent.

Finally, the presence of organisms closely related to the ones we have described here in other (partial nitritation) anammox systems suggests that our study is of broad relevance for the understanding of PNA systems. Our results contribute to the understanding of PNA systems and highlight the need for further work on the microbial ecology of these innovative, sustainable and increasingly important wastewater treatment systems.

## Methods

### Sampling

The described system is a full-scale (600 m^3^) PNA reactor at the end of the main line of a wastewater treatment plant treating potato-processing wastewater at 35 °C (Olburgen, The Netherlands)^22^. Samples (10 l), consisting of granular and flocculent biomass, were taken from two points in the reactor to assure stirring was sufficient for homogenous biomass distribution, at 1.4 and 3.8 m from the base, on 6 November 2013 (week 45). From both the 10 l samples two representative 50 ml subsamples were taken. The granules of one subsample were washed three times with 1 × PBS, removing the flocculent biomass. Washing was done by pouring off the liquid from 50 ml reactor sample, adding 1 × PBS to a final volume of 50 ml, vigorously shaking and allowing the granular biomass to settle for 3 s before pouring off the liquid and the suspended flocculent biomass. The other subsample was processed without wash steps. All four resulting fractions were homogenized using a potter homogenizer and 1 ml of the homogenized sample was used for each DNA extraction.

### DNA extraction

DNA extraction from each of the four fractions was performed using two different methods, resulting in eight different samples. Two different DNA extraction methods were used to aid in genome binning, based on the method previously published by Albertsen *et al.*^24^. In addition, the use of multiple DNA extraction methods might mitigate the effect of extraction bias of each method. DNA extraction using the PowerSoil kit (MoBio Laboratories Inc., Carlsbad, USA) was performed according to the manufacturer's instructions. Organic solvent extraction using CTAB/Phenol/Chloroform was done using a protocol modified from Zhou *et al.*^50^. Briefly, the samples were pelleted and resuspended in 675 μl CTAB extraction buffer (10 g l^−1^ CTAB, 100 mM Tris, 100 mM EDTA, 100 mM sodium phosphate, 1.5 M NaCl pH 8), 50 μl lysozyme (10 mg ml^−1^) and 30 μl RNAse A (10 mg ml^−1^), and incubated at 37 °C for 30 min. Subsequently, 50 μl proteinase K (10 mg ml^−1^) was added and the samples were incubated at 37 °C for 30 min. Next, 150 μl of 10% SDS was added and the samples were incubated at 65 °C for 2 h. After cell lysis, DNA was recovered using phenol/chloroform extraction and isopropanol precipitation, and resuspended in diethylpyrocarbonate-treated nuclease-free water.

### Metagenome sequencing

All kits described in this paragraph were obtained from Life Technologies (Carlsbad, CA, USA). The library preparation procedure described below was performed separately for each of the eight samples obtained after DNA extraction. DNA was sheared for 5 min using the IonXpress Plus Fragment Library Kit, following the manufacturer's instructions, and barcoded using the IonXpress barcode adapters. Further library preparation was performed using the Ion Plus Fragment Library Kit following manufacturer's instructions, with size selection using an E-gel 2% agarose gel. Emulsion PCR was done using the Ion PGM Template OT2 400 kit and sequencing was performed on an IonTorrent PGM using the Ion PGM 400 bp sequencing kit and an Ion 318v2 chip. For each run, two barcoded libraries were pooled and a total of six runs were performed. The libraries that were pooled had been treated the same way (for example, washed granules where DNA was extracted using the PowerSoil kit), but originated from the two different sampling points. The resulting 30 million reads were imported into the CLC Genomics Workbench (v6.0.4, CLCbio, Arhus, Denmark) and read ends were trimmed using the integrated trimming algorithm. In addition, after end trimming all reads below 100 bp were discarded, resulting in 8.2 Gbp of sequencing data in single reads (Supplementary Table 1).

### Metagenome assembly

All read data were cross-assembled, that is, the data sets were combined and the 27.2 million reads were assembled *de novo* together, to obtain the maximum amount of biologically relevant information on contigs with an average coverage above 15 × . Assembly was performed using the *de novo* assembler incorporated in the CLC Genomics Server software (v6.0.4, CLCbio), using a word size of 35 and a bubble size of 5,000. This assembly resulted in 93,035 contigs larger than 1,000 bp, totalling 272 million bp. Reads (21.2 million reads; 78% of the data, 6.4 Gbp) were incorporated in the assembled contigs. A total of 8,396 of these contigs were longer than 5,000 bp.

### Binning

To bin the metagenomic contigs into draft genomes, we used a multi-step approach employing a mix of available methods and custom scripts. Custom scripts are available at www.github.com/dspeth/bioinfo_scripts.

First, tetranucleotide frequencies of contigs longer than 5,000 bp were used to generate an emergent self-organizing map (ESOM), on which all contigs were subsequently projected^51^,^52^. Using this map, 32,630 contigs were classified into 27 clusters (Supplementary Fig. 3). The contigs in these clusters were extracted using ESOM_bin_parser.pl. Every clustered contig was assigned a cluster number, which was used to visualize the clustered contigs during the manual binning procedure described below.

In addition, coverage, length and GC content of all contigs was obtained using the script fasta_to_gc_cov_length_tab.pl and tetramer content was calculated using calc.kmerfreq.pl (www.github.com/MadsAlbertsen/miscperlscripts)^24^. For all contigs the average coverage in each subsample was determined using the read mapper of the CLC genomics workbench (v7.0.4, CLCbio), with mismatch penalty 2, insertion/deletion penalty 3 and an 80% identity over 50% of the read requirement.

All contig information (full sequence included) was loaded in R and ratios for both differential coverage between DNA isolations and sample treatment were calculated. All data used to extract the 23 genome bins are available on figshare (https://figshare.com/articles/Olburgen_PNA_genome_binning_source_data/1612256). Binning was done manually, based on visualization of the data using the ggplot2 package. Clustering information from ESOM was used as visual aid in bin identification. Bins were extracted and refined based on differential coverage, differential coverage ratios, overall coverage, GC content and tetramer composition. The importance of each metric differed per bin as, for example, AMX could be binned primarily using coverage, whereas, for example, CFX1 sequence composition provided a stronger signal. An overview of the binning procedure used is available at https://github.com/dspeth/bioinfo_scripts/tree/master/binning. All commands run to extract the 23 genome bins are available as Supplementary Note 1. Examples of plots generated during the binning procedure are shown in Supplementary Figs 4–6.

After binning, reads were mapped on the binned contigs using bowtie2 (ref. 53) and the data of each bin were reassembled with SPAdes, using the original CLC contigs as ‘trusted contig'^54^.

### Bin completeness check and refining and annotation

The taxonomic affiliation and completeness of the obtained genome bins were assessed using CheckM^55^. After the completeness estimate, several bins were manually refined by addition of previously unbinned contigs and CheckM was run on the data again. Reported completeness estimates (Table 1 and Supplementary Fig. 2) were based on the 111 essential single-copy genes proposed by Dupont *et al.*^56^. Hidden Markov models (HMMs) of the proteins encoded by these genes (combined in essential.hmm) were downloaded from github.com/MadsAlbertsen/mmgenome/tree/master/scripts. CheckM was used to assess their presence and number of copies in each bin, followed by a manual check for the five bins belonging to candidate phyla OD1, WS6 and OP11.

We first validated that all genomes used the standard genetic code using the FACIL webserver^57^. Next, genome annotation was done using Prokka (v1.10), with the –c flag removed from the prodigal command (line 649) to include partial open reading frames at contig ends^58^,^59^,^60^,^61^,^62^. A set of trusted nitrogen cycle protein sequences (Supplementary Table 4) and custom whole-genome databases based on the taxonomic affiliation of the draft genomes (Supplementary Table 5) were made for annotation with Prokka.

After initial annotation the draft genomes were corrected for persistent frameshifts, common in IonTorrent data^63^, using iontorrent_indel_correcter.pl (https://github.com/dspeth/bioinfo_scripts/tree/master/iontorrent_errors) followed by a round of manual curation. After error correction, the genomes were reannotated using Prokka.

Raw data and assembled, annotated draft genomes are available in GenBank under BioProject accession number PRJNA274364 and the accession numbers listed below and in Table 1.

### Data interpretation

The gene complement of all 23 genomes was analysed for nitrogen cycle genes using text searches of the annotation and sequence searches with BLAST against the contig sequences, to detect misannotated genes or genes missed in annotation^64^. To assess general metabolic capabilities and biosynthetic pathways, annotation searches were combined with analysis in Artemis^65^, BLAST searches and mapping of the protein complement of the draft genomes on Kyoto Encyclopedia of Genes and Genomes pathway maps using KAAS^66^.

Genomic data of each organism were interpreted in view of its location in the biomass, inferred from enrichment or depletion of the sequences from the granule fraction. To confidently assign each draft genome to either the flocs/granule surface niche (micro-aerobic) or the granule core niche (anaerobic), the per-base sequencing depth was calculated using SAMtools^67^ on a BAM file of reads mapping on the assembled contigs and exported from the CLCgenomics Workbench (version 8.5.1). To account for the inherently lower number of reads from the untreated sample mapping to the assembled contigs (Supplementary Table 2), we subsampled the reads obtained from the washed granule fraction to 80% of their total. After this correction, an equal number of reads mapped to the combined assembled contigs (Supplementary Table 6). The distributions of sequencing depth per base were compared using the *t*-test and the Mann–Whitney *U*-test, and visualized as boxplots using R.

### 16S rRNA gene analysis

Phylogenetic affiliation of the bins was initially determined using CheckM^55^. However, to link our draft genomes to previous (and future) 16S rRNA gene-based studies, it is essential that all bins contain a 16S rRNA gene. Eleven draft genome bins (CHB2-4, BCD2-5, CFX2-3, OP11-2 and WS6-2) lacked a 16S rRNA gene, most probably due to co-assembly of 16S reads from different organisms into the most abundant bin within the lineage. For these bins, (near) full-length 16S rRNA gene sequences were reconstructed by mapping all reads against the Silva SSU-NR database (release 115) and extracting reads per phylum. The obtained sets were then assembled *de novo* using the CLC genomics server (v6.0.4) to obtain seed sequences, which were iteratively extended through cycles of mapping and *de novo* assembly with the ‘CLCserver_16S_extension' shell script (www.github.com/dspeth/bioinfo_scripts). In case of eight bins (CHB2-4, BCD3, BCD5, CFX3, OP11-2 and WS6-2) this extension approach led to overlap with contigs from the metagenome assembly previously assigned to a bin, linking the 16S rRNA gene to the bin. The 16S rRNA genes in the remaining three bins (BCD2, BCD4 and CFX2) were subsequently linked to bins based on comparison of the 16S rRNA and the CheckM phylogenies. This bin assignment was subsequently checked using the differential coverage profile across the eight samples.

The assembled sequences were imported in ARB and aligned against the Silva reference alignment using the integrated aligner^68^. The alignments were manually inspected and refined and 44 reference sequences from the Silva database were chosen based on phylogeny of the 23 16S rRNA gene sequences obtained from our data (Fig. 2a). The 67 aligned sequences were exported from ARB and a phylogenetic tree was constructed using FastTree2 (ref. 69) and bootstrapped using SeqBoot from the PHYLIP package^70^ and the script CompareToBootstrap.pl (http://meta.microbesonline.org/fasttree/treecmp.html).

Assembled 16S sequences were used for BLAST searches against the NCBI-nt database (December 2014), to assess the previous detection of the identified organisms (at >97% 16S rRNA gene identity) and their co-occurrence with anammox bacteria (Fig. 2c and Supplementary Table 3).

## Additional information

**Accession codes:** This Whole Genome Shotgun project has been deposited at DDBJ/EMBL/GenBank under the accessions JZEK00000000, JZQZ00000000, JZQY00000000, JYPE00000000, LLZO00000000, LLZP00000000, LMYZ00000000, JZQW00000000, LNBW00000000, LNBX00000000, LNFQ00000000, LNFR00000000, JZRA00000000, LMZR00000000, LMZS00000000, LMZT00000000, LLEU00000000, JZQX00000000, JZEL00000000, JYNZ00000000, JYPD00000000, LLEV00000000 and LMZU00000000. The versions described in this study are version JZEK01000000, JZQZ01000000, JZQY01000000, JYPE01000000, LLZO01000000, LLZP01000000, LMYZ01000000, JZQW01000000, LNBW01000000, LNBX01000000, LNFQ01000000, LNFR01000000, JZRA01000000, LMZR01000000, LMZS01000000, LMZT01000000, LLEU01000000, JZQX01000000, JZEL01000000, JYNZ01000000, JYPD01000000, LLEV01000000 and LMZU01000000.

**How to cite this article:** Speth, D. R. *et al.* Genome-based microbial ecology of anammox granules in a full-scale wastewater treatment system. *Nat. Commun.* 7:11172 doi: 10.1038/ncomms11172 (2016).

## Supplementary Material

Supplementary Information Supplementary Figures 1-6, Supplementary Tables 1-6, Supplementary Note 1

## Figures and Tables

**Figure 1:**
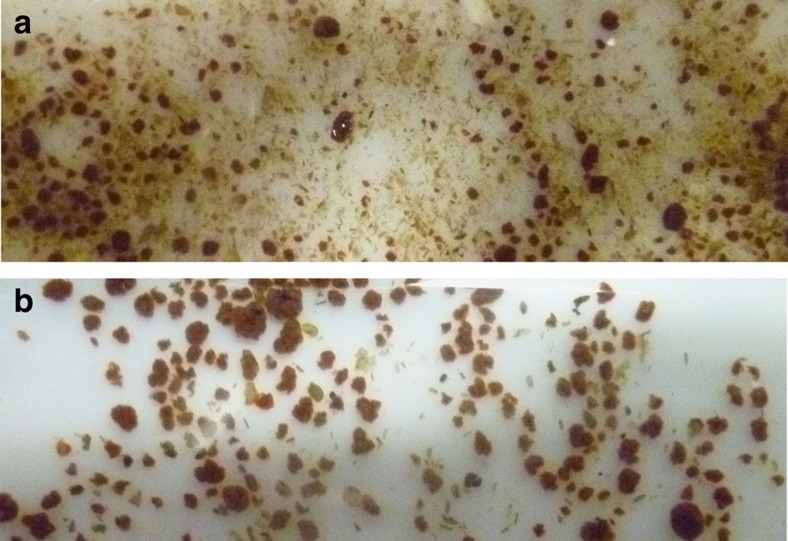
Biomass from the Olburgen PNA reactor. (**a**) Untreated sample consisting of both flocculent and granular biomass. (**b**) Washed sample containing predominantly granular biomass.

**Table 1:** Characteristics of the 23 draft genomes obtained in this study.

**Bin ID**	**Phylum**	**Number of contigs**	**Draft genome size (Mbp)**	**Single copy marker genes (out of 111)**	**Features (CDS/rRNA/tRNA)**	**Accession number**
AMX	*Planctomycetes*	86	3.9	104	3650/3/40	JZEK00000000
AOB	*Proteobacteria*	343	2.6	103	2614/3/32	JZQZ00000000
NOB	*Nitrospirae*	79	3.8	98	3743/3/31	JZQY00000000
CHB1	*Chlorobi*	24	2.5	105	2213/2/38	JYPE00000000
CHB2	*Chlorobi*	389	3.3	99	3324/2/27	LLZO00000000
CHB3	*Chlorobi*	54	2.5	101	2232/3/34	LLZP00000000
CHB4	*Chlorobi*	271	3.9	104	3593/3/39	LMYZ00000000
BCD1	*Bacteroidetes*	74	3.6	105	3000/4/30	JZQW00000000
BCD2	*Bacteroidetes*	80	3.2	104	2900/2/28	LNBW00000000
BCD3	*Bacteroidetes*	51	3.0	100	2802/2/37	LNBX00000000
BCD4	*Bacteroidetes*	72	2.1	90	2567/2/25	LNFQ00000000
BCD5	*Bacteroidetes*	118	3.6	90	3876/3/34	LNFR00000000
CFX1	*Chloroflexi*	148	4.2	103	3871/3/42	JZRA00000000
CFX2	*Chloroflexi*	74	3.0	95	3502/3/41	LMZR00000000
CFX3	*Chloroflexi*	190	3.8	96	3617/2/39	LMZS00000000
OP3	*Omnitophica* (OP3)	181	4.0	100	3759/2/33	LMZT00000000
ACD	*Acidobacteria*	65	3.0	97	2915/3/40	LLEU00000000
ATM	*Armatimonadetes* (OP10)	224	2.6	91	2630/2/31	JZQX00000000
OD1	*Parcubacteria* (OD1)	31	0.9	94	973/2/34	JZEL00000000
WS6-1	WS6	7	1.4	91	1508/2/43	JYNZ00000000
WS6-2	WS6	32	1.0	84	1140/2/29	JYPD00000000
OP11-1	*Microgenomates* (OP11)	29	0.9	82	1148/2/44	LLEV00000000
OP11-2	*Microgenomates* (OP11)	47	0.9	83	1267/2/39	LMZU00000000

**Table 2:** Performance of the Olburgen PNA reactor around the time of sampling.

**Week nr (2013)**	**COD (mg l**^**−1**^**)**		**NH4-N (mg l**^**−1**^**)**		**NO3-N (mg l**^**−1**^**)**		**Ammonium-N removed (%)**
	**In**	**Out**	**In**	**Out**	**In**	**Out**	
36	220	125	310	25	0.3	60	91.9
37	225	100	280	15	0.3	50	94.6
38	200	<100	290	20	0.3	50	93.1
39	200	<100	260	20	0.4	40	92.3
40	175	170	215	15	0.3	30	93.0
41	250	100	275	20	0.3	40	92.7
42	165	<100	250	15	2	30	94.0
43	200	<100	220	15	0.3	30	93.2
44	225	<100	260	16	0.4	40	93.8
45	210	115	260	15	0.8	40	94.2
46	180	110	265	15	0.6	45	94.3
47	240	<100	260	15	0.6	45	94.2
48	150	<100	240	15	0.6	40	93.8
49	185	<100	320	20	0.5	55	93.8
50	175	<100	250	11	0.5	45	95.6
51	155	<100	280	15	0.4	35	94.6
52	200	<100	300	12	0.6	30	96.0
Average	197.4	#	266.8	16.4	0.5	41.5	93.8
s.d.	28.9	#	28.6	3.5	0.4	9.0	1.1

The week when the samples were taken is shaded in grey.

^#^As the detection limit is 100 mg l^−1^ no average was calculated.
