# Supplementary material for: Genome-based microbial ecology of anammox granules in a full-scale wastewater treatment system
Source: Nat Commun. 2016 Mar 31;7:11172. doi: 10.1038/ncomms11172 (PMC4821891; doi:10.1038/ncomms11172)
Supplement: Supplementary Information — Supplementary Figures 1-6, Supplementary Tables 1-6, Supplementary Note 1 [file ncomms11172-s1.pdf]

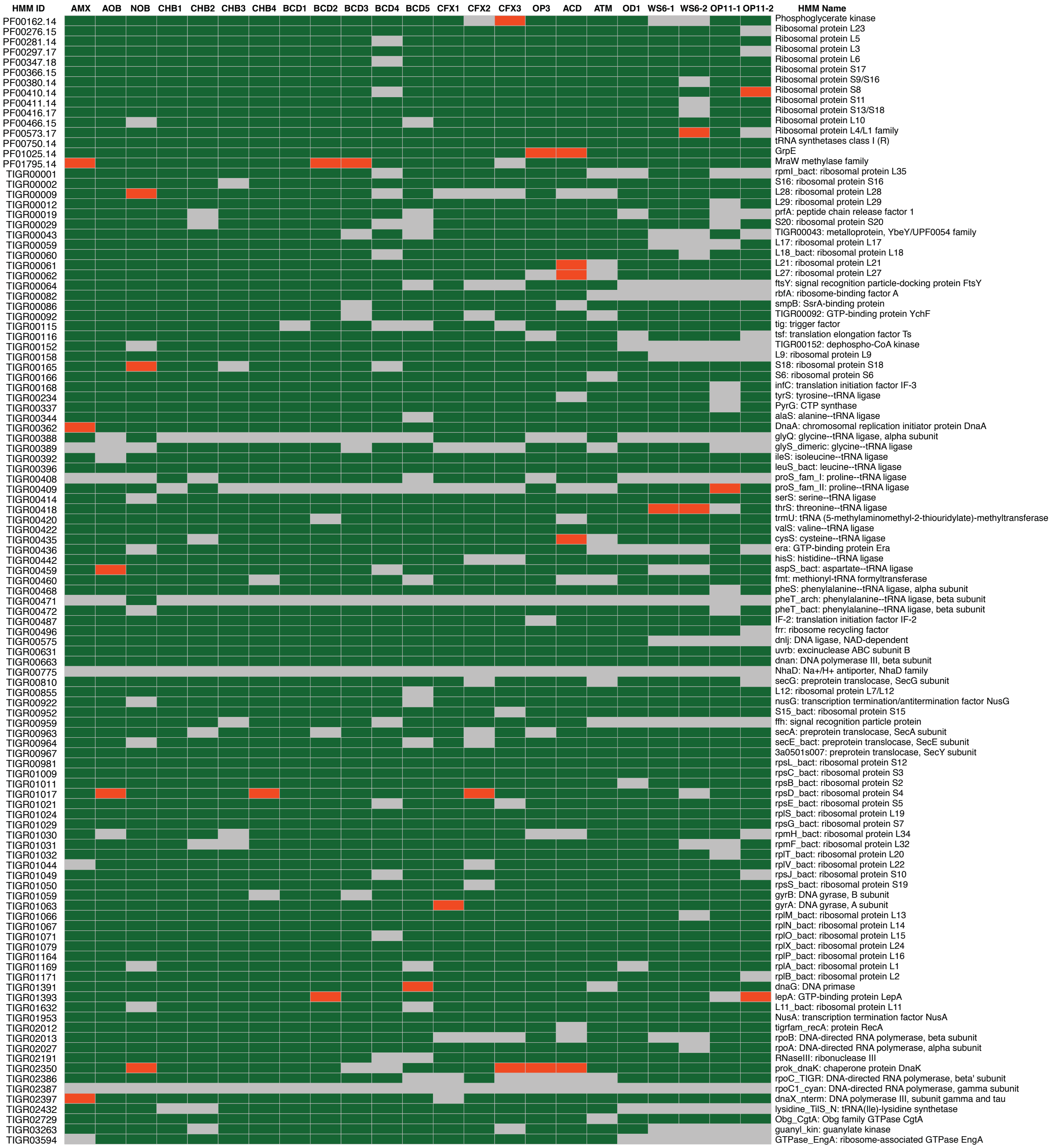

Supplementary Figure 1 presence/absence of marker genes used to estimate genome completeness.

Grey indicates a marker gene was not detected, green indicates a single copy of the marker was detected and red indicates multiple copies were detected. The set of 111 markers was taken from Dupont et al. (2012), and the HMMs used (essential.hmm) were downloaded from github.com/MadsAlbertsen/mmgenome/tree/master/scripts and searched using CheckM

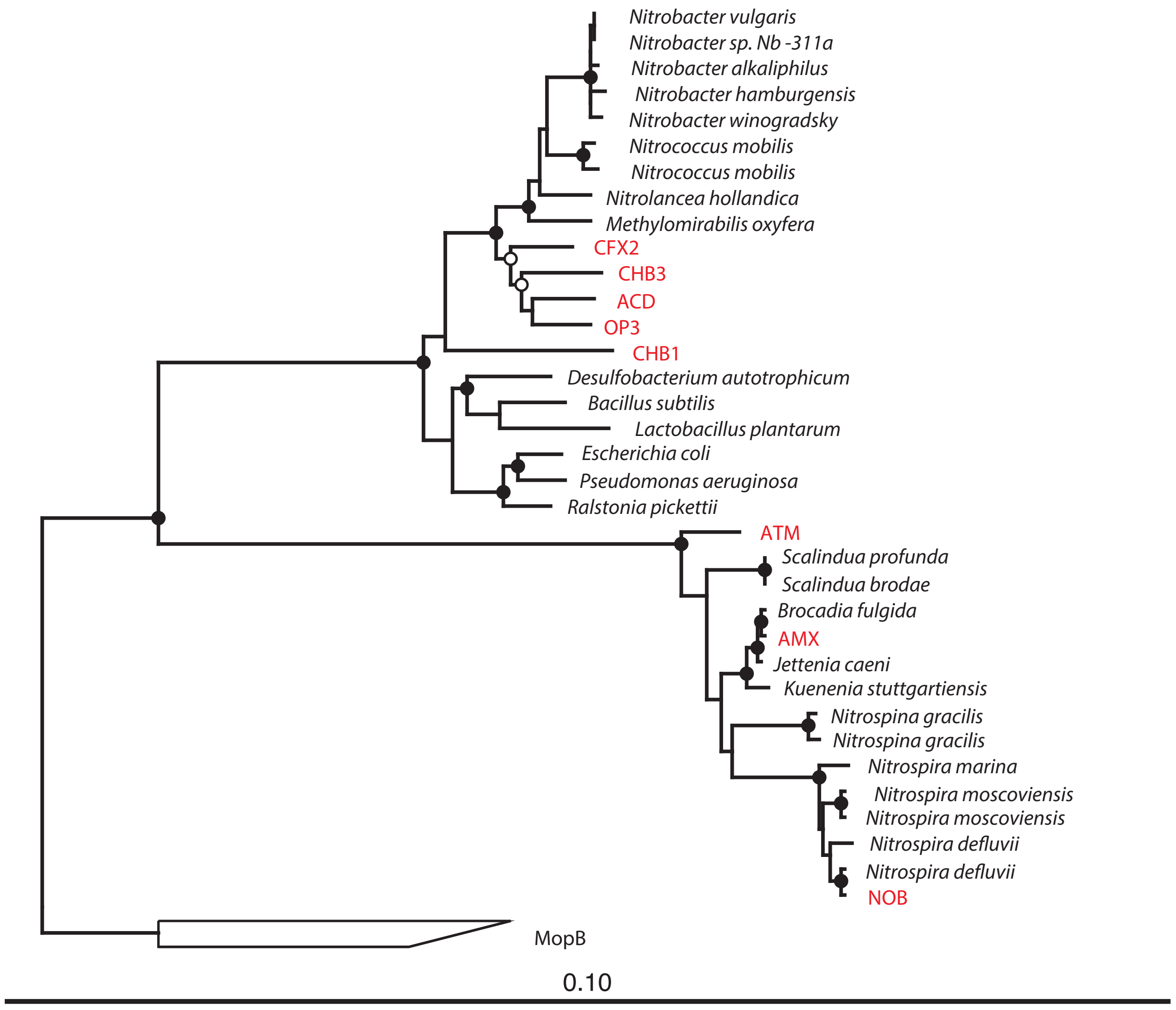

Supplementary Figure 2 Phylogeny of the large subunit of nitrate reductase (narG) and nitrate oxidoreductase (nxrA) detected in the draft genomes.

Sequences obtained in this study are indicated in red. The gene from ATM clearly clusters with AMX, NOB, and known nxrA genes. Aligned reference sequences were obtained from Lucker et al. (2013). Sequences obtained in this study were imported in ARB, aligned with the reference sequences and alignment was manually inspected and curated. 6 MopB sequences were used as outgroup. Aligned sequences were exported from ARB and a phylogenetic tree was constructed using FastTree and bootstrapped using SeqBoot from the PHYLIP package and the script CompareToBootstrap.pl. The tree was reimported in ARB for visualization.

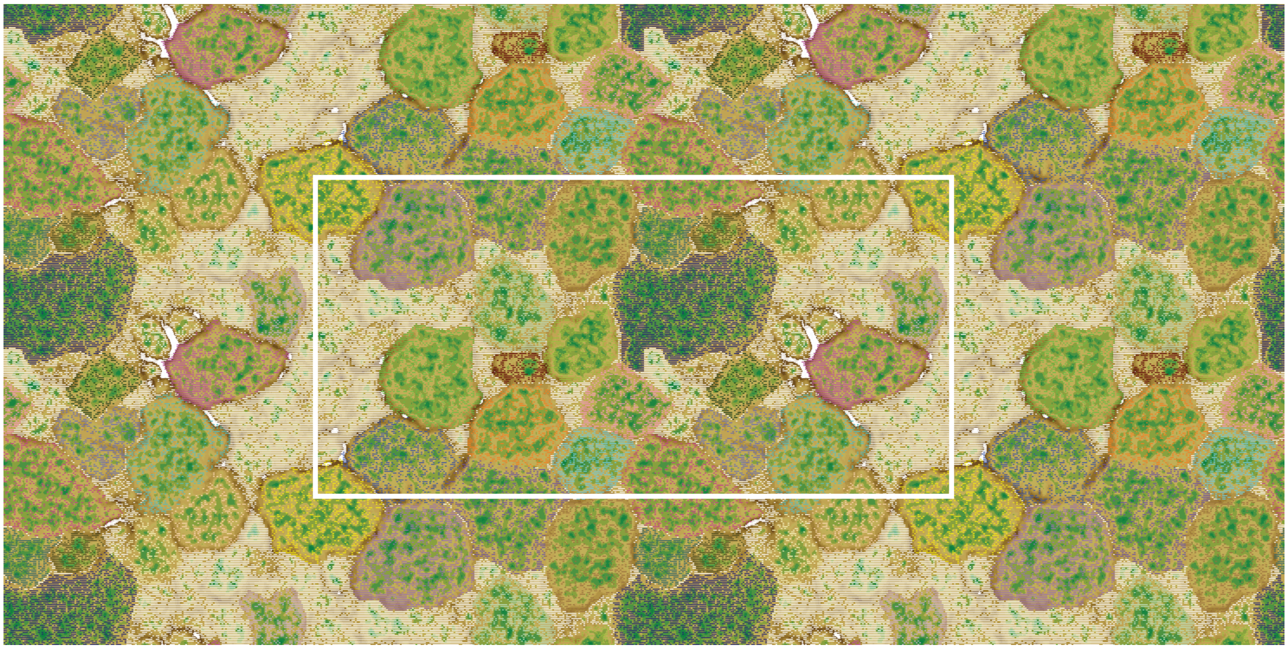

Supplementary Figure 3 tetraESOM map of all contigs longer than 5000 bp
Emergent self organizing map based on tetranucleotide frequency of all contigs longer than 5000 bp colored based on identified clusters. The clusters were extracted and used to visually guide the R binning (see methods and supplementary Fig. 4-6). The map is built up of repeating units, indicated by the white box.

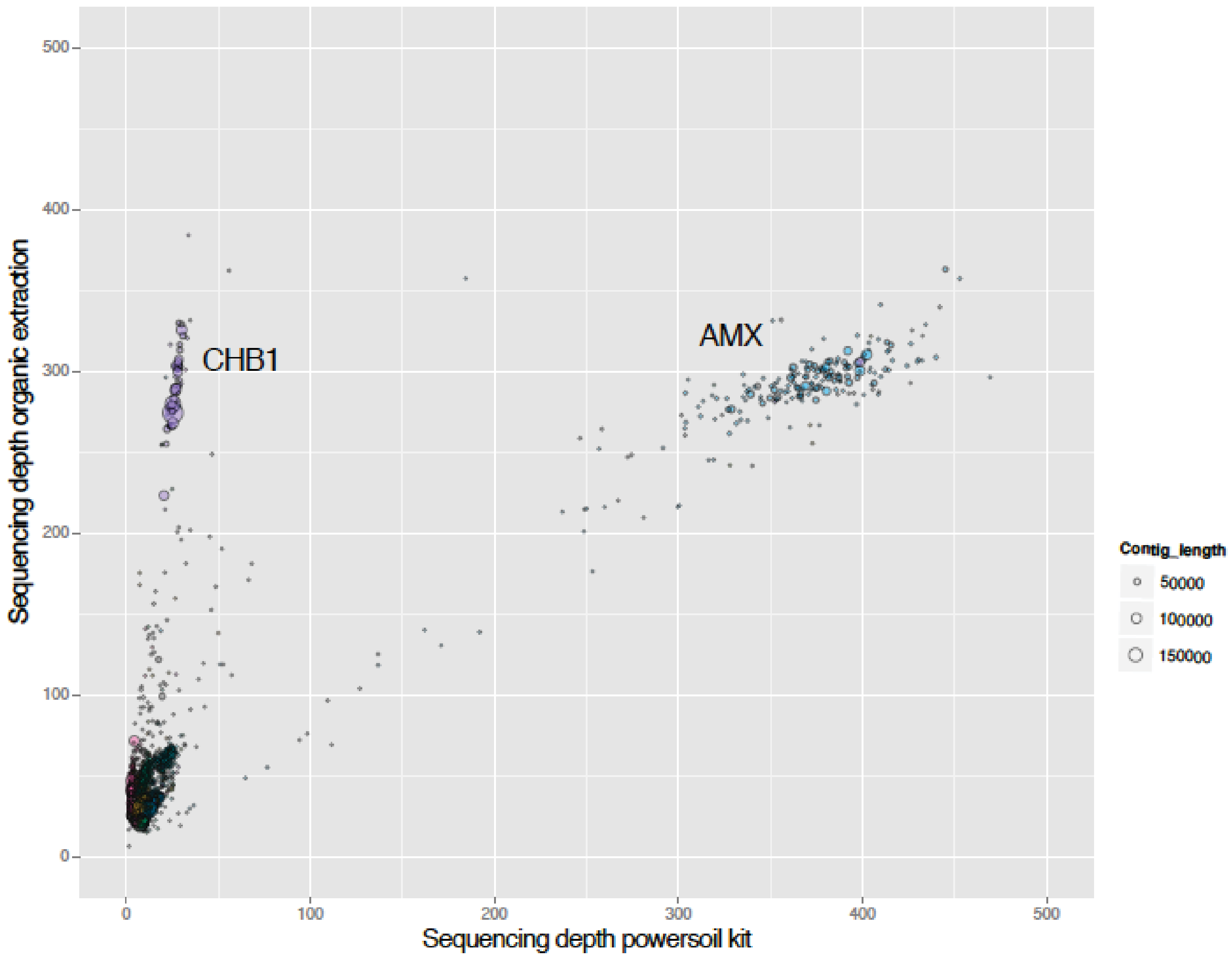

Supplementary Figure 4 Differential coverage binning using DNA isolation biases
The different effectiveness of either DNA isolation methods on AMX and CHB1 results in differences in sequencing depth which can be exploited to separate them from each other.

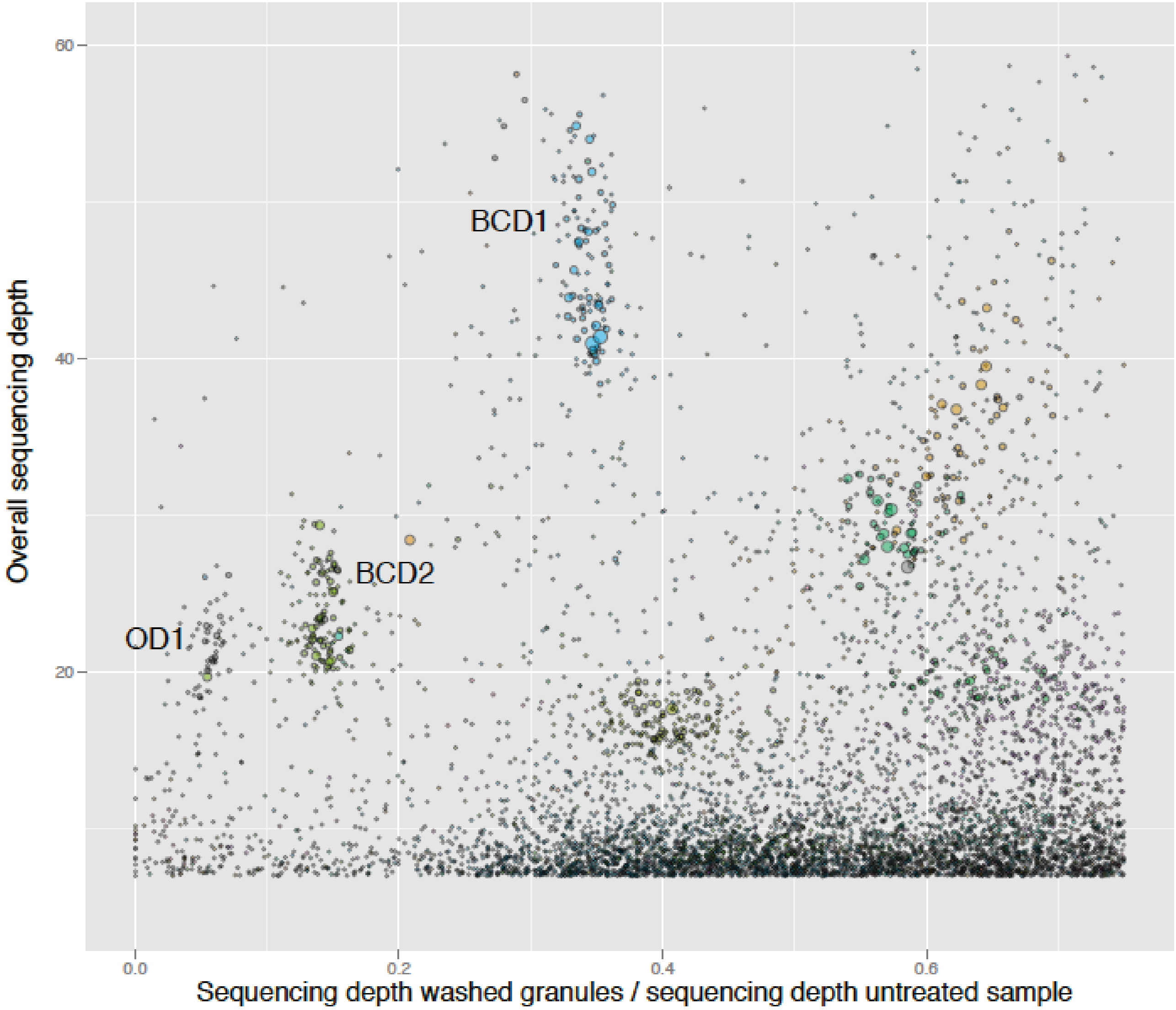

Supplementary Figure 5 Depletion of BCD1, BCD2 and OD1 in the washed granule fraction
Washing the granular biomass results in different depletion of, amongst others, BCD1, BCD2, and OD1 in the washed granules fraction. This can be exploited to separate them from the other contigs

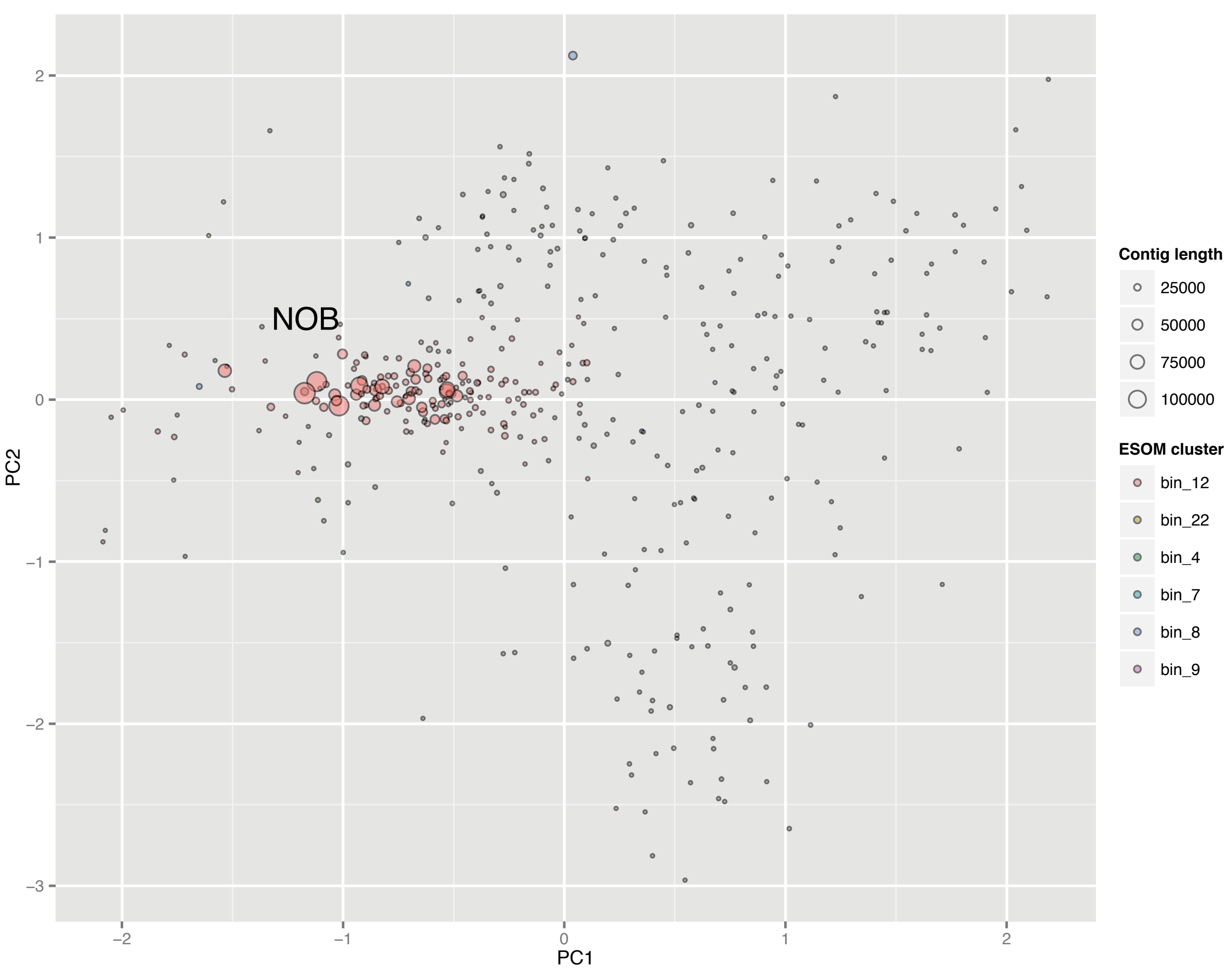

Supplementary Figure 6 refining of NOB bin
Scatterplot indicating the first two components of a tetranucleotide frequency principle component analysis of the contigs included in a preliminary NOB bin. All bins were checked using a principle component analysis of tetranucleotide frequencies of the contigs to further refine the binning.

Supplementary Table 1 Sequencing effort for this study

Sample	Heigth from base	Treatment	Extraction method	Runs	No. of reads after quality trim	Average read length after quality trim
1	3.8 m	None	Organic extraction	2	4,318,303	303 bp
2	3.8 m	None	Powersoil Kit	1	1,956,018	291 bp
3	3.8 m	Washed granules	Organic extraction	2	4,922,405	310 bp
4	3.8 m	Washed granules	Powersoil Kit	1	2,262,638	301 bp
5	1.4 m	None	Organic extraction	2	4,947,305	303 bp
6	1.4 m	None	Powersoil Kit	1	2,400,541	282 bp
7	1.4 m	Washed granules	Organic extraction	2	4,663,465	308 bp
8	1.4 m	Washed granules	Powersoil Kit	1	1,796,121	302 bp

Supplementary Table 2 Read mapping of the 8 subsamples on the obtained draft genomes (1/4)

Organic extraction
Untreated biomass

	sample point 3.8m	sample point 1.4m	average
**reads**	4318303	4947305	
**mapped**	2063596	2357388	
**% mapped**	47.79	47.65	47.72

bin	No. of reads 3.8m	No. of reads 1.4m	% of reads 3.8m	% of reads 1.4m
AMX	577108	952081	13.36	19.24
AOB	78220	198889	1.81	4.02
NOB	124178	81959	2.88	1.66
CHB1	483428	337529	11.19	6.82
CHB2	97449	88862	2.26	1.80
CHB3	25256	25738	0.58	0.52
CHB4	55569	51933	1.29	1.05
BCD1	125171	125004	2.90	2.53
BCD2	75132	74383	1.74	1.50
BCD3	50542	67923	1.17	1.37
BCD4	38847	38321	0.90	0.77
BCD5	18391	26762	0.43	0.54
CFX1	84507	57130	1.96	1.15
CFX2	34165	46017	0.79	0.93
CFX3	57699	41513	1.34	0.84
OP3	30353	40479	0.70	0.82
ACD	31750	28474	0.74	0.58
ATM	22107	17580	0.51	0.36
OD1	19415	26743	0.45	0.54
WS6-1	13152	12057	0.30	0.24
WS6-2	8111	8034	0.19	0.16
OP11-1	6912	5605	0.16	0.11
OP11-2	6134	4372	0.14	0.09
sums	2063596	2357388	47.79	47.65

Supplementary Table 2 Read mapping of the 8 subsamples on the obtained draft genomes (2/4)

Organic extraction
Washed granules

	sample point 3.8m	sample point 1.4m	average
**reads**	4922405	4663465	
**mapped**	2953326	2773692	
**% mapped**	60.00	59.48	59.74

bin	No. of reads 3.8m	No. of reads 1.4m	% of reads 3.8m	% of reads 1.4m
AMX	1197310	1003066	24.32	21.51
AOB	111769	76573	2.27	1.64
NOB	59882	67030	1.22	1.44
CHB1	736383	730604	14.96	15.67
CHB2	156930	167284	3.19	3.59
CHB3	46557	40121	0.95	0.86
CHB4	38020	48348	0.77	1.04
BCD1	52017	60134	1.06	1.29
BCD2	13547	12459	0.28	0.27
BCD3	42082	38206	0.85	0.82
BCD4	26957	26436	0.55	0.57
BCD5	23820	23209	0.48	0.50
CFX1	167717	192902	3.41	4.14
CFX2	17627	18093	0.36	0.39
CFX3	51411	73941	1.04	1.59
OP3	59581	52711	1.21	1.13
ACD	53132	52735	1.08	1.13
ATM	34564	32712	0.70	0.70
OD1	1625	794	0.03	0.02
WS6-1	25795	23273	0.52	0.50
WS6-2	15829	13242	0.32	0.28
OP11-1	11259	10361	0.23	0.22
OP11-2	9512	9458	0.19	0.20
sums	2953326	2773692	60.00	59.48

Supplementary Table 2 Read mapping of the 8 subsamples on the obtained draft genomes (3/4)

Powersoil kit
Untreated biomass

	sample point 3.8m	sample point 1.4m	average
**reads**	1956018	2400541	
**mapped**	1280603	1719985	
**% mapped**	65.47	71.65	68.56

bin	No. of reads 3.8m	No. of reads 1.4m	% of reads 3.8m	% of reads 1.4m
AMX	885622	1228827	45.28	51.19
AOB	39256	53253	2.01	2.22
NOB	20052	21668	1.03	0.90
CHB1	67651	104561	3.46	4.36
CHB2	23831	38280	1.22	1.59
CHB3	7112	9554	0.36	0.40
CHB4	12996	13364	0.66	0.56
BCD1	67870	80762	3.47	3.36
BCD2	31001	36274	1.58	1.51
BCD3	29776	33686	1.52	1.40
BCD4	6426	8366	0.33	0.35
BCD5	13796	15638	0.71	0.65
CFX1	13789	10259	0.70	0.43
CFX2	24323	18825	1.24	0.78
CFX3	8619	11660	0.44	0.49
OP3	5513	7434	0.28	0.31
ACD	5627	7517	0.29	0.31
ATM	3604	3556	0.18	0.15
OD1	7966	7719	0.41	0.32
WS6-1	2268	3416	0.12	0.14
WS6-2	1209	1884	0.06	0.08
OP11-1	1187	1825	0.06	0.08
OP11-2	1109	1657	0.06	0.07
sums	1280603	1719985	65.47	71.65

Supplementary Table 2 Read mapping of the 8 subsamples on the obtained draft genomes (4/4)

Powersoil kit
Washed granules

	sample point 3.8m	sample point 1.4m	average
**reads**	2262638	1796121	
**mapped**	1804268	1372542	
**% mapped**	79.74	76.42	78.08

bin	No. of reads 3.8m	No. of reads 1.4m	% of reads 3.8m	% of reads 1.4m
AMX	1615796	1173620	71.41	65.34
AOB	38788	42436	1.71	2.36
NOB	12452	17749	0.55	0.99
CHB1	26740	23137	1.18	1.29
CHB2	22547	19590	1.00	1.09
CHB3	7136	9189	0.32	0.51
CHB4	2503	5275	0.11	0.29
BCD1	11647	8849	0.51	0.49
BCD2	3007	1210	0.13	0.07
BCD3	13190	8806	0.58	0.49
BCD4	2630	1494	0.12	0.08
BCD5	9306	10693	0.41	0.60
CFX1	5097	10609	0.23	0.59
CFX2	6896	5697	0.30	0.32
CFX3	7883	12773	0.35	0.71
OP3	6595	9371	0.29	0.52
ACD	2969	3901	0.13	0.22
ATM	1718	2144	0.08	0.12
OD1	890	197	0.04	0.01
WS6-1	2416	2782	0.11	0.15
WS6-2	1396	879	0.06	0.05
OP11-1	1234	787	0.05	0.04
OP11-2	1432	1354	0.06	0.08
sums	1804268	1372542	79.74	76.42

Supplementary Table 3 Previous detection and closest relatives of the dominant organisms (1/2)

Bin	No. of blast hits >97% identity against NCBI-nt	blast hits >97% identity against organisms in anammox systems
AMX	37	37
AOB	233	49
NOB	86	5
CHB1	3	2
CHB2	39	3
CHB3	10	8
CHB4	10	0
BCD1	32	0
BCD2	14	2
BCD3	3	0
BCD4	4	0
BCD5	20	4
CFX1	5	4
CFX2	35	12
CFX3	2	0
OP3	18	18
ACD	34	9
ATM	15	14
OD1	0	0
WS6-1	0	0
WS6-2	0	0
OP11-1	0	0
OP11-2	0	0

Supplementary Table 3 Previous detection and closest relatives of the dominant organisms (2/2)

Bin	Closest cultured organism	16S rRNA gene identity (%)	Closest sequenced organism	16S rRNA gene identity (%)
AMX	*Brocadia sinica*	99	*Brocadia fulgida*	94
AOB	*Nitrosomonas europaea*	99	*Nitrosomonas europaea*	99
NOB	*Nitrospira defluvii*	97	*Nitrospira defluvii*	97
CHB1	*Ignavibacterium album*	85	*Ignavibacterium album*	85
CHB2	*Ignavibacterium album*	86	*Ignavibacterium album*	86
CHB3	*Melioribacter roseus*	83	*Melioribacter roseus*	83
CHB4	*Prosthecochloris aestuarii*	84	*Prosthecochloris aestuarii*	84
BCD1	*Haliscomenobacter hydrossis*	88	*Haliscomenobacter hydrossis*	88
BCD2	*Owenweeksia hongkongensis*	86	*Owenweeksia hongkongensis*	86
BCD3	*Solitalea candensis*	87	*Solitalea candensis*	87
BCD4	*Solitalea candensis*	88	*Solitalea candensis*	88
BCD5	*Chryseolinea serpens*	91	*Marivirga tractuosa*	85
CFX1	*Anaerolinea thermophila*	82	*Anaerolinea thermophila*	82
CFX2	*Litorilinea aerophila*	85	*Caldilinea aerophila*	84
CFX3	*Anaerolinea thermophila*	83	*Anaerolinea thermophila*	83
OP3	*Thermolithobacter ferrireducens*	81	*Desulfotalea psychrophila*	81
ACD	*Aridibacter famidurans*	93	*Terriglobus roseus*	83
ATM	*Fimbriimonas gingensoli*	90	*Fimbriimonas gingensoli*	90
OD1	*		*Parcubacteria bacterium* LCCS01000018	82
WS6-1	*		WS6 bacterium LBWK01000001	80
WS6-2	*		WS6 bacterium LBWK01000001	81
OP11-1	*		*Roizmanbacteria bacterium* LBPX0100022	81
OP11-2	*		*	

* = no organisms at over 80% identity

Supplementary Table 4. Trusted nitrogen cycle genes

Accession number	gene	protein
YP_003798852.1	*nxrB*	nitrate oxidoreductase subunit beta
YP_003798853.1	*nxrA*	nitrate oxidoreductase subunit alpha
NP_415742.1	*narG*	nitrate reductase, alpha subunit
NP_415743.1	*narH*	nitrate reductase, beta (Fe-S) subunit
NP_415744.1	*narJ*	delta subunit of nitrate reductase
NP_415745.1	*narI*	nitrate reductase, gamma (cytochrome b(NR)) subunit
NP_416707.4	*napB*	nitrate reductase, small, cytochrome C550 subunit, periplasmic
NP_416710.1	*napA*	nitrate reductase, periplasmic, large subunit
NP_418494.1	*nrfA*	nitrite reductase, formate-dependent, cytochrome
NP_249210.1	*nirS*	nitrite reductase
YP_003799842.1	*nirK*	copper-containing nitrite reductase
NP_249214.1	*norC*	nitric-oxide reductase (NorBC) subunit C
NP_249215.1	*norB*	nitric-oxide reductase (NorBC) subunit B
WP_011617789.1	*norZ*	nitric oxide reductase qNor type
NP_252082.1	*nosZ*	nitrous-oxide reductase
NP_252083.1	*nosD*	NosD protein
NP_252085.1	*nosY*	NosY protein
NP_252086.1	*nosL*	NosL protein
CAJ73611.1	*hzsC*	hydrazine synthase subunit C
CAJ73612.1	*hzsB*	hydrazine synthase subunit B
CAJ73613.1	*hzsA*	hydrazine synthase subunit A
NP_842054.1	*hao*	hydroxylamine oxidoreductase
NP_841016.1	*amoB*	ammonia monooxygenase subunit B
NP_841017.1	*amoA*	ammonia monooxygenase subunit A
NP_841018.1	*amoC*	ammonia monooxygenase subunit C
WP_011160148.1	*nifN*	nitrogenase molybdenum-cofactor biosynthesis protein NifN
WP_011160149.1	*nifE*	nitrogenase iron-molybdenum cofactor biosynthesis protein NifE
WP_011160150.1	*nifK*	nitrogenase molybdenum-iron protein beta chain
WP_011160151.1	*nifD*	nitrogenase molybdenum-iron protein alpha chain
WP_011160152.1	*nifH*	nitrogenase reductase
WP_011160162.1	*nifB*	FeMo cofactor biosynthesis protein NifB

Supplementary Table 5 reference genomes used for annotation of the obtained draft genomes

Reference dataset	used for annotation of	Accession numbers
Acidobacteria	ACD	NC_012483
		NC_015060
		NC_015064
		NC_015065
		NC_015057
		NC_015058
		NC_015059
		NC_016025
		NC_016024
		NC_008009
		NC_008536
		NC_016631
		NC_018014
		NC_014963
Armatimonadetes	ATM	NC_021487
		NZ_CP007139
Brocadiales	AMX	BAFH01000003
		BAFH01000002
		BAFH01000001
		BAFH01000004
		CT573071
		CT573072
		CT573073
		CT573074
		CT030148
Chloroflexi	CFX1	NC_014960
	CFX2	NC_012032
	CFX3	NC_017079
		NC_011831
		NC_010175
		NC_002936
		NC_014314
		NC_009972
		NC_009767
		NC_009523
		CP001823
		CP001824
		NC_011959
Ignavibacterium	CHB1	NC_017464
	CHB2	NC_018178
	CHB3	
	CHB4	
Nitrosomonas	AOB	NC_015222
		NC_004757
		NC_008344
		NC_015731
Nitrospira	NOB	NC_018649
		NC_017094
		NC_014355
		NC_011296
Sphingo-bacteriales	BCD1	NC_013132
	BCD2	NC_015510
	BCD3	NZ_CM001403
	BCD4	NC_016609
	BCD5	CP007035
		NC_013061
		NC_015177
		NC_015277
		NC_017770
		NC_016940

Supplementary table 6 read mapping and sequencing depth used for niche assignment

Bin	Mapped reads (untreated)	Mapped reads (washed)	Mean sequencing depth (untreated)	Mean sequencing depth (washed)	Mann Whitney U p-value	t test p-value
AMX	1549472	1781354	120.6225	140.9845	< 0.00001	< 0.00001
AOB	286007	154917	33.96467	18.72646	< 0.00001	< 0.00001
NOB	195810	101074	15.974812	8.491949	< 0.00001	< 0.00001
CHB1	825567	1174417	101.4781	145.0593	< 0.00001	< 0.00001
CHB2	182358	252663	16.76585	23.38332	< 0.00001	< 0.00001
CHB3	51544	70377	6.370658	8.793155	< 0.00001	< 0.00001
CHB4	107791	69414	8.450233	5.663588	< 0.00001	< 0.00001
BCD1	252671	90629	21.672629	7.893073	< 0.00001	< 0.00001
BCD2	151308	21184	14.63932	2.42754	< 0.00001	< 0.00001
BCD3	118941	64251	12.317571	6.683316	< 0.00001	< 0.00001
BCD4	78461	43285	10.560444	5.884025	< 0.00001	< 0.00001
BCD5	45171	37601	3.985882	3.435843	< 0.00001	< 0.00001
CFX1	143597	293034	10.49001	21.62427	< 0.00001	< 0.00001
CFX2	79611	28688	8.012961	3.106448	< 0.00001	< 0.00001
CFX3	98165	99037	8.089152	8.28197	< 0.00001	< 0.00001
OP3	70576	89689	5.404915	6.990687	< 0.00001	< 0.00001
ACD	60438	84756	6.372306	8.988227	< 0.00001	< 0.00001
ATM	39430	53493	4.86051	6.608888	< 0.00001	< 0.00001
OD1	46442	1919	15.43859	1.43055	< 0.00001	< 0.00001
WS6-1	25099	39054	5.450347	8.524052	< 0.00001	< 0.00001
WS6-2	16062	23051	4.806135	6.828341	< 0.00001	< 0.00001
OP11-1	12513	17374	4.134759	5.708186	< 0.00001	< 0.00001
OP11-2	10444	14976	3.704933	5.122653	< 0.00001	< 0.00001

Supplementary Note 1

```
### all commands run during the manual binning process of the Olburgen PNA dataset

### for an editable version, contact Daan Speth

### the file these commands were run on can be downloaded from:
### https://figshare.com/articles/Olburgen_PNA_genome_binning_source_data/1612256

> A_source_data <- read.delim(Source_data_all)
> require(vegan)
Loading required package: vegan
Loading required package: permute
Loading required package: lattice
This is vegan 2.0-9
> require(ggplot2)
Loading required package: ggplot2
> plot <- ggplot(A_source_data, aes(x=GC_content, y=Sequencing_depth, fill=bin, size=Contig_length)) + geom_point(alpha=0.5, shape=21)
> plot
> plot + ylim(0,1000)
Warning message:
Removed 15 rows containing missing values (geom_point).
> plot <- ggplot(A_source_data, aes(x=Average_coverage_total, y=Average_coverage_gran, fill=bin, size=Contig_length)) + geom_point(alpha=0.5, shape=21)
> plot
> plot + xlim(0,1000) + ylim(0,1000)
Warning message:
Removed 6 rows containing missing values (geom_point).
> plot <- ggplot(A_source_data, aes(x=Average_coverage_kit, y=Average_coverage_ctab, fill=bin, size=Contig_length)) + geom_point(alpha=0.5, shape=21)
> plot
> plot + xlim(0,1000) + ylim(0,1000)
Warning message:
Removed 6 rows containing missing values (geom_point).
> plot <- ggplot(A_source_data, aes(x=isolation_bias, y=Sequencing_depth, fill=bin, size=Contig_length)) + geom_point(alpha=0.5, shape=21)
> plot
> plot +ylim(0,1000)
Warning message:
Removed 15 rows containing missing values (geom_point).
> plot +ylim(0,250)
Warning message:
Removed 275 rows containing missing values (geom_point).
> plot +ylim(0,50)
Warning message:
Removed 1102 rows containing missing values (geom_point).
> plot +ylim(0,10) + xlim(0,3)
Warning message:
Removed 10362 rows containing missing values (geom_point).
> plot +ylim(0,1000) +xlim(0,3)
Warning message:
Removed 56 rows containing missing values (geom_point).
```

```
> a_bin1 <- subset(A_source_data, Sequencing_depth > 250 & isolation_bias > 0.9)
> plot <- ggplot(a_bin1, aes(x=GC_content, y=Sequencing_depth, fill=bin, size=Contig_length)) +
geom_point(alpha=0.5, shape=21)
> plot
> plot <- ggplot(a_bin1, aes(x=isolation_bias, y=gran_ratio, fill=bin, size=Contig_length)) + geom_point(alpha=0.5,
shape=21)
> plot
> rda <- rda(a_bin1[,13:268], data=a_bin1[,1:12], scale=T)
> scores <- scores(rda, choices = 1:5)$sites
> X_scores <- scores(rda, choices = 1:5)$sites
> rm(scores)
> a_bin1_all <- cbind(a_bin1, X_scores)
> pairs(a_bin1_all[,269:273], upper.panel=NULL)
> temp <- rbind(A_source_data, a_bin1)
> plot <- ggplot(a_bin1_all, aes(x=GC_content, y=PC2, fill=bin, size=Contig_length)) + geom_point(alpha=0.5,
shape=21)
> plot
> plot <- ggplot(a_bin1_all, aes(x=PC1, y=PC2, fill=bin, size=Contig_length)) + geom_point(alpha=0.5, shape=21)
> plot
> temp <- A_source_data[!(A_source_data$Contig %in% a_bin1$Contig),]
> plot <- ggplot(temp, aes(x=GC_content, y=Sequencing_depth, fill=bin, size=Contig_length)) +
geom_point(alpha=0.5, shape=21)
> plot
> plot <- ggplot(temp, aes(x=Average_coverage_gran, y=Average_coverage_total, fill=bin, size=Contig_length)) +
geom_point(alpha=0.5, shape=21)
> plot
> plot <- ggplot(temp, aes(x=gran_ratio, y=Sequencing_depth, fill=bin, size=Contig_length)) + geom_point(alpha=0.5,
shape=21)
> plot
> plot +xlim(0,2)
Warning message:
Removed 8948 rows containing missing values (geom_point).
> plot +xlim(1,2) + ylim(0,500)
Warning message:
Removed 63127 rows containing missing values (geom_point).
> b_bin2 <- subset(temp, gran_ratio <2 & gran_ratio > 1.25 & Sequencing_depth > 100)
> plot <- ggplot(b_bin2, aes(x=gran_ratio, y=isolation_bias, fill=bin, size=Contig_length)) + geom_point(alpha=0.5,
shape=21)
> plot
> b_bin2 <- subset(b_bin2, isolation_bias < 0.5)
> plot <- ggplot(b_bin2, aes(x=GC_content, y=Sequencing_depth, fill=bin, size=Contig_length)) +
geom_point(alpha=0.5, shape=21)
> plot
> rda <- rda(b_bin2[,13:268], data=b_bin2[,1:12], scale=T)
> X_scores <- scores(rda, choices = 1:5)$sites
> b_bin2_all <- cbind(b_bin2, X_scores)
> pairs(b_bin2_all[,269:273], upper.panel=NULL)
> plot <- ggplot(b_bin2_all, aes(x=PC1, y=Sequencing_depth, fill=bin, size=Contig_length)) + geom_point(alpha=0.5,
shape=21)
> plot
> plot <- ggplot(b_bin2_all, aes(x=PC1, y=PC2, fill=bin, size=Contig_length)) + geom_point(alpha=0.5, shape=21)
> plot
> b_bin2_all <- subset(b_bin2_all, PC1 > -2 & PC2 > -2)
> plot <- ggplot(b_bin2_all, aes(x=GC_content, y=Sequencing_depth, fill=bin, size=Contig_length)) +
```

```
geom_point(alpha=0.5, shape=21)
> plot
> plot <- ggplot(b_bin2_all, aes(x=PC1, y=PC2, fill=bin, size=Contig_length)) + geom_point(alpha=0.5, shape=21)
> plot
> plot <- ggplot(b_bin2_all, aes(x=gran_ratio, y=isolation_bias, fill=bin, size=Contig_length)) +
geom_point(alpha=0.5, shape=21)
> plot
> b_bin2_all_t <- subset(b_bin2_all, gran_ratio >1.4 & gran_ratio < 1.65 & isolation_bias < 0.112)
> plot <- ggplot(b_bin2_all_t, aes(x=PC1, y=PC2, fill=bin, size=Contig_length)) + geom_point(alpha=0.5, shape=21)
> plot
> rm(b_bin2_all_t)
> plot <- ggplot(b_bin2_all, aes(x=gran_ratio, y=isolation_bias, fill=bin, size=Contig_length)) +
geom_point(alpha=0.5, shape=21)
> plot
> b_bin2_all <- subset(b_bin2_all, isolation_bias < 0.112)
> temp_t <- temp[!(temp$Contig %in% b_bin2_all$Contig),]
> temp <- temp[!(temp$Contig %in% b_bin2_all$Contig),]
> rm(temp_t)
> plot <- ggplot(temp, aes(x=GC_content, y=Sequencing_depth, fill=bin, size=Contig_length)) +
geom_point(alpha=0.5, shape=21)
> plot
> plot + ylim(0,200)
Warning message:
Removed 22 rows containing missing values (geom_point).
> plot <- ggplot(temp, aes(x=Average_coverage_gran, y=Average_coverage_total, fill=bin, size=Contig_length)) +
geom_point(alpha=0.5, shape=21)
> plot
> plot +xlim(0,100) + ylim(0,100)
Warning message:
Removed 49 rows containing missing values (geom_point).
> plot <- ggplot(temp, aes(x=gran-ratio, y=Sequencing_depth, fill=bin, size=Contig_length)) + geom_point(alpha=0.5,
shape=21)
> plot <- ggplot(temp, aes(x=gran_ratio, y=Sequencing_depth, fill=bin, size=Contig_length)) + geom_point(alpha=0.5,
shape=21)
> plot
> plot + xlim(0,5)
Warning message:
Removed 822 rows containing missing values (geom_point).
> plot + xlim(0,1.5)
Warning message:
Removed 19609 rows containing missing values (geom_point).
> plot + xlim(0,1.5) + ylim(0,200)
Warning message:
Removed 19624 rows containing missing values (geom_point).
> c_bin3 <- subset(temp, gran_ratio > 0.45 & gran_ratio < 1.2 & Sequencing_depth > 40)
> plot <- ggplot(temp, aes(x=gran_ratio, y=isolation_bias, fill=bin, size=Contig_length)) + geom_point(alpha=0.5,
shape=21)
> plot
> plot <- ggplot(c_bin3, aes(x=gran_ratio, y=isolation_bias, fill=bin, size=Contig_length)) + geom_point(alpha=0.5,
shape=21)
> plot
> rda <- rda(c_bin3[,13:268], data=c_bin3[,1:12], scale=T)
> X_scores <- scores(rda, choices = 1:5)$sites
> c_bin3_all <- cbind(c_bin3, X_scores)
```

```
> pairs(c_bin3_all[269:273], upper.panel=NULL)
> plot <- ggplot(c_bin3_all, aes(x=PC1, y=PC2, fill=bin, size=Contig_length)) + geom_point(alpha=0.5, shape=21)
> plot
> plot <- ggplot(c_bin3_all, aes(x=GC_content, y=Sequencing_depth, fill=bin, size=Contig_length)) +
geom_point(alpha=0.5, shape=21)
> plot
> c_bin3_all <- subset(c_bin3_all, PC1 > -1 & PC2 < 1)
> plot <- ggplot(c_bin3_all, aes(x=GC_content, y=Sequencing_depth, fill=bin, size=Contig_length)) +
geom_point(alpha=0.5, shape=21)
> plot
> plot <- ggplot(c_bin3_all, aes(x=isolation_bias, y=gran_ratio, fill=bin, size=Contig_length)) +
geom_point(alpha=0.5, shape=21)
> plot
> c_bin3_all <- subset(c_bin3_all, isolation_bias < 0.8 & gran_ratio < 1.0)
> plot <- ggplot(c_bin3_all, aes(x=isolation_bias, y=gran_ratio, fill=bin, size=Contig_length)) +
geom_point(alpha=0.5, shape=21)
> plot
> c_bin3 <- c_bin3_all[-c(269:273)]
> View(c_bin3)
> View(c_bin3_all)
> rda <- rda(c_bin3[,13:268], data=c_bin3[,1:12], scale=T)
> X_scores <- scores(rda, choices = 1:5)$sites
> c_bin3_all <- cbind(c_bin3, X_scores)
> pairs(c_bin3_all[269:273], upper.panel=NULL)
> plot <- ggplot(c_bin3_all, aes(x=PC1, y=PC2, fill=bin, size=Contig_length)) + geom_point(alpha=0.5, shape=21)
> plot
> c_bin3_all <- subset(c_bin3_all, PC2 < 2)
> c_bin3_all <- subset(c_bin3_all, PC1 < 2)
> plot <- ggplot(c_bin3_all, aes(x=isolation_bias, y=Sequencing_depth, fill=bin, size=Contig_length)) +
geom_point(alpha=0.5, shape=21)
> plot
> plot + ylim(0,100)
Warning message:
Removed 6 rows containing missing values (geom_point).
> c_bin3_all <- subset(c_bin3_all, isolation_bias < 0.5)
> plot <- ggplot(c_bin3_all, aes(x=PC1, y=PC2, fill=bin, size=Contig_length)) + geom_point(alpha=0.5, shape=21)
> plot
> plot <- ggplot(c_bin3_all, aes(x=isolation_bias, y=gran_ratio, fill=bin, size=Contig_length)) +
geom_point(alpha=0.5, shape=21)
> plot
> temp <- temp[!(temp$Contig %in% c_bin3_all$Contig), ]
> plot <- ggplot(temp, aes(x=gran_ratio, y=Sequencing_depth, fill=bin, size=Contig_length)) + geom_point(alpha=0.5,
shape=21)
> plot
> plot + xlim(0,10)
Warning message:
Removed 152 rows containing missing values (geom_point).
> plot + xlim(0,3)
Warning message:
Removed 2920 rows containing missing values (geom_point).
> plot + xlim(1,2) + ylim(0,200)
Warning message:
Removed 62827 rows containing missing values (geom_point).
> plot + xlim(1,2) + ylim(0,100)
```

```
Warning message:
Removed 62843 rows containing missing values (geom_point).
> d_bin4 <- subset(temp, gran_ratio > 1 & gran_ratio < 2 & Sequencing_depth > 25)
> plot <- ggplot(temp, aes(x=gran_ratio, y=isolation_bias, fill=bin, size=Contig_length)) + geom_point(alpha=0.5,
shape=21)
> plot
> plot <- ggplot(d_bin4, aes(x=gran_ratio, y=isolation_bias, fill=bin, size=Contig_length)) + geom_point(alpha=0.5,
shape=21)
> plot
> d_bin4 <- subset(d_bin4, isolation_bias < 0.4 & gran_ratio < 1.75)
> plot <- ggplot(d_bin4, aes(x=gran_ratio, y=isolation_bias, fill=bin, size=Contig_length)) + geom_point(alpha=0.5,
shape=21)
> plot
> d_bin4 <- subset(d_bin4, gran_ratio > 1.3)
> rda <- rda(d_bin4[,13:268], data=d_bin4[,1:12], scale=T)
> X_scores <- scores(rda, choices = 1:5)$sites
> d_bin4_all <- cbind(d_bin4, X_scores)
> pairs(d_bin4_all[269:273], upper.panel=NULL)
> plot <- ggplot(d_bin4_all, aes(x=PC1, y=PC3, fill=bin, size=Contig_length)) + geom_point(alpha=0.5, shape=21)
> plot
> plot + xlim(0,1)
Warning message:
Removed 438 rows containing missing values (geom_point).
> plot + xlim(-1,1)
Warning message:
Removed 74 rows containing missing values (geom_point).
> d_bin4_all <- subset(d_bin4_all, PC1 < 0.25)
> plot <- ggplot(d_bin4_all, aes(x=gran_ratio, y=isolation_bias, fill=bin, size=Contig_length)) +
geom_point(alpha=0.5, shape=21)
> plot
> plot <- ggplot(d_bin4_all, aes(x=GC_content, y=Sequencing_depth, fill=bin, size=Contig_length)) +
geom_point(alpha=0.5, shape=21)
> plot
> temp <- temp[!(temp$Contig %in% d_bin4_all$Contig), ]
> plot <- ggplot(temp, aes(x=GC_content, y=Sequencing_depth, fill=bin, size=Contig_length)) +
geom_point(alpha=0.5, shape=21)
> plot
> plot + ylim(0,100)
Warning message:
Removed 60 rows containing missing values (geom_point).
> plot <- ggplot(temp, aes(x=gran_ratio, y=Sequencing_depth, fill=bin, size=Contig_length)) + geom_point(alpha=0.5,
shape=21)
> plot
> plot <- ggplot(temp, aes(x=isolation_bias, y=Sequencing_depth, fill=bin, size=Contig_length)) +
geom_point(alpha=0.5, shape=21)
> plot
> plot <- ggplot(temp, aes(x=(1/isolation_bias), y=Sequencing_depth, fill=bin, size=Contig_length)) +
geom_point(alpha=0.5, shape=21)
> plot
> plot + xlim(0,20)
Warning message:
Removed 4746 rows containing missing values (geom_point).
> plot + xlim(0,20) + ylim(0,100)
Warning message:
```

```
Removed 4805 rows containing missing values (geom_point).
> plot <- ggplot(temp, aes(x=isolation_bias, y=Sequencing_depth, fill=bin, size=Contig_length)) + geom_point(alpha=0.5, shape=21)
> plot + ylim(0,100)
Warning message:
Removed 60 rows containing missing values (geom_point).
> plot + ylim(0,100) + xlim(0,1)
Warning message:
Removed 1781 rows containing missing values (geom_point).
> plot + ylim(0,100) + xlim(0.25,1)
Warning message:
Removed 62012 rows containing missing values (geom_point).
> e_bin5 <- subset(temp, isolation_bias > 0.25 & Sequencing_depth > 25)
> plot <- ggplot(e_bin5, aes(x=isolation_bias, y=gran_ratio, fill=bin, size=Contig_length)) + geom_point(alpha=0.5, shape=21)
> plot
> e_bin5 <- subset(e_bin5, gran_ratio < 0.5 & gran_ratio > 0.2)
> plot <- ggplot(e_bin5, aes(x=isolation_bias, y=gran_ratio, fill=bin, size=Contig_length)) + geom_point(alpha=0.5, shape=21)
> plot
> rda <- rda(e_bin5[,13:268], data=e_bin5[,1:12], scale=T)
> X_scores <- scores(rda, choices = 1:5)$sites
> e_bin5_all <- cbind(e_bin5, X_scores)
> pairs(e_bin5_all[,269:273], upper.panel=NULL)
> plot <- ggplot(e_bin5_all, aes(x=PC1, y=PC2, fill=bin, size=Contig_length)) + geom_point(alpha=0.5, shape=21)
> plot
> plot + ylim(-1,1)
Warning message:
Removed 54 rows containing missing values (geom_point).
> e_bin5_all <- subset(e_bin5_all, PC2 > -0.2)
> plot <- ggplot(e_bin5_all, aes(x=PC1, y=PC2, fill=bin, size=Contig_length)) + geom_point(alpha=0.5, shape=21)
> plot
> plot <- ggplot(e_bin5_all, aes(x=isolation_bias, y=gran_ratio, fill=bin, size=Contig_length)) + geom_point(alpha=0.5, shape=21)
> plot
> plot <- ggplot(e_bin5_all, aes(x=GC_content, y=Sequencing_depth, fill=bin, size=Contig_length)) + geom_point(alpha=0.5, shape=21)
> plot
> temp <- temp[!(temp$Contig %in% e_bin5_all$Contig), ]
> plot <- ggplot(temp, aes(x=GC_content, y=Sequencing_depth, fill=bin, size=Contig_length)) + geom_point(alpha=0.5, shape=21)
> plot
> plot + ylim(0,100)
Warning message:
Removed 54 rows containing missing values (geom_point).
> plot <- ggplot(temp, aes(x=isolation_bias, y=Sequencing_depth, fill=bin, size=Contig_length)) + geom_point(alpha=0.5, shape=21)
> plot
> plot <- ggplot(temp, aes(x=gran_ratio, y=Sequencing_depth, fill=bin, size=Contig_length)) + geom_point(alpha=0.5, shape=21)
> plot
> plot + xlim(0,5)
Warning message:
Removed 822 rows containing missing values (geom_point).
```

```
> plot + xlim(0,5) +ylim(0,100)
Warning message:
Removed 876 rows containing missing values (geom_point).
> plot + xlim(1,4) +ylim(0,100)
Warning message:
Removed 55135 rows containing missing values (geom_point).
> plot + xlim(1,3) +ylim(0,100)
Warning message:
Removed 56653 rows containing missing values (geom_point).
> f_bin6 <- subset(temp, gran_ratio > 1.7 & Sequencing_depth > 10)
> plot <- ggplot(f_bin6, aes(x=gran_ratio, y=isolation_bias, fill=bin, size=Contig_length)) + geom_point(alpha=0.5,
shape=21)
> plot
> plot <- ggplot(f_bin6, aes(x=gran_ratio, y=(1/isolation_bias), fill=bin, size=Contig_length)) +
geom_point(alpha=0.5, shape=21)
> plot
> plot +ylim(0,20)
Warning message:
Removed 47 rows containing missing values (geom_point).
> plot <- ggplot(f_bin6, aes(x=gran_ratio, y=isolation_bias, fill=bin, size=Contig_length)) + geom_point(alpha=0.5,
shape=21)
> plot +ylim(0,0.5)
Warning message:
Removed 18 rows containing missing values (geom_point).
> plot +ylim(0,0.5) + xlim(0,4)
Warning message:
Removed 23 rows containing missing values (geom_point).
> plot <- ggplot(f_bin6, aes(x=isolation_bias, y=Sequencing_depth, fill=bin, size=Contig_length)) +
geom_point(alpha=0.5, shape=21)
> plot
> plot + ylim(0,100)
Warning message:
Removed 19 rows containing missing values (geom_point).
> rda <- rda(f_bin6[,13:268], data=f_bin6[,1:12], scale=T)
> X_scores <- scores(rda, choices = 1:5)$sites
> f_bin6_all <- cbind(f_bin6, X_scores)
> pairs(f_bin6_all[,269:273], upper.panel=NULL)
> plot <- ggplot(f_bin6_all, aes(x=PC1, y=PC2, fill=bin, size=Contig_length)) + geom_point(alpha=0.5, shape=21)
> plot
> plot <- ggplot(f_bin6_all, aes(x=PC1, y=Sequencing_depth, fill=bin, size=Contig_length)) + geom_point(alpha=0.5,
shape=21)
> plot
> f_bin6_all <- subset(f_bin6_all, PC1 < 0)
> plot <- ggplot(f_bin6_all, aes(x=PC1, y=PC2, fill=bin, size=Contig_length)) + geom_point(alpha=0.5, shape=21)
> plot
> f_bin6_all <- subset(f_bin6_all, PC2 > -1)
> plot <- ggplot(f_bin6_all, aes(x=gran_ratio, y=isolation_bias, fill=bin, size=Contig_length)) +
geom_point(alpha=0.5, shape=21)
> plot
> plot <- ggplot(f_bin6_all, aes(x=PC2, y=isolation_bias, fill=bin, size=Contig_length)) + geom_point(alpha=0.5,
shape=21)
> plot
> f_bin6 <- f_bin6_all[-c(269:273)]
> rda <- rda(f_bin6[,13:268], data=f_bin6[,1:12], scale=T)
```

```
> X_scores <- scores(rda, choices = 1:5)$sites
> f_bin6_all <- cbind(f_bin6, X_scores)
> pairs(f_bin6_all[,269:273], upper.panel=NULL)
> plot <- ggplot(f_bin6_all, aes(x=PC1, y=PC2, fill=bin, size=Contig_length)) + geom_point(alpha=0.5, shape=21)
> plot
> plot <- ggplot(f_bin6_all, aes(x=PC1, y=isolation_bias, fill=bin, size=Contig_length)) + geom_point(alpha=0.5, shape=21)
> plot
> plot <- ggplot(f_bin6_all, aes(x=PC1, y=Sequencing_depth, fill=bin, size=Contig_length)) + geom_point(alpha=0.5, shape=21)
> plot
> plot + ylim(0,100)
Warning message:
Removed 6 rows containing missing values (geom_point).
> f_bin6_all <- subset(f_bin6_all, PC2 > -0.5 & PC1 < 1)
> plot <- ggplot(f_bin6_all, aes(x=PC2, y=PC3, fill=bin, size=Contig_length)) + geom_point(alpha=0.5, shape=21)
> plot
> f_bin6_all <- subset(f_bin6_all, PC2 < 1.5)
> f_bin6 <- f_bin6_all[-c(269:273)]
> rda <- rda(f_bin6[,13:268], data=f_bin6[,1:12], scale=T)
> X_scores <- scores(rda, choices = 1:5)$sites
> f_bin6_all <- cbind(f_bin6, X_scores)
> pairs(f_bin6_all[,269:273], upper.panel=NULL)
> plot <- ggplot(f_bin6_all, aes(x=PC2, y=PC3, fill=bin, size=Contig_length)) + geom_point(alpha=0.5, shape=21)
> plot
> f_bin6_all <- subset(f_bin6_all, PC2 > -1.5)
> plot <- ggplot(f_bin6_all, aes(x=PC1, y=PC2, fill=bin, size=Contig_length)) + geom_point(alpha=0.5, shape=21)
> plot
> f_bin6_all <- subset(f_bin6_all, PC2 < 1 & PC1 < 1)
> plot <- ggplot(f_bin6_all, aes(x=gran_ratio, y=isolation_bias, fill=bin, size=Contig_length)) +
geom_point(alpha=0.5, shape=21)
> plot
> plot <- ggplot(f_bin6_all, aes(x=PC2, y=isolation_bias, fill=bin, size=Contig_length)) + geom_point(alpha=0.5, shape=21)
> plot
> plot <- ggplot(f_bin6_all, aes(x=GC_content, y=Sequencing_depth, fill=bin, size=Contig_length)) +
geom_point(alpha=0.5, shape=21)
> plot
> f_bin6_all <- subset(f_bin6_all, Sequencing_depth > 20 | GC_content > 0.64)
> plot <- ggplot(f_bin6_all, aes(x=GC_content, y=Sequencing_depth, fill=bin, size=Contig_length)) +
geom_point(alpha=0.5, shape=21)
> plot
> f_bin6 <- f_bin6_all[-c(269:273)]
> rda <- rda(f_bin6[,13:268], data=f_bin6[,1:12], scale=T)
> X_scores <- scores(rda, choices = 1:5)$sites
> f_bin6_all <- cbind(f_bin6, X_scores)
> pairs(f_bin6_all[,269:273], upper.panel=NULL)
> plot <- ggplot(f_bin6_all, aes(x=PC1, y=PC2, fill=bin, size=Contig_length)) + geom_point(alpha=0.5, shape=21)
> plot
> plot <- ggplot(f_bin6_all, aes(x=isolation_bias, y=gran_ratio, fill=bin, size=Contig_length)) +
geom_point(alpha=0.5, shape=21)
> plot
> plot <- ggplot(f_bin6_all, aes(x=GC_content, y=Sequencing_depth, fill=bin, size=Contig_length)) +
geom_point(alpha=0.5, shape=21)
```

```
> plot
> temp <- temp[!(temp$Contig %in% f_bin6_all$Contig), ]
> plot <- ggplot(temp, aes(x=gran_ratio, y=Sequencing_depth, fill=bin, size=Contig_length)) + geom_point(alpha=0.5,
shape=21)
> plot
> plot + xlim(0,5)
Warning message:
Removed 822 rows containing missing values (geom_point).
> plot + xlim(0,5) + ylim(0,100)
Warning message:
Removed 876 rows containing missing values (geom_point).
> plot + xlim(0,0.5) + ylim(0,100)
Warning message:
Removed 66539 rows containing missing values (geom_point).
> plot + xlim(0,0.2) + ylim(0,100)
Warning message:
Removed 83097 rows containing missing values (geom_point).
> g_bin7 <- subset(temp, gran_ratio < 0.13 & Sequencing_depth > 5)
> plot <- ggplot(g_bin7, aes(x=gran_ratio, y=isolation_bias, fill=bin, size=Contig_length)) + geom_point(alpha=0.5,
shape=21)
> plot
> g_bin7 <- subset(g_bin7, isolation_bias < 0.7)
> plot <- ggplot(g_bin7, aes(x=gran_ratio, y=isolation_bias, fill=bin, size=Contig_length)) + geom_point(alpha=0.5,
shape=21)
> plot
> g_bin7 <- subset(g_bin7, isolation_bias > 0.2 & gran_ratio > 0.01)
> plot <- ggplot(g_bin7, aes(x=gran_ratio, y=isolation_bias, fill=bin, size=Contig_length)) + geom_point(alpha=0.5,
shape=21)
> plot
> plot <- ggplot(g_bin7, aes(x=GC_content, y=Sequencing_depth, fill=bin, size=Contig_length)) +
geom_point(alpha=0.5, shape=21)
> plot
> g_bin7 <- subset(g_bin7, Sequencing_depth > 10)
> plot <- ggplot(g_bin7, aes(x=gran_ratio, y=isolation_bias, fill=bin, size=Contig_length)) + geom_point(alpha=0.5,
shape=21)
> plot
> rda <- rda(g_bin7[,13:268], data=g_bin7[,1:12], scale=T)
> X_scores <- scores(rda, choices = 1:5)$sites
> g_bin7_all <- cbind
> g_bin7_all <- cbind(g_bin7, X_scores)
> pairs(g_bin7_all[,269:273], upper.panel=NULL)
> plot <- ggplot(g_bin7_all, aes(x=PC1, y=PC2, fill=bin, size=Contig_length)) + geom_point(alpha=0.5, shape=21)
> plot
> g_bin7_all <- subset(g_bin7_all, PC1 < 0.8)
> plot <- ggplot(g_bin7_all, aes(x=PC3, y=PC2, fill=bin, size=Contig_length)) + geom_point(alpha=0.5, shape=21)
> plot
> g_bin7_all <- subset(g_bin7_all, PC2 < 1)
> g_bin7_all <- subset(g_bin7_all, PC3 < 1)
> plot <- ggplot(g_bin7_all, aes(x=isolation_bias, y=gran_ratio, fill=bin, size=Contig_length)) +
geom_point(alpha=0.5, shape=21)
> plot
> temp <- temp[!(temp$Contig %in% g_bin7_all$Contig), ]
> plot <- ggplot(temp, aes(x=gran_ratio, y=Sequencing_depth, fill=bin, size=Contig_length)) + geom_point(alpha=0.5,
shape=21)
```

```
> plot + xlim(0,0.5) + ylim(0,100)
Warning message:
Removed 66539 rows containing missing values (geom_point).
> h_bin8 <- subset(temp, gran_ratio < 0.3 & Sequencing_depth > 10)
> plot <- ggplot(temp, aes(x=gran_ratio, y=isolation_bias, fill=bin, size=Contig_length)) + geom_point(alpha=0.5,
shape=21)
> plot
> plot <- ggplot(h_bin8, aes(x=gran_ratio, y=isolation_bias, fill=bin, size=Contig_length)) + geom_point(alpha=0.5,
shape=21)
> plot
> h_bin8 <- subset(h_bin8, gran_ratio < 0.25 & isolation_bias > 0.2)
> plot <- ggplot(h_bin8, aes(x=gran_ratio, y=isolation_bias, fill=bin, size=Contig_length)) + geom_point(alpha=0.5,
shape=21)
> plot
> h_bin8 <- subset(temp, gran_ratio < 0.2 & gran_ratio > 0.1)
> h_bin8 <- subset(temp, gran_ratio < 0.3 & Sequencing_depth > 10)
> h_bin8 <- subset(h_bin8, gran_ratio < 0.25 & isolation_bias > 0.2)
> h_bin8 <- subset(h_bin8, gran_ratio < 0.2 & gran_ratio > 0.1)
> plot <- ggplot(h_bin8, aes(x=gran_ratio, y=isolation_bias, fill=bin, size=Contig_length)) + geom_point(alpha=0.5,
shape=21)
> plot
> rda <- rda(h_bin8[,13:268], data=h_bin8[,1:12], scale=T)
> X_scores <- scores(rda, choices = 1:5)$sites
> h_bin8_all <- cbind(h_bin8,X_scores)
> pairs(h_bin8_all[,269:273], upper.panel=NULL)
> plot <- ggplot(h_bin8_all, aes(x=PC1, y=PC2, fill=bin, size=Contig_length)) + geom_point(alpha=0.5, shape=21)
> plot
> plot <- ggplot(h_bin8_all, aes(x=PC2, y=PC3, fill=bin, size=Contig_length)) + geom_point(alpha=0.5, shape=21)
> plot
> plot <- ggplot(h_bin8_all, aes(x=GC_content, y=Sequencing_depth, fill=bin, size=Contig_length)) +
geom_point(alpha=0.5, shape=21)
> plot
> h_bin8_all <- subset(h_bin8_all, PC1 < 1)
> plot <- ggplot(h_bin8_all, aes(x=GC_content, y=Sequencing_depth, fill=bin, size=Contig_length)) +
geom_point(alpha=0.5, shape=21)
> plot
> plot <- ggplot(h_bin8_all, aes(x=PC1, y=PC2, fill=bin, size=Contig_length)) + geom_point(alpha=0.5, shape=21)
> plot
> h_bin8_all <- subset(h_bin8_all, PC1 > -1)
> h_bin8_all <- subset(h_bin8_all, PC2 < 2)
> plot <- ggplot(h_bin8_all, aes(x=GC_content, y=Sequencing_depth, fill=bin, size=Contig_length)) +
geom_point(alpha=0.5, shape=21)
> plot
> temp <- temp[!(temp$Contig %in% h_bin8_all$Contig), ]
> plot <- ggplot(temp, aes(x=gran_ratio, y=Sequencing_depth, fill=bin, size=Contig_length)) + geom_point(alpha=0.5,
shape=21)
> plot + xlim(0,0.5) + ylim(0,100)
Warning message:
Removed 66539 rows containing missing values (geom_point).
> plot + xlim(0,5) + ylim(0,100)
Warning message:
Removed 876 rows containing missing values (geom_point).
> plot + xlim(0,1) + ylim(0,100)
Warning message:
```

```
Removed 37843 rows containing missing values (geom_point).
> plot + xlim(0.2,0.8) + ylim(0,75)
Warning message:
Removed 56481 rows containing missing values (geom_point).
> i_bin9 <- subset(temp, gran_ratio > 0.4 & gran_ratio < 1 & Sequencing_depth > 10)
> plot <- ggplot(i_bin9, aes(x=gran_ratio, y=isolation_bias, fill=bin, size=Contig_length)) + geom_point(alpha=0.5,
shape=21)
> plot
> i_bin9 <- subset(i_bin9, isolation_bias < 0.4 & gran_ratio < 0.85)
> plot <- ggplot(i_bin9, aes(x=gran_ratio, y=isolation_bias, fill=bin, size=Contig_length)) + geom_point(alpha=0.5,
shape=21)
> plot
> plot <- ggplot(i_bin9, aes(x=GC_content, y=Sequencing_depth, fill=bin, size=Contig_length)) +
geom_point(alpha=0.5, shape=21)
> plot
> i_bin9 <- subset(i_bin9, isolation_bias < 0.35 & gran_ratio < 0.75)
> i_bin9 <- subset(i_bin9, isolation_bias > 0.1)
> plot <- ggplot(i_bin9, aes(x=gran_ratio, y=isolation_bias, fill=bin, size=Contig_length)) + geom_point(alpha=0.5,
shape=21)
> plot
> rda <- rda(i_bin9[,13:268], data=i_bin9[,1:12], scale=T)
> X_scores <- scores(rda, choices = 1:5)$sites
> i_bin9_all <- cbind(i_bin9, X_scores)
> pairs(i_bin9_all[,269:273], upper.panel=NULL)
> plot <- ggplot(i_bin9_all, aes(x=PC1, y=PC2, fill=bin, size=Contig_length)) + geom_point(alpha=0.5, shape=21)
> plot
> i_bin9_all <- subset(i_bin9_all, PC1 < 0.2 & PC2 > -0.5)
> plot <- ggplot(i_bin9_all, aes(x=PC1, y=PC2, fill=bin, size=Contig_length)) + geom_point(alpha=0.5, shape=21)
> plot
> plot <- ggplot(i_bin9_all, aes(x=gran_ratio, y=isolation_bias, fill=bin, size=Contig_length)) +
geom_point(alpha=0.5, shape=21)
> plot
> plot <- ggplot(i_bin9_all, aes(x=GC_content, y=Sequencing_depth, fill=bin, size=Contig_length)) +
geom_point(alpha=0.5, shape=21)
> plot
> i_bin9_all <- subset(i_bin9_all, GC_content > 0.55)
> i_bin9 <- i_bin9_all[-c(269:273)]
> rda <- rda(i_bin9[,13:268], data=i_bin9[,1:12], scale=T)
> X_scores <- scores(rda, choices = 1:5)$sites
> i_bin9_all <- cbind(i_bin9, X_scores)
> pairs(i_bin9_all[,269:273], upper.panel=NULL)
> plot <- ggplot(i_bin9_all, aes(x=PC1, y=PC2, fill=bin, size=Contig_length)) + geom_point(alpha=0.5, shape=21)
> plot
> i_bin9_all <- subset(i_bin9_all, PC2 > -1 & PC2 < 1 & PC1 < 0.5)
> plot <- ggplot(i_bin9_all, aes(x=PC1, y=PC3, fill=bin, size=Contig_length)) + geom_point(alpha=0.5, shape=21)
> plot
> i_bin9_all <- subset(i_bin9_all, PC3 < 0.5)
> plot <- ggplot(i_bin9_all, aes(x=gran_ratio, y=isolation_bias, fill=bin, size=Contig_length)) +
geom_point(alpha=0.5, shape=21)
> plot
> plot <- ggplot(i_bin9_all, aes(x=GC_content, y=Sequencing_depth, fill=bin, size=Contig_length)) +
geom_point(alpha=0.5, shape=21)
> plot
> plot + ylim(0,50)
```

```
Warning message:
Removed 6 rows containing missing values (geom_point).
> i_bin9_all <- subset(i_bin9_all, Sequencing_depth > 20 | GC_content > 0.624 )
> plot <- ggplot(i_bin9_all, aes(x=gran_ratio, y=isolation_bias, fill=bin, size=Contig_length)) +
geom_point(alpha=0.5, shape=21)
> plot
> i_bin9 <- i_bin9_all[-c(269:273)]
> rda <- rda(i_bin9[,13:268], data=i_bin9[,1:12], scale=T)
> X_scores <- scores(rda, choices = 1:5)$sites
> i_bin9_all <- cbind(i_bin9, X_scores)
> pairs(i_bin9_all[,269:273], upper.panel=NULL)
> plot <- ggplot(i_bin9_all, aes(x=PC1, y=PC2, fill=bin, size=Contig_length)) + geom_point(alpha=0.5, shape=21)
> plot
> plot <- ggplot(i_bin9_all, aes(x=gran_ratio, y=PC2, fill=bin, size=Contig_length)) + geom_point(alpha=0.5,
shape=21)
> plot
> plot <- ggplot(i_bin9_all, aes(x=isolation_bias, y=PC2, fill=bin, size=Contig_length)) + geom_point(alpha=0.5,
shape=21)
> plot
> plot <- ggplot(i_bin9_all, aes(x=isolation_bias, y=PC1, fill=bin, size=Contig_length)) + geom_point(alpha=0.5,
shape=21)
> plot
> plot <- ggplot(i_bin9_all, aes(x=gran_ratio, y=PC1, fill=bin, size=Contig_length)) + geom_point(alpha=0.5,
shape=21)
> plot
> plot <- ggplot(i_bin9_all, aes(x=GC_content, y=PC1, fill=bin, size=Contig_length)) + geom_point(alpha=0.5,
shape=21)
> plot
> plot <- ggplot(i_bin9_all, aes(x=GC_content, y=PC2, fill=bin, size=Contig_length)) + geom_point(alpha=0.5,
shape=21)
> plot
> plot <- ggplot(i_bin9_all, aes(x=Sequencing_depth, y=PC1, fill=bin, size=Contig_length)) + geom_point(alpha=0.5,
shape=21)
> plot
> plot <- ggplot(i_bin9_all, aes(x=Sequencing_depth, y=PC2, fill=bin, size=Contig_length)) + geom_point(alpha=0.5,
shape=21)
> plot
> i_bin9_all <- subset(i_bin9_all, PC2 < 1.5)
> temp <- temp[!(temp$Contig %in% i_bin9_all$Contig), ]
> plot <- ggplot(temp, aes(x=gran_ratio, y=Sequencing_depth, fill=bin, size=Contig_length)) + geom_point(alpha=0.5,
shape=21)
> plot
> plot + xlim(0.2,0.8) + ylim(0,75)
Warning message:
Removed 56480 rows containing missing values (geom_point).
> j_bin10 <- subset(temp, gran_ratio > 0.4 & gran_ratio < 0.8 & Sequencing_depth > 15)
> plot <- ggplot(j_bin10, aes(x=gran_ratio, y=isolation_bias, fill=bin, size=Contig_length)) + geom_point(alpha=0.5,
shape=21)
> plot
> j_bin10 <- subset(j_bin10, gran_ratio > 0.45 & gran_ratio < 0.7)
> j_bin10 <- subset(j_bin10, isolation_bias > 0.25 & isolation_bias < 0.7)
> plot <- ggplot(j_bin10, aes(x=gran_ratio, y=isolation_bias, fill=bin, size=Contig_length)) + geom_point(alpha=0.5,
shape=21)
> plot
```

```
> rda <- rda(j_bin10[,13:268], data=j_bin10[,1:12], scale=T)
> X_scores <- scores(rda, choices = 1:5)$sites
> j_bin10_all <- cbind(j_bin10, X_scores)
> pairs(j_bin10_all[,269:273], upper.panel=NULL)
> plot <- ggplot(j_bin10_all, aes(x=PC1, y=PC2, fill=bin, size=Contig_length)) + geom_point(alpha=0.5, shape=21)
> plot
> j_bin10_all <- subset(j_bin10_all, PC1 < 0 & PC2 > -1)
> plot <- ggplot(j_bin10_all, aes(x=PC1, y=PC2, fill=bin, size=Contig_length)) + geom_point(alpha=0.5, shape=21)
> plot
> j_bin10_all <- subset(j_bin10_all, PC1 < 0.75 & PC2 > -0.5 & PC2 < 2)
> plot <- ggplot(j_bin10_all, aes(x=PC1, y=PC2, fill=bin, size=Contig_length)) + geom_point(alpha=0.5, shape=21)
> plot
> plot <- ggplot(j_bin10_all, aes(x=PC1, y=PC3, fill=bin, size=Contig_length)) + geom_point(alpha=0.5, shape=21)
> plot
> j_bin10_all <- subset(j_bin10_all, PC1 < -0.25 & PC3 > -0.5)
> plot <- ggplot(j_bin10_all, aes(x=gran_ratio, y=isolation_bias, fill=bin, size=Contig_length)) +
geom_point(alpha=0.5, shape=21)
> plot
> plot <- ggplot(j_bin10_all, aes(x=GC_content, y=Sequencing_depth, fill=bin, size=Contig_length)) +
geom_point(alpha=0.5, shape=21)
> plot
> plot <- ggplot(j_bin10_all, aes(x=PC1, y=PC3, fill=bin, size=Contig_length)) + geom_point(alpha=0.5, shape=21)
> plot
> plot <- ggplot(j_bin10_all, aes(x=PC1, y=PC2, fill=bin, size=Contig_length)) + geom_point(alpha=0.5, shape=21)
> plot
> temp <- temp[!(temp$Contig %in% j_bin10_all$Contig), ]
> plot <- ggplot(temp, aes(x=gran_ratio, y=Sequencing_depth, fill=bin, size=Contig_length)) + geom_point(alpha=0.5,
shape=21)
> plot + xlim(0.2,1) + ylim(0,75)
Warning message:
Removed 46117 rows containing missing values (geom_point).
> k_bin11 <- subset(temp, gran_ratio < 0.6 & Sequencing_depth > 5)
> k_bin11 <- subset(k_bin11, gran_ratio > 0.2)
> k_bin11 <- subset(k_bin11, isolation_bias < 0.8)
> k_bin11 <- subset(k_bin11, isolation_bias > 0.2)
> plot <- ggplot(k_bin11, aes(x=gran_ratio, y=isolation_bias, fill=bin, size=Contig_length)) + geom_point(alpha=0.5,
shape=21)
> plot
> k_bin11 <- subset(k_bin11, isolation_bias > 0.3)
> plot <- ggplot(k_bin11, aes(x=gran_ratio, y=isolation_bias, fill=bin, size=Contig_length)) + geom_point(alpha=0.5,
shape=21)
> plot
> k_bin11 <- subset(k_bin11, gran_ratio < 0.55)
> k_bin11 <- subset(k_bin11, isolation_bias > 0.35)
> rda <- rda(k_bin11[,13:268], data=k_bin11[,1:12], scale=T)
> X_scores <- scores(rda, choices = 1:5)$sites
> k_bin11_all <- cbind(k_bin11, X_scores)
> pairs(k_bin11_all[,269:273], upper.panel=NULL)
> plot <- ggplot(k_bin11_all, aes(x=PC1, y=PC2, fill=bin, size=Contig_length)) + geom_point(alpha=0.5, shape=21)
> plot
> plot <- ggplot(k_bin11_all, aes(x=PC1, y=PC4, fill=bin, size=Contig_length)) + geom_point(alpha=0.5, shape=21)
> plot
> k_bin11_all <- subset(k_bin11_all, PC1 < 0)
> k_bin11_all <- subset(k_bin11_all, PC4 < 0.5)
```

```
> plot <- ggplot(k_bin11_all, aes(x=PC1, y=PC2, fill=bin, size=Contig_length)) + geom_point(alpha=0.5, shape=21)
> plot
> plot <- ggplot(k_bin11_all, aes(x=gran_ratio, y=isolation_bias, fill=bin, size=Contig_length)) +
geom_point(alpha=0.5, shape=21)
> plot
> k_bin11_all <- subset(k_bin11_all, isolation_bias < 0.6)
> k_bin11_all <- subset(k_bin11_all, gran_ratio > 0.3)
> plot <- ggplot(k_bin11_all, aes(x=gran_ratio, y=isolation_bias, fill=bin, size=Contig_length)) +
geom_point(alpha=0.5, shape=21)
> plot
> k_bin11 <- k_bin11_all[-c(269:273)]
> rda <- rda(k_bin11[,13:268], data=k_bin11[,1:12], scale=T)
> X_scores <- scores(rda, choices = 1:5)$sites
> k_bin11_all <- cbind(k_bin11, X_scores)
> pairs(k_bin11_all[,269:273], upper.panel=NULL)
> plot <- ggplot(k_bin11_all, aes(x=PC1, y=PC3, fill=bin, size=Contig_length)) + geom_point(alpha=0.5, shape=21)
> plot
> k_bin11_all <- subset(k_bin11_all, PC1 < 0 & PC3 < 0)
> plot <- ggplot(k_bin11_all, aes(x=gran_ratio, y=isolation_bias, fill=bin, size=Contig_length)) +
geom_point(alpha=0.5, shape=21)
> plot
> temp <- temp[!(temp$Contig %in% k_bin11_all$Contig), ]
> plot <- ggplot(temp, aes(x=gran_ratio, y=Sequencing_depth, fill=bin, size=Contig_length)) + geom_point(alpha=0.5,
shape=21)
> plot
> plot + xlim(0,5) + ylim(0,75)
Warning message:
Removed 918 rows containing missing values (geom_point).
> plot + xlim(1,2) + ylim(0,75)
Warning message:
Removed 61781 rows containing missing values (geom_point).
> l_bin12 <- subset(temp, gran_ratio > 1 & gran_ratio < 1.5 & Sequencing_depth > 5)
> plot <- ggplot(l_bin12, aes(x=gran_ratio, y=isolation_bias, fill=bin, size=Contig_length)) + geom_point(alpha=0.5,
shape=21)
> plot
> l_bin12 <- subset(l_bin12, isolation_bias < 0.5)
> plot <- ggplot(l_bin12, aes(x=gran_ratio, y=isolation_bias, fill=bin, size=Contig_length)) + geom_point(alpha=0.5,
shape=21)
> plot
> l_bin12 <- subset(l_bin12, isolation_bias < 0.4)
> l_bin12 <- subset(l_bin12, isolation_bias > 0.05)
> l_bin12 <- subset(l_bin12, gran_ratio > 1.05)
> l_bin12 <- subset(l_bin12, gran_ratio < 1.45)
> plot <- ggplot(l_bin12, aes(x=gran_ratio, y=isolation_bias, fill=bin, size=Contig_length)) + geom_point(alpha=0.5,
shape=21)
> plot
> rda <- rda(l_bin12[,13:268], data=l_bin12[,1:12], scale=T)
> X_scores <- scores(rda, choices = 1:5)$sites
> l_bin12_all <- cbind(l_bin12, X_scores)
> pairs(l_bin12_all[,269:273], upper.panel=NULL)
> plot <- ggplot(l_bin12_all, aes(x=PC1, y=PC2, fill=bin, size=Contig_length)) + geom_point(alpha=0.5, shape=21)
> plot
> l_bin12_all <- subset(l_bin12_all, PC1 > -0.2 & PC1 < 0.6)
> l_bin12_all <- subset(l_bin12_all, PC2 > -0.5 & PC2 < 0.6)
```

```
> plot <- ggplot(l_bin12_all, aes(x=PC1, y=PC2, fill=bin, size=Contig_length)) + geom_point(alpha=0.5, shape=21)
> plot
> l_bin12_all <- subset(l_bin12_all, PC2 > -0.2 & PC2 < 0.5)
> l_bin12_all <- subset(l_bin12_all, PC1 > -0.1 & PC1 < 0.5)
> l_bin12 <- l_bin12_all[-c(269:273)]
> rda <- rda(l_bin12[,13:268], data=l_bin12[,1:12], scale=T)
> X_scores <- scores(rda, choices = 1:5)$sites
> l_bin12_all <- cbind(l_bin12, X_scores)
> pairs(l_bin12_all[,269:273], upper.panel=NULL)
> plot <- ggplot(l_bin12_all, aes(x=PC1, y=PC2, fill=bin, size=Contig_length)) + geom_point(alpha=0.5, shape=21)
> plot
> l_bin12_all <- subset(l_bin12_all, PC1 > 0)
> l_bin12_all <- subset(l_bin12_all, PC2 > -1)
> plot <- ggplot(l_bin12_all, aes(x=gran_ratio, y=isolation_bias, fill=bin, size=Contig_length)) +
geom_point(alpha=0.5, shape=21)
> plot
> l_bin12_all <- subset(l_bin12_all, isolation_bias < 0.3)
> plot <- ggplot(l_bin12_all, aes(x=GC_content, y=Sequencing_depth, fill=bin, size=Contig_length)) +
geom_point(alpha=0.5, shape=21)
> plot
> l_bin12_all <- subset(l_bin12_all, Sequencing_depth > 12)
> plot <- ggplot(l_bin12_all, aes(x=GC_content, y=Sequencing_depth, fill=bin, size=Contig_length)) +
geom_point(alpha=0.5, shape=21)
> plot
> plot <- ggplot(l_bin12_all, aes(x=gran_ratio, y=isolation_bias, fill=bin, size=Contig_length)) +
geom_point(alpha=0.5, shape=21)
> plot
> l_bin12_all <- subset(l_bin12_all, GC_content > 0.5)
> plot <- ggplot(l_bin12_all, aes(x=gran_ratio, y=isolation_bias, fill=bin, size=Contig_length)) +
geom_point(alpha=0.5, shape=21)
> plot
> l_bin12 <- l_bin12_all[-c(269:273)]
> rda <- rda(l_bin12[,13:268], data=l_bin12[,1:12], scale=T)
> X_scores <- scores(rda, choices = 1:5)$sites
> l_bin12_all <- cbind(l_bin12, X_scores)
> pairs(l_bin12_all[,269:273], upper.panel=NULL)
> plot <- ggplot(l_bin12_all, aes(x=PC1, y=PC2, fill=bin, size=Contig_length)) + geom_point(alpha=0.5, shape=21)
> plot
> l_bin12_all <- subset(l_bin12_all, PC1 > -2)
> temp <- temp[!(temp$Contig %in% l_bin12_all$Contig), ]
> plot <- ggplot(temp, aes(x=gran_ratio, y=Sequencing_depth, fill=bin, size=Contig_length)) + geom_point(alpha=0.5,
shape=21)
> plot + xlim(1,2) + ylim(0,75)
Warning message:
Removed 61781 rows containing missing values (geom_point).
> m_bin13 <- subset(temp, gran_ratio > 1.3 & gran_ratio < 2 & Sequencing_depth > 10)
> m_bin13 <- subset(m_bin13, isolation_bias < 0.5)
> m_bin13 <- subset(m_bin13, isolation_bias > 0.1)
> m_bin13 <- subset(m_bin13, gran_ratio < 1.8)
> rda <- rda(m_bin13[,13:268], data=m_bin13[,1:12], scale=T)
> X_scores <- scores(rda, choices = 1:5)$sites
> m_bin13_all <- cbind(m_bin13, X_scores)
> pairs(m_bin13_all[,269:273], upper.panel=NULL)
> plot <- ggplot(m_bin13_all, aes(x=PC1, y=PC2, fill=bin, size=Contig_length)) + geom_point(alpha=0.5, shape=21)
```

```
> plot
> m_bin13_all <- subset(m_bin13_all, PC2 < 0.8 & PC1 > -1 & PC1 < 0.5 & PC2 > -1)
> plot <- ggplot(m_bin13_all, aes(x=PC1, y=PC2, fill=bin, size=Contig_length)) + geom_point(alpha=0.5, shape=21)
> plot
> m_bin13_all <- subset(m_bin13_all, PC2 < 0.8 & PC1 > -1 & PC1 < 0.5 & PC2 > -0.5)
> m_bin13_all <- subset(m_bin13_all, PC2 < 0.8 & PC1 > -0.75 & PC1 < 0.25 & PC2 > -0.5)
> plot <- ggplot(m_bin13_all, aes(x=PC1, y=PC2, fill=bin, size=Contig_length)) + geom_point(alpha=0.5, shape=21)
> plot
> plot <- ggplot(m_bin13_all, aes(x=gran_ratio, y=isolation_bias, fill=bin, size=Contig_length)) +
geom_point(alpha=0.5, shape=21)
> plot
> m_bin13_all <- subset(m_bin13_all, isolation_bias < 0.3)
> m_bin13_all <- subset(m_bin13_all, isolation_bias > 0.15)
> plot <- ggplot(m_bin13_all, aes(x=gran_ratio, y=isolation_bias, fill=bin, size=Contig_length)) +
geom_point(alpha=0.5, shape=21)
> plot
> m_bin13 <- m_bin13_all[-c(269:273)]
> rda <- rda(m_bin13[,13:268], data=m_bin13[,1:12], scale=T)
> X_scores <- scores(rda, choices = 1:5)$sites
> m_bin13_all <- cbind(m_bin13, X_scores)
> pairs(m_bin13_all[,269:273], upper.panel=NULL)
> plot <- ggplot(m_bin13_all, aes(x=PC1, y=PC2, fill=bin, size=Contig_length)) + geom_point(alpha=0.5, shape=21)
> plot
> plot <- ggplot(m_bin13_all, aes(x=PC1, y=PC3, fill=bin, size=Contig_length)) + geom_point(alpha=0.5, shape=21)
> plot
> m_bin13_all <- subset(m_bin13_all, PC3 < -1)
> plot <- ggplot(m_bin13_all, aes(x=gran_ratio, y=isolation_bias, fill=bin, size=Contig_length)) +
geom_point(alpha=0.5, shape=21)
> plot
> m_bin13_all <- subset(m_bin13_all, isolation_bias > 0.17)
> m_bin13_all <- subset(m_bin13_all, isolation_bias < 0.27)
> plot <- ggplot(m_bin13_all, aes(x=GC_content, y=Sequencing_depth, fill=bin, size=Contig_length)) +
geom_point(alpha=0.5, shape=21)
> plot
> temp <- temp[!(temp$Contig %in% m_bin13_all$Contig), ]
> plot <- ggplot(temp, aes(x=gran_ratio, y=Sequencing_depth, fill=bin, size=Contig_length)) + geom_point(alpha=0.5,
shape=21)
> plot + xlim(1,2) + ylim(0,75)
Warning message:
Removed 61781 rows containing missing values (geom_point).
> n_bin14 <- subset(temp, gran_ratio > 1.3 & gran_ratio < 2.5 & Sequencing_depth > 10)
> plot <- ggplot(n_bin14, aes(x=gran_ratio, y=isolation_bias, fill=bin, size=Contig_length)) + geom_point(alpha=0.5,
shape=21)
> plot
> n_bin14 <- subset(n_bin14, isolation_bias < 0.5)
> plot <- ggplot(n_bin14, aes(x=gran_ratio, y=isolation_bias, fill=bin, size=Contig_length)) + geom_point(alpha=0.5,
shape=21)
> plot
> n_bin14 <- subset(n_bin14, isolation_bias < 0.3)
> n_bin14 <- subset(n_bin14, gran_ratio < 2.2)
> plot <- ggplot(n_bin14, aes(x=gran_ratio, y=isolation_bias, fill=bin, size=Contig_length)) + geom_point(alpha=0.5,
shape=21)
> plot
> rda <- rda(n_bin14[,13:268], data=n_bin14[,1:12], scale=T)
```

```
> X_scores <- scores(rda, choices = 1:5)$sites
> n_bin14_all <- cbind(n_bin14, X_scores)
> pairs(n_bin14_all[,269:273], upper.panel=NULL)
> plot <- ggplot(n_bin14_all, aes(x=PC1, y=PC2, fill=bin, size=Contig_length)) + geom_point(alpha=0.5, shape=21)
> plot
> n_bin14_all <- subset(n_bin14_all, PC1 > -1 & PC1 < 0.5)
> n_bin14_all <- subset(n_bin14_all, PC2 > -0.5 & PC2 < 1)
> plot <- ggplot(n_bin14_all, aes(x=PC1, y=PC2, fill=bin, size=Contig_length)) + geom_point(alpha=0.5, shape=21)
> plot
> n_bin14_all <- subset(n_bin14_all, PC2 > 0 & PC2 < 0.7)
> n_bin14_all <- subset(n_bin14_all, PC1 > -0.7 & PC1 < 0)
> plot <- ggplot(n_bin14, aes(x=gran_ratio, y=isolation_bias, fill=bin, size=Contig_length)) + geom_point(alpha=0.5,
shape=21)
> plot
> plot <- ggplot(n_bin14_all, aes(x=gran_ratio, y=isolation_bias, fill=bin, size=Contig_length)) +
geom_point(alpha=0.5, shape=21)
> plot
> n_bin14_all <- subset(n_bin14_all, isolation_bias < 0.2)
> n_bin14_all <- subset(n_bin14_all, gran_ratio > 1.5)
> plot <- ggplot(n_bin14_all, aes(x=gran_ratio, y=isolation_bias, fill=bin, size=Contig_length)) +
geom_point(alpha=0.5, shape=21)
> plot
> n_bin14 <- n_bin14_all[-c(269:273)]
> rda <- rda(n_bin14[,13:268], data=n_bin14[,1:12], scale=T)
> X_scores <- scores(rda, choices = 1:5)$sites
> n_bin14_all <- cbind(n_bin14, X_scores)
> pairs(n_bin14_all[,269:273], upper.panel=NULL)
> plot <- ggplot(n_bin14_all, aes(x=PC1, y=PC3, fill=bin, size=Contig_length)) + geom_point(alpha=0.5, shape=21)
> plot
> n_bin14_all <- subset(n_bin14_all, PC1 > 0.5 & PC3 > 0.1)
> plot <- ggplot(n_bin14_all, aes(x=PC1, y=PC3, fill=bin, size=Contig_length)) + geom_point(alpha=0.5, shape=21)
> plot
> plot <- ggplot(n_bin14_all, aes(x=gran_ratio, y=isolation_bias, fill=bin, size=Contig_length)) +
geom_point(alpha=0.5, shape=21)
> plot
> temp <- temp[!(temp$Contig %in% n_bin14_all$Contig), ]
> plot <- ggplot(temp, aes(x=gran_ratio, y=Sequencing_depth, fill=bin, size=Contig_length)) + geom_point(alpha=0.5,
shape=21)
> plot + xlim(0,3) + ylim(0,75)
Warning message:
Removed 3011 rows containing missing values (geom_point).
> plot + xlim(0,1) + ylim(0,30)
Warning message:
Removed 37932 rows containing missing values (geom_point).
> o_bin15 <- subset(temp, gran_ratio > 0.3 & gran_ratio < 1 & Sequencing_depth > 5)
> plot <- ggplot(o_bin15, aes(x=gran_ratio, y=isolation_bias, fill=bin, size=Contig_length)) + geom_point(alpha=0.5,
shape=21)
> plot
> o_bin15 <- subset(o_bin15, isolation_bias < 0.5)
> plot <- ggplot(o_bin15, aes(x=gran_ratio, y=isolation_bias, fill=bin, size=Contig_length)) + geom_point(alpha=0.5,
shape=21)
> plot
> o_bin15 <- subset(o_bin15, isolation_bias < 0.3)
> o_bin15 <- subset(o_bin15, gran_ratio > 0.4 & gran_ratio < 0.8)
```

```
> rda <- rda(o_bin15[,13:268], data=o_bin15[,1:12], scale=T)
> X_scores <- scores(rda, choices = 1:5)$sites
> o_bin15_all <- cbind(o_bin15,X_scores)
> pairs(o_bin15_all[,269:273], upper.panel=NULL)
> plot <- ggplot(o_bin15_all, aes(x=PC1, y=PC2, fill=bin, size=Contig_length)) + geom_point(alpha=0.5, shape=21)
> plot
> plot <- ggplot(o_bin15_all, aes(x=PC1, y=PC3, fill=bin, size=Contig_length)) + geom_point(alpha=0.5, shape=21)
> plot
> o_bin15_all <- subset(o_bin15_all, PC1 > 0.2 & PC3 < 0.5)
> plot <- ggplot(o_bin15_all, aes(x=PC1, y=PC3, fill=bin, size=Contig_length)) + geom_point(alpha=0.5, shape=21)
> plot
> o_bin15_all <- subset(o_bin15_all, PC3 > -0.5 & PC3 < 0.2)
> o_bin15_all <- subset(o_bin15_all, PC1 > 0.3)
> plot <- ggplot(o_bin15_all, aes(x=PC1, y=PC3, fill=bin, size=Contig_length)) + geom_point(alpha=0.5, shape=21)
> plot
> o_bin15_all <- subset(o_bin15_all, PC3 < 0)
> plot <- ggplot(o_bin15_all, aes(x=gran_ratio, y=isolation_bias, fill=bin, size=Contig_length)) +
geom_point(alpha=0.5, shape=21)
> plot
> o_bin15_all <- subset(o_bin15_all, isolation_bias < 0.2)
> o_bin15_all <- subset(o_bin15_all, gran_ratio > 0.5)
> o_bin15 <- o_bin15_all[-c(269:273)]
> rda <- rda(o_bin15[,13:268], data=o_bin15[,1:12], scale=T)
> X_scores <- scores(rda, choices = 1:5)$sites
> o_bin15_all <- cbind(o_bin15,X_scores)
> pairs(o_bin15_all[,269:273], upper.panel=NULL)
> plot <- ggplot(o_bin15_all, aes(x=PC1, y=PC3, fill=bin, size=Contig_length)) + geom_point(alpha=0.5, shape=21)
> plot
> o_bin15_all <- subset(o_bin15_all, PC3 < 0.5)
> o_bin15_all <- subset(o_bin15_all, PC1 > -1)
> plot <- ggplot(o_bin15_all, aes(x=PC1, y=PC3, fill=bin, size=Contig_length)) + geom_point(alpha=0.5, shape=21)
> plot
> plot <- ggplot(o_bin15_all, aes(x=gran_ratio, y=isolation_bias, fill=bin, size=Contig_length)) +
geom_point(alpha=0.5, shape=21)
> plot
> plot <- ggplot(o_bin15_all, aes(x=PC1, y=PC2, fill=bin, size=Contig_length)) + geom_point(alpha=0.5, shape=21)
> plot
> o_bin15_all <- subset(o_bin15_all, PC2 > -1)
> o_bin15_all <- subset(o_bin15_all, PC1 < 1)
> plot <- ggplot(o_bin15_all, aes(x=gran_ratio, y=isolation_bias, fill=bin, size=Contig_length)) +
geom_point(alpha=0.5, shape=21)
> plot
> plot <- ggplot(o_bin15_all, aes(x=GC_content, y=Sequencing_depth, fill=bin, size=Contig_length)) +
geom_point(alpha=0.5, shape=21)
> plot
> plot + ylim(0,50)
Warning message:
Removed 1 rows containing missing values (geom_point).
> o_bin15_all <- subset(o_bin15_all, Sequencing_depth > 10)
> temp <- temp[!(temp$Contig %in% o_bin15_all$Contig), ]
> plot <- ggplot(temp, aes(x=gran_ratio, y=Sequencing_depth, fill=bin, size=Contig_length)) + geom_point(alpha=0.5,
shape=21)
> plot + xlim(0,3) + ylim(0,50)
Warning message:
```

```
Removed 3151 rows containing missing values (geom_point).
> p_bin16 <- subset(temp, gran_ratio > 1 & gran_ratio < 2 & Sequencing_depth > 10)
> plot <- ggplot(temp, aes(x=gran_ratio, y=isolation_bias, fill=bin, size=Contig_length)) + geom_point(alpha=0.5, shape=21)
> plot
> plot <- ggplot(p_bin16, aes(x=gran_ratio, y=isolation_bias, fill=bin, size=Contig_length)) + geom_point(alpha=0.5, shape=21)
> plot
> p_bin16 <- subset(p_bin16, isolation_bias < 0.4)
> plot <- ggplot(p_bin16, aes(x=gran_ratio, y=isolation_bias, fill=bin, size=Contig_length)) + geom_point(alpha=0.5, shape=21)
> plot
> rda <- rda(p_bin16[,13:268], data=p_bin16[,1:12], scale=T)
> X_scores <- scores(rda, choices = 1:5)$sites
> p_bin16_all <- cbind(p_bin16,X_scores)
> pairs(p_bin16_all[,269:273], upper.panel=NULL)
> plot <- ggplot(p_bin16_all, aes(x=PC1, y=PC2, fill=bin, size=Contig_length)) + geom_point(alpha=0.5, shape=21)
> plot
> p_bin16_all <-subset(p_bin16_all, PC2 > 0 & PC1 > -0.5)
> plot <- ggplot(p_bin16_all, aes(x=PC1, y=PC2, fill=bin, size=Contig_length)) + geom_point(alpha=0.5, shape=21)
> plot
> plot <- ggplot(p_bin16_all, aes(x=PC4, y=PC2, fill=bin, size=Contig_length)) + geom_point(alpha=0.5, shape=21)
> plot
> plot <- ggplot(p_bin16_all, aes(x=gran_ratio, y=isolation_bias, fill=bin, size=Contig_length)) + geom_point(alpha=0.5, shape=21)
> plot
> p_bin16_all <-subset(p_bin16_all,gran_ratio > 1.2)
> p_bin16_all <-subset(p_bin16_all, isolation_bias < 0.25)
> p_bin16 <- p_bin16_all[-c(269:273)]
> rda <- rda(p_bin16[,13:268], data=p_bin16[,1:12], scale=T)
> X_scores <- scores(rda, choices = 1:5)$sites
> p_bin16_all <- cbind(p_bin16,X_scores)
> pairs(p_bin16_all[,269:273], upper.panel=NULL)
> plot <- ggplot(p_bin16_all, aes(x=PC1, y=PC2, fill=bin, size=Contig_length)) + geom_point(alpha=0.5, shape=21)
> plot
> p_bin16_all <-subset(p_bin16_all, PC2 > -0.5)
> plot <- ggplot(p_bin16_all, aes(x=PC3, y=PC2, fill=bin, size=Contig_length)) + geom_point(alpha=0.5, shape=21)
> plot
> plot <- ggplot(p_bin16_all, aes(x=PC3, y=PC1, fill=bin, size=Contig_length)) + geom_point(alpha=0.5, shape=21)
> plot
> p_bin16_all <-subset(p_bin16_all, PC1 > 0 | PC3 < -1)
> plot <- ggplot(p_bin16_all, aes(x=PC3, y=PC1, fill=bin, size=Contig_length)) + geom_point(alpha=0.5, shape=21)
> plot
> plot <- ggplot(p_bin16_all, aes(x=gran_ratio, y=isolation_bias, fill=bin, size=Contig_length)) + geom_point(alpha=0.5, shape=21)
> plot
> plot <- ggplot(p_bin16_all, aes(x=GC_content, y=Sequencing_depth, fill=bin, size=Contig_length)) + geom_point(alpha=0.5, shape=21)
> plot
> plot + ylim(0,50)
Warning message:
Removed 2 rows containing missing values (geom_point).
> p_bin16_all <-subset(p_bin16_all, PC1 > 1 | PC3 < 0)
> plot <- ggplot(p_bin16_all, aes(x=PC3, y=PC1, fill=bin, size=Contig_length)) + geom_point(alpha=0.5, shape=21)
```

```
> plot
> p_bin16 <- p_bin16_all[-c(269:273)]
> rda <- rda(p_bin16[,13:268], data=p_bin16[,1:12], scale=T)
> X_scores <- scores(rda, choices = 1:5)$sites
> p_bin16_all <- cbind(p_bin16,X_scores)
> pairs(p_bin16_all[,269:273], upper.panel=NULL)
> plot <- ggplot(p_bin16_all, aes(x=PC1, y=PC2, fill=bin, size=Contig_length)) + geom_point(alpha=0.5, shape=21)
> plot
> p_bin16_all <- subset(p_bin16_all, PC1 > -1.5 & PC2 < 2)
> plot <- ggplot(p_bin16_all, aes(x=PC1, y=PC2, fill=bin, size=Contig_length)) + geom_point(alpha=0.5, shape=21)
> plot
> plot <- ggplot(p_bin16_all, aes(x=gran_ratio, y=isolation_bias, fill=bin, size=Contig_length)) +
geom_point(alpha=0.5, shape=21)
> plot
> plot <- ggplot(p_bin16_all, aes(x=GC_content, y=Sequencing_depth, fill=bin, size=Contig_length)) +
geom_point(alpha=0.5, shape=21)
> plot
> temp <- temp[!(temp$Contig %in% p_bin16_all$Contig), ]
> q_bin17 <- subset(temp, gran_ratio > 1 & gran_ratio < 2 & Sequencing_depth > 10)
> plot <- ggplot(temp, aes(x=gran_ratio, y=Sequencing_depth, fill=bin, size=Contig_length)) + geom_point(alpha=0.5,
shape=21)
> plot <- ggplot(q_bin17, aes(x=gran_ratio, y=Sequencing_depth, fill=bin, size=Contig_length)) +
geom_point(alpha=0.5, shape=21)
> plot
> plot + ylim(0,50)
Warning message:
Removed 81 rows containing missing values (geom_point).
> q_bin17 <- subset(q_bin17, gran_ratio > 1.2)
> plot <- ggplot(q_bin17, aes(x=gran_ratio, y=isolation_bias, fill=bin, size=Contig_length)) + geom_point(alpha=0.5,
shape=21)
> plot
> q_bin17 <- subset(q_bin17, isolation_bias < 0.4)
> plot <- ggplot(q_bin17, aes(x=gran_ratio, y=isolation_bias, fill=bin, size=Contig_length)) + geom_point(alpha=0.5,
shape=21)
> plot
> rda <- rda(q_bin17[,13:268], data=q_bin17[,1:12], scale=T)
> X_scores <- scores(rda, choices = 1:5)$sites
> q_bin17_all <- cbind(q_bin17,X_scores)
> pairs(q_bin17_all[,269:273], upper.panel=NULL)
> plot <- ggplot(q_bin17_all, aes(x=PC1, y=PC2, fill=bin, size=Contig_length)) + geom_point(alpha=0.5, shape=21)
> plot
> q_bin17_all <- subset(q_bin17_all, PC2 > 0.5)
> plot <- ggplot(q_bin17_all, aes(x=PC3, y=PC2, fill=bin, size=Contig_length)) + geom_point(alpha=0.5, shape=21)
> plot
> q_bin17_all <- subset(q_bin17_all, PC2 > 0.8 | PC3 > 0)
> plot <- ggplot(q_bin17_all, aes(x=PC3, y=PC2, fill=bin, size=Contig_length)) + geom_point(alpha=0.5, shape=21)
> plot
> q_bin17_all <- subset(q_bin17_all, PC3 > -0.5)
> plot <- ggplot(q_bin17_all, aes(x=PC3, y=PC2, fill=bin, size=Contig_length)) + geom_point(alpha=0.5, shape=21)
> plot
> q_bin17_all <- subset(q_bin17_all, PC2 > 0.7)
> q_bin17_all <- subset(q_bin17_all, PC3 < 1)
> plot <- ggplot(q_bin17_all, aes(x=gran_ratio, y=isolation_bias, fill=bin, size=Contig_length)) +
geom_point(alpha=0.5, shape=21)
```

```
> plot
> q_bin17_all <- subset(q_bin17_all, isolation_bias < 0.25)
> temp <- temp[!(temp$Contig %in% q_bin17_all$Contig), ]
> plot <- ggplot(q_bin17_all, aes(x=GC_content, y=Sequencing_depth, fill=bin, size=Contig_length)) +
geom_point(alpha=0.5, shape=21)
> plot
> r_bin18 <- subset(temp, gran_ratio > 1 & gran_ratio < 2 & Sequencing_depth > 5)
> plot <- ggplot(r_bin18, aes(x=gran_ratio, y=isolation_bias, fill=bin, size=Contig_length)) + geom_point(alpha=0.5,
shape=21)
> plot
> r_bin18 <- subset(r_bin18, isolation_bias < 0.4)
> plot <- ggplot(r_bin18, aes(x=gran_ratio, y=isolation_bias, fill=bin, size=Contig_length)) + geom_point(alpha=0.5,
shape=21)
> plot
> r_bin18 <- subset(r_bin18, gran_ratio > 1.2)
> r_bin18 <- subset(r_bin18, isolation_bias > 0.02)
> r_bin18 <- subset(r_bin18, isolation_bias < 0.25)
> rda <- rda(r_bin18[,13:268], data=r_bin18[,1:12], scale=T)
> X_scores <- scores(rda, choices = 1:5)$sites
> r_bin18_all <- cbind(r_bin18, X_scores)
> pairs(r_bin18_all[,269:273], upper.panel=NULL)
> plot <- ggplot(r_bin18_all, aes(x=PC1, y=PC2, fill=bin, size=Contig_length)) + geom_point(alpha=0.5, shape=21)
> plot
> r_bin18_all <- subset(r_bin18_all, PC1 > 0.2)
> plot <- ggplot(r_bin18_all, aes(x=PC1, y=PC2, fill=bin, size=Contig_length)) + geom_point(alpha=0.5, shape=21)
> plot
> plot <- ggplot(r_bin18_all, aes(x=PC3, y=PC2, fill=bin, size=Contig_length)) + geom_point(alpha=0.5, shape=21)
> plot
> r_bin18_all <- subset(r_bin18_all, PC2 < 0.4)
> plot <- ggplot(r_bin18_all, aes(x=gran_ratio, y=isolation_bias, fill=bin, size=Contig_length)) +
geom_point(alpha=0.5, shape=21)
> plot
> r_bin18_all <- subset(r_bin18_all, isolation_bias < 0.2)
> r_bin18 <- r_bin18_all[-c(269:273)]
> rda <- rda(r_bin18[,13:268], data=r_bin18[,1:12], scale=T)
> X_scores <- scores(rda, choices = 1:5)$sites
> r_bin18_all <- cbind(r_bin18, X_scores)
> pairs(r_bin18_all[,269:273], upper.panel=NULL)
> plot <- ggplot(r_bin18_all, aes(x=PC1, y=PC2, fill=bin, size=Contig_length)) + geom_point(alpha=0.5, shape=21)
> plot
> plot <- ggplot(r_bin18_all, aes(x=PC1, y=PC3, fill=bin, size=Contig_length)) + geom_point(alpha=0.5, shape=21)
> plot
> plot <- ggplot(r_bin18_all, aes(x=PC2, y=PC3, fill=bin, size=Contig_length)) + geom_point(alpha=0.5, shape=21)
> plot
> r_bin18_all <- subset(r_bin18_all, PC2 > -0.1)
> r_bin18_all <- subset(r_bin18_all, PC3 > -0.5)
> plot <- ggplot(r_bin18_all, aes(x=PC1, y=PC2, fill=bin, size=Contig_length)) + geom_point(alpha=0.5, shape=21)
> plot
> plot <- ggplot(r_bin18_all, aes(x=gran_ratio, y=isolation_bias, fill=bin, size=Contig_length)) +
geom_point(alpha=0.5, shape=21)
> plot
> r_bin18_all <- subset(r_bin18_all, PC1 > -1)
> r_bin18_all <- subset(r_bin18_all, PC1 > 0 | PC2 > 0.75)
> plot <- ggplot(r_bin18_all, aes(x=PC1, y=PC2, fill=bin, size=Contig_length)) + geom_point(alpha=0.5, shape=21)
```

```
> plot
> r_bin18_all <- subset(r_bin18_all, PC1 < 1)
> r_bin18 <- r_bin18_all[-c(269:273)]
> rda <- rda(r_bin18[,13:268], data=r_bin18[,1:12], scale=T)
> X_scores <- scores(rda, choices = 1:5)$sites
> r_bin18_all <- cbind(r_bin18, X_scores)
> pairs(r_bin18_all[,269:273], upper.panel=NULL)
> plot <- ggplot(r_bin18_all, aes(x=PC1, y=PC2, fill=bin, size=Contig_length)) + geom_point(alpha=0.5, shape=21)
> plot
> s_bin19 <- subset(r_bin18_all, PC1 > 0 & PC2 < 0)
> r_bin18_all <- subset(r_bin18_all, PC1 < 0.2)
> plot <- ggplot(r_bin18_all, aes(x=isolation_bias, y=gran_ratio, fill=bin, size=Contig_length)) +
geom_point(alpha=0.5, shape=21)
> plot
> plot <- ggplot(r_bin18_all, aes(x=GC_content, y=Sequencing_depth, fill=bin, size=Contig_length)) +
geom_point(alpha=0.5, shape=21)
> plot
> r_bin18_all <- subset(r_bin18_all, Sequencing_depth > 8)
> plot <- ggplot(r_bin18_all, aes(x=GC_content, y=Sequencing_depth, fill=bin, size=Contig_length)) +
geom_point(alpha=0.5, shape=21)
> plot
> plot <- ggplot(r_bin18_all, aes(x=isolation_bias, y=gran_ratio, fill=bin, size=Contig_length)) +
geom_point(alpha=0.5, shape=21)
> plot
> temp <- temp[!(temp$Contig %in% r_bin18_all$Contig), ]
> plot <- ggplot(s_bin19, aes(x=GC_content, y=Sequencing_depth, fill=bin, size=Contig_length)) +
geom_point(alpha=0.5, shape=21)
> plot
> s_bin19_all <- s_bin19
> s_bin19_all <- subset(s_bin19_all, Sequencing_depth > 8)
> plot <- ggplot(s_bin19, aes(x=GC_content, y=Sequencing_depth, fill=bin, size=Contig_length)) +
geom_point(alpha=0.5, shape=21)
>
> plot
> plot <- ggplot(s_bin19_all, aes(x=GC_content, y=Sequencing_depth, fill=bin, size=Contig_length)) +
geom_point(alpha=0.5, shape=21)
> plot
> plot <- ggplot(s_bin19_all, aes(x=gran_ratio, y=isolation_bias, fill=bin, size=Contig_length)) +
geom_point(alpha=0.5, shape=21)
> plot
> s_bin19_all <- subset(s_bin19_all, GC_content > 0.36)
> temp <- temp[!(temp$Contig %in% s_bin19_all$Contig), ]
> plot <- ggplot(temp, aes(x=isolation_bias, y=Sequencing_depth, fill=bin, size=Contig_length)) +
geom_point(alpha=0.5, shape=21)
> plot
> plot + xlim(0,2)
Warning message:
Removed 154 rows containing missing values (geom_point).
> plot + xlim(0,2) + ylim(0,50)
Warning message:
Removed 391 rows containing missing values (geom_point).
> plot + xlim(0,1) + ylim(0,20)
Warning message:
Removed 3198 rows containing missing values (geom_point).
```

```
> t_bin20 <- subset(temp, isolation_bias > 0.25 & isolation_bias < 1 & Sequencing_depth > 5)
> plot <- ggplot(t_bin20, aes(x=isolation_bias, y=gran_ratio, fill=bin, size=Contig_length)) + geom_point(alpha=0.5, shape=21)
> plot
> t_bin20 <- subset(t_bin20, gran_ratio < 2)
> plot <- ggplot(t_bin20, aes(x=isolation_bias, y=gran_ratio, fill=bin, size=Contig_length)) + geom_point(alpha=0.5, shape=21)
> plot
> t_bin20 <- subset(t_bin20, gran_ratio < 1.5 & gran_ratio > 0.5)
> t_bin20 <- subset(t_bin20, isolation_bias < 0.8)
> t_bin20 <- subset(t_bin20, isolation_bias > 0.3)
> rda <- rda(t_bin20[,13:268], data=t_bin20[,1:12], scale=T)
> X_scores <- scores(rda, choices = 1:5)$sites
> t_bin20_all <- cbind(t_bin20, X_scores)
> pairs(t_bin20_all[,269:273], upper.panel=NULL)
> plot <- ggplot(t_bin20_all, aes(x=PC1, y=PC2, fill=bin, size=Contig_length)) + geom_point(alpha=0.5, shape=21)
> plot
> t_bin20_all <- subset(t_bin20_all, PC1 < 0)
> t_bin20_all <- subset(t_bin20_all, PC2 < 0.5)
> plot <- ggplot(t_bin20_all, aes(x=PC1, y=PC2, fill=bin, size=Contig_length)) + geom_point(alpha=0.5, shape=21)
> plot
> t_bin20_all <- subset(t_bin20_all, PC1 > -0.5)
> t_bin20_all <- subset(t_bin20_all, PC1 < -0.1)
> t_bin20 <- t_bin20_all[-c(269:273)]
> plot <- ggplot(t_bin20_all, aes(x=isolation_bias, y=gran_ratio, fill=bin, size=Contig_length)) + geom_point(alpha=0.5, shape=21)
> plot
> t_bin20_all <- subset(t_bin20_all, gran_ratio < 1.25)
> t_bin20_all <- subset(t_bin20_all, gran_ratio > 0.65)
> t_bin20_all <- subset(t_bin20_all, isolation_bias < 0.75)
> t_bin20_all <- subset(t_bin20_all, isolation_bias > 0.35)
> plot <- ggplot(t_bin20_all, aes(x=isolation_bias, y=gran_ratio, fill=bin, size=Contig_length)) + geom_point(alpha=0.5, shape=21)
> plot
> plot <- ggplot(t_bin20_all, aes(x=GC_content, y=Sequencing_depth, fill=bin, size=Contig_length)) + geom_point(alpha=0.5, shape=21)
> plot
> plot + ylim(0,20)
Warning message:
Removed 69 rows containing missing values (geom_point).
> t_bin20_all <- subset(t_bin20_all, Sequencing_depth > 7)
> t_bin20 <- t_bin20_all[-c(269:273)]
> rda <- rda(t_bin20[,13:268], data=t_bin20[,1:12], scale=T)
> X_scores <- scores(rda, choices = 1:5)$sites
> t_bin20_all <- cbind(t_bin20, X_scores)
> pairs(t_bin20_all[,269:273], upper.panel=NULL)
> plot <- ggplot(t_bin20_all, aes(x=PC1, y=PC2, fill=bin, size=Contig_length)) + geom_point(alpha=0.5, shape=21)
> plot
> t_bin20_all <- subset(t_bin20_all, PC1 > -0.5)
> plot <- ggplot(t_bin20_all, aes(x=PC1, y=PC2, fill=bin, size=Contig_length)) + geom_point(alpha=0.5, shape=21)
> plot
> plot <- ggplot(t_bin20_all, aes(x=isolation_bias, y=gran_ratio, fill=bin, size=Contig_length)) + geom_point(alpha=0.5, shape=21)
> plot
```

```
> t_bin20_all <- subset(t_bin20_all, gran_ratio > 0.7)
> t_bin20 <- t_bin20_all[-c(269:273)]
> rda <- rda(t_bin20[,13:268], data=t_bin20[,1:12], scale=T)
> X_scores <- scores(rda, choices = 1:5)$sites
> t_bin20_all <- cbind(t_bin20, X_scores)
> pairs(t_bin20_all[,269:273], upper.panel=NULL)
> plot <- ggplot(t_bin20_all, aes(x=PC1, y=PC2, fill=bin, size=Contig_length)) + geom_point(alpha=0.5, shape=21)
> plot
> plot <- ggplot(t_bin20_all, aes(x=PC1, y=PC3, fill=bin, size=Contig_length)) + geom_point(alpha=0.5, shape=21)
> plot
> t_bin20_all <- subset(t_bin20_all, PC1 < 1.1)
> plot <- ggplot(t_bin20_all, aes(x=PC1, y=PC3, fill=bin, size=Contig_length)) + geom_point(alpha=0.5, shape=21)
> plot
> t_bin20_all <- subset(t_bin20_all, PC3 > -2)
> t_bin20_all <- subset(t_bin20_all, PC3 < 2)
> plot <- ggplot(t_bin20_all, aes(x=isolation_bias, y=gran_ratio, fill=bin, size=Contig_length)) +
geom_point(alpha=0.5, shape=21)
> plot
> plot + ylim(0,100)
Warning message:
Removed 289 rows containing missing values (geom_point).
> temp <- temp[!(temp$Contig %in% t_bin20_all$Contig), ]
> plot <- ggplot(temp, aes(x=isolation_bias, y=Sequencing_depth, fill=bin, size=Contig_length)) +
geom_point(alpha=0.5, shape=21)
> plot
> plot + xlim(0,2) + ylim(0,100)
Warning message:
Removed 206 rows containing missing values (geom_point).
> plot + xlim(0,2) + ylim(0,10)
Warning message:
Removed 7165 rows containing missing values (geom_point).
> plot + xlim(0,0.5) + ylim(0,10)
Warning message:
Removed 15250 rows containing missing values (geom_point).
> plot + xlim(0,0.5) + ylim(0,50)
Warning message:
Removed 8730 rows containing missing values (geom_point).
> u_bin21 <- subset(temp, isolation_bias < 0.5 & Sequencing_depth > 5)
> u_bin21 <- subset(u_bin21, gran_ratio < 5)
> plot <- ggplot(u_bin21, aes(x=isolation_bias, y=gran_ratio, fill=bin, size=Contig_length)) + geom_point(alpha=0.5,
shape=21)
> plot
> u_bin21 <- subset(u_bin21, gran_ratio < 2)
> plot <- ggplot(u_bin21, aes(x=isolation_bias, y=gran_ratio, fill=bin, size=Contig_length)) + geom_point(alpha=0.5,
shape=21)
> plot
> u_bin21 <- subset(u_bin21, gran_ratio < 1.2)
> u_bin21 <- subset(u_bin21, isolation_bias > 0.01)
> plot <- ggplot(u_bin21, aes(x=isolation_bias, y=gran_ratio, fill=bin, size=Contig_length)) + geom_point(alpha=0.5,
shape=21)
> plot
> u_bin21 <- subset(u_bin21, gran_ratio > 0.3)
> rda <- rda(u_bin21[,13:268], data=u_bin21[,1:12], scale=T)
> X_scores <- scores(rda, choices = 1:5)$sites
```

```
> u_bin21_all <- cbind(u_bin21, X_scores)
> pairs(t_bin20_all[,269:273], upper.panel=NULL)
> pairs(u_bin21_all[,269:273], upper.panel=NULL)
> plot <- ggplot(u_bin21_all, aes(x=PC1, y=PC2, fill=bin, size=Contig_length)) + geom_point(alpha=0.5, shape=21)
> plot
> u_bin21_all <- subset(u_bin21_all, PC1 < 0.2)
> u_bin21_all <- subset(u_bin21_all, PC2 < 0)
> plot <- ggplot(u_bin21_all, aes(x=PC1, y=PC2, fill=bin, size=Contig_length)) + geom_point(alpha=0.5, shape=21)
> plot
> u_bin21_all <- subset(u_bin21_all, PC1 > -0.3)
> plot <- ggplot(u_bin21_all, aes(x=PC1, y=PC3, fill=bin, size=Contig_length)) + geom_point(alpha=0.5, shape=21)
> plot
> u_bin21_all <- subset(u_bin21_all, PC1 < 0.1)
> u_bin21 <- u_bin21_all[-c(269:273)]
> rda <- rda(u_bin21[,13:268], data=u_bin21[,1:12], scale=T)
> X_scores <- scores(rda, choices = 1:5)$sites
> u_bin21_all <- cbind(u_bin21, X_scores)
> pairs(u_bin21_all[,269:273], upper.panel=NULL)
> plot <- ggplot(u_bin21_all, aes(x=PC1, y=PC2, fill=bin, size=Contig_length)) + geom_point(alpha=0.5, shape=21)
> plot
> plot <- ggplot(u_bin21_all, aes(x=PC4, y=PC2, fill=bin, size=Contig_length)) + geom_point(alpha=0.5, shape=21)
> plot
> u_bin21_all <- subset(u_bin21_all, PC2 < 0)
> u_bin21_all <- subset(u_bin21_all, PC4 > -0.5)
> plot <- ggplot(u_bin21_all, aes(x=PC1, y=PC2, fill=bin, size=Contig_length)) + geom_point(alpha=0.5, shape=21)
> plot
> plot <- ggplot(u_bin21_all, aes(x=GC_content, y=Sequencing_depth, fill=bin, size=Contig_length)) +
geom_point(alpha=0.5, shape=21)
> plot
> plot + ylim(0,100)
Warning message:
Removed 5 rows containing missing values (geom_point).
> plot <- ggplot(u_bin21_all, aes(x=isolation_bias, y=gran_ratio, fill=bin, size=Contig_length)) +
geom_point(alpha=0.5, shape=21)
> plot
> u_bin21_all <- subset(u_bin21_all, gran_ratio < 1)
> u_bin21_all <- subset(u_bin21_all, isolation_bias < 0.35)
> u_bin21 <- u_bin21_all[-c(269:273)]
> rda <- rda(u_bin21[,13:268], data=u_bin21[,1:12], scale=T)
> X_scores <- scores(rda, choices = 1:5)$sites
> u_bin21_all <- cbind(u_bin21, X_scores)
> pairs(u_bin21_all[,269:273], upper.panel=NULL)
> plot <- ggplot(u_bin21_all, aes(x=PC1, y=PC2, fill=bin, size=Contig_length)) + geom_point(alpha=0.5, shape=21)
> plot
> u_bin21_all <- subset(u_bin21_all, PC1 < 0)
> plot <- ggplot(u_bin21_all, aes(x=PC1, y=PC3, fill=bin, size=Contig_length)) + geom_point(alpha=0.5, shape=21)
> plot
> plot <- ggplot(u_bin21_all, aes(x=PC2, y=PC3, fill=bin, size=Contig_length)) + geom_point(alpha=0.5, shape=21)
> plot
> plot <- ggplot(u_bin21_all, aes(x=isolation_bias, y=gran_ratio, fill=bin, size=Contig_length)) +
geom_point(alpha=0.5, shape=21)
> plot
> plot <- ggplot(u_bin21_all, aes(x=GC_content, y=Sequencing_depth, fill=bin, size=Contig_length)) +
geom_point(alpha=0.5, shape=21)
```

```
> plot
> u_bin21_all <- subset(u_bin21_all, Sequencing_depth > 10 | GC_content > 0.59)
> plot <- ggplot(u_bin21_all, aes(x=GC_content, y=Sequencing_depth, fill=bin, size=Contig_length)) +
geom_point(alpha=0.5, shape=21)
> plot
> u_bin21 <- u_bin21_all[-c(269:273)]
> rda <- rda(u_bin21[,13:268], data=u_bin21[,1:12], scale=T)
> X_scores <- scores(rda, choices = 1:5)$sites
> u_bin21_all <- cbind(u_bin21, X_scores)
> pairs(u_bin21_all[,269:273], upper.panel=NULL)
> plot <- ggplot(u_bin21_all, aes(x=PC1, y=PC2, fill=bin, size=Contig_length)) + geom_point(alpha=0.5, shape=21)
> plot
> plot <- ggplot(u_bin21_all, aes(x=PC1, y=GC_content, fill=bin, size=Contig_length)) + geom_point(alpha=0.5,
shape=21)
> plot
> plot <- ggplot(u_bin21_all, aes(x=PC2, y=isolation_bias, fill=bin, size=Contig_length)) + geom_point(alpha=0.5,
shape=21)
> plot
> plot <- ggplot(u_bin21_all, aes(x=gran_ratio, y=isolation_bias, fill=bin, size=Contig_length)) +
geom_point(alpha=0.5, shape=21)
> plot
> u_bin21_all <- subset(u_bin21_all, PC2 < 2)
> plot <- ggplot(u_bin21_all, aes(x=PC2, y=PC3, fill=bin, size=Contig_length)) + geom_point(alpha=0.5, shape=21)
> plot
> u_bin21_all <- subset(u_bin21_all, PC3 > -2)
> View(u_bin21_all)
> View(u_bin21_all)
> temp <- temp[!(temp$Contig %in% u_bin21_all$Contig), ]
> plot <- ggplot(temp, aes(x=gran_ratio, y=Sequencing_depth, fill=bin, size=Contig_length)) + geom_point(alpha=0.5,
shape=21)
> plot + ylim(0,50) + xlim(0,5)
Warning message:
Removed 1058 rows containing missing values (geom_point).
> plot + ylim(0,50) + xlim(0,1)
Warning message:
Removed 36992 rows containing missing values (geom_point).
> plot + ylim(0,20) + xlim(0,1)
Warning message:
Removed 37685 rows containing missing values (geom_point).
> v_bin22 <- subset(temp, gran_ratio > 0.4 & gran_ratio < 1 & Sequencing_depth > 5)
> plot <- ggplot(temp, aes(x=gran_ratio, y=isolation_bias, fill=bin, size=Contig_length)) + geom_point(alpha=0.5,
shape=21)
> plot
> plot <- ggplot(v_bin22, aes(x=gran_ratio, y=isolation_bias, fill=bin, size=Contig_length)) + geom_point(alpha=0.5,
shape=21)
> plot
> v_bin22 <- subset(v_bin22, isolation_bias < 1)
> plot <- ggplot(v_bin22, aes(x=gran_ratio, y=isolation_bias, fill=bin, size=Contig_length)) + geom_point(alpha=0.5,
shape=21)
> plot
> v_bin22 <- subset(v_bin22, isolation_bias < 0.5)
> plot <- ggplot(v_bin22, aes(x=gran_ratio, y=isolation_bias, fill=bin, size=Contig_length)) + geom_point(alpha=0.5,
shape=21)
> plot
```

```
> v_bin22 <- subset(v_bin22, isolation_bias > 0.1)
> v_bin22 <- subset(v_bin22, gran_ratio < 0.9)
> rda <- rda(v_bin22[,13:268], data=v_bin22[,1:12], scale=T)
> X_scores <- scores(rda, choices = 1:5)$sites
> v_bin22_all <- cbind(v_bin22, X_scores)
> pairs(v_bin22_all[,269:273], upper.panel=NULL)
> plot <- ggplot(v_bin22, aes(x=PC1, y=PC2, fill=bin, size=Contig_length)) + geom_point(alpha=0.5, shape=21)
> plot
Error in eval(expr, envir, enclos) : object 'PC1' not found

> plot <- ggplot(v_bin22_all, aes(x=PC1, y=PC2, fill=bin, size=Contig_length)) + geom_point(alpha=0.5, shape=21)
> plot
> v_bin22_all <- subset(v_bin22_all, PC1 > -0.2)
> plot <- ggplot(v_bin22_all, aes(x=PC1, y=PC2, fill=bin, size=Contig_length)) + geom_point(alpha=0.5, shape=21)
> plot
> v_bin22_all <- subset(v_bin22_all, PC1 < 0.5)
> plot <- ggplot(v_bin22_all, aes(x=PC3, y=PC2, fill=bin, size=Contig_length)) + geom_point(alpha=0.5, shape=21)
> plot
> plot <- ggplot(v_bin22_all, aes(x=PC3, y=PC1, fill=bin, size=Contig_length)) + geom_point(alpha=0.5, shape=21)
> plot
> v_bin22_all <- subset(v_bin22_all, PC1 < 0.3)
> v_bin22_all <- subset(v_bin22_all, PC1 > 0)
> v_bin22 <- v_bin22_all[-c(269:273)]
> rda <- rda(v_bin22[,13:268], data=v_bin22[,1:12], scale=T)
> X_scores <- scores(rda, choices = 1:5)$sites
> v_bin22_all <- cbind(v_bin22, X_scores)
> pairs(v_bin22_all[,269:273], upper.panel=NULL)
> plot <- ggplot(v_bin22_all, aes(x=PC1, y=PC2, fill=bin, size=Contig_length)) + geom_point(alpha=0.5, shape=21)
> plot
> v_bin22_all <- subset(v_bin22_all, PC1 > 0)
> plot <- ggplot(v_bin22_all, aes(x=PC1, y=PC2, fill=bin, size=Contig_length)) + geom_point(alpha=0.5, shape=21)
> plot
> v_bin22_all <- subset(v_bin22_all, PC1 > 0.2)
> plot <- ggplot(v_bin22_all, aes(x=PC1, y=PC2, fill=bin, size=Contig_length)) + geom_point(alpha=0.5, shape=21)
> plot
> v_bin22_all <- subset(v_bin22_all, PC1 > 0.3)
> plot <- ggplot(v_bin22_all, aes(x=gran_ratio, y=isolation_bias, fill=bin, size=Contig_length)) +
geom_point(alpha=0.5, shape=21)
> plot
> plot <- ggplot(v_bin22_all, aes(x=GC_content, y=Sequencing_depth, fill=bin, size=Contig_length)) +
geom_point(alpha=0.5, shape=21)
> plot
> v_bin22 <- v_bin22_all[-c(269:273)]
> rda <- rda(v_bin22[,13:268], data=v_bin22[,1:12], scale=T)
> X_scores <- scores(rda, choices = 1:5)$sites
> v_bin22_all <- cbind(v_bin22, X_scores)
> pairs(v_bin22_all[,269:273], upper.panel=NULL)
> plot <- ggplot(v_bin22_all, aes(x=PC1, y=PC2, fill=bin, size=Contig_length)) + geom_point(alpha=0.5, shape=21)
> plot
> v_bin22_all <- subset(v_bin22_all, PC1 > -2)
> v_bin22_all <- subset(v_bin22_all, PC2 < 2)
> v_bin22_all <- subset(v_bin22_all, PC1 < 2)
> v_bin22_all <- subset(v_bin22_all, PC2 > -2)
> v_bin22_all <- subset(v_bin22_all, PC1 > -1 | PC2 < 0)
```

```
> plot <- ggplot(v_bin22, aes(x=gran_ratio, y=isolation_bias, fill=bin, size=Contig_length)) + geom_point(alpha=0.5, shape=21)
> plot
> plot <- ggplot(v_bin22, aes(x=GC_content, y=Sequencing_depth, fill=bin, size=Contig_length)) + geom_point(alpha=0.5, shape=21)
> plot
> temp <- temp[!(temp$Contig %in% v_bin22_all$Contig), ]
> plot <- ggplot(temp, aes(x=gran_ratio, y=Sequencing_depth, fill=bin, size=Contig_length)) + geom_point(alpha=0.5, shape=21)
> plot
> plot + xlim(0,5)
Warning message:
Removed 822 rows containing missing values (geom_point).
> plot + xlim(0,5) + ylim(0,40)
Warning message:
Removed 1182 rows containing missing values (geom_point).
> plot + xlim(0,1) + ylim(0,20)
Warning message:
Removed 37683 rows containing missing values (geom_point).
> w_bin23 <- subset(temp, gran_ratio > 0.1 & gran_ratio < 1 & Sequencing_depth > 5)
> plot <- ggplot(w_bin23, aes(x=gran_ratio, y=isolation_bias, fill=bin, size=Contig_length)) + geom_point(alpha=0.5, shape=21)
> plot
> w_bin23 <- subset(w_bin23, isolation_bias < 1)
> plot <- ggplot(w_bin23, aes(x=gran_ratio, y=isolation_bias, fill=bin, size=Contig_length)) + geom_point(alpha=0.5, shape=21)
> plot
> w_bin23 <- subset(w_bin23, isolation_bias < 0.5)
> plot <- ggplot(w_bin23, aes(x=gran_ratio, y=isolation_bias, fill=bin, size=Contig_length)) + geom_point(alpha=0.5, shape=21)
> plot
> w_bin23 <- subset(w_bin23, isolation_bias > 0.01)
> w_bin23 <- subset(w_bin23, gran_ratio < 0.8)
> w_bin23 <- subset(w_bin23, gran_ratio > 0.2)
> plot <- ggplot(w_bin23, aes(x=gran_ratio, y=isolation_bias, fill=bin, size=Contig_length)) + geom_point(alpha=0.5, shape=21)
> plot
> w_bin23 <- subset(w_bin23, isolation_bias < 0.4)
> w_bin23 <- subset(w_bin23, gran_ratio < 0.7)
> rda <- rda(w_bin23[,13:268], data=w_bin23[,1:12], scale=T)
> X_scores <- scores(rda, choices = 1:5)$sites
> w_bin23_all <- cbind(w_bin23,X_scores)
> pairs(w_bin23_all[,269:273], upper.panel=NULL)
> plot <- ggplot(w_bin23_all, aes(x=PC1, y=PC2, fill=bin, size=Contig_length)) + geom_point(alpha=0.5, shape=21)
> plot
> w_bin23_all <- subset(w_bin23_all, PC1 > 0)
> w_bin23_all <- subset(w_bin23_all, PC2 < 1)
> plot <- ggplot(w_bin23_all, aes(x=PC1, y=PC3, fill=bin, size=Contig_length)) + geom_point(alpha=0.5, shape=21)
> plot
> w_bin23_all <- subset(w_bin23_all, PC3 < 0)
> plot <- ggplot(w_bin23_all, aes(x=PC1, y=PC3, fill=bin, size=Contig_length)) + geom_point(alpha=0.5, shape=21)
> plot
> w_bin23_all <- subset(w_bin23_all, PC3 < 0.2)
> w_bin23_all <- subset(w_bin23_all, PC1 < 0.5)
```

```
> w_bin23_all <- subset(w_bin23_all, PC3 < -0.2)
> plot <- ggplot(w_bin23_all, aes(x=gran_ratio, y=isolation_bias, fill=bin, size=Contig_length)) +
geom_point(alpha=0.5, shape=21)
> plot
> w_bin23_all <- subset(w_bin23_all, gran_ratio > 0.3)
> w_bin23_all <- subset(w_bin23_all, isolation_bias < 0.3)
> plot <- ggplot(w_bin23_all, aes(x=GC_content, y=Sequencing_depth, fill=bin, size=Contig_length)) +
geom_point(alpha=0.5, shape=21)
> plot
> w_bin23 <- w_bin23_all[-c(269:2783)]
> w_bin23 <- w_bin23_all[-c(269:273)]
> rda <- rda(w_bin23[,13:268], data=w_bin23[,1:12], scale=T)
> X_scores <- scores(rda, choices = 1:5)$sites
> w_bin23_all <- cbind(w_bin23,X_scores)
> pairs(w_bin23_all[,269:273], upper.panel=NULL)
> plot <- ggplot(w_bin23_all, aes(x=PC1, y=PC2, fill=bin, size=Contig_length)) + geom_point(alpha=0.5, shape=21)
> plot
> w_bin23_all <- subset(w_bin23_all, PC2 > -1)
> w_bin23_all <- subset(w_bin23_all, PC1 < 0.1)
> plot <- ggplot(w_bin23_all, aes(x=PC1, y=PC2, fill=bin, size=Contig_length)) + geom_point(alpha=0.5, shape=21)
> plot
> w_bin23_all <- subset(w_bin23_all, PC1 > -1.5)
> plot <- ggplot(w_bin23_all, aes(x=GC_content, y=Sequencing_depth, fill=bin, size=Contig_length)) +
geom_point(alpha=0.5, shape=21)
> plot
> plot <- ggplot(w_bin23_all, aes(x=gran_ratio, y=isolation_bias, fill=bin, size=Contig_length)) +
geom_point(alpha=0.5, shape=21)
> plot
> w_bin23 <- w_bin23_all[-c(269:273)]
> rda <- rda(w_bin23[,13:268], data=w_bin23[,1:12], scale=T)
> X_scores <- scores(rda, choices = 1:5)$sites
> w_bin23_all <- cbind(w_bin23,X_scores)
> pairs(w_bin23_all[,269:273], upper.panel=NULL)
> plot <- ggplot(w_bin23_all, aes(x=PC1, y=PC2, fill=bin, size=Contig_length)) + geom_point(alpha=0.5, shape=21)
> plot
> w_bin23_all <- subset(w_bin23_all, PC1 < 1.5)
> w_bin23_all <- subset(w_bin23_all, PC2 < 1.9)
> plot <- ggplot(w_bin23_all, aes(x=GC_content, y=Sequencing_depth, fill=bin, size=Contig_length)) +
geom_point(alpha=0.5, shape=21)
> plot
> temp <- temp[!(temp$Contig %in% w_bin23_all$Contig), ]
> plot <- ggplot(temp, aes(x=GC_content, y=Sequencing_depth, fill=bin, size=Contig_length)) +
geom_point(alpha=0.5, shape=21)
> plot
> plot + ylim(0,50)
Warning message:
Removed 235 rows containing missing values (geom_point).
> plot + ylim(0,20)
Warning message:
Removed 1412 rows containing missing values (geom_point).
> plot <- ggplot(w_bin23_all, aes(x=gran_ratio, y=Sequencing_depth, fill=bin, size=Contig_length)) +
geom_point(alpha=0.5, shape=21)
> plot <- ggplot(temp, aes(x=gran_ratio, y=Sequencing_depth, fill=bin, size=Contig_length)) + geom_point(alpha=0.5,
shape=21)
```

```
> plot
> plot + xlim(0,3)
Warning message:
Removed 2917 rows containing missing values (geom_point).
> plot + xlim(0,3) + ylim(0,30)
Warning message:
Removed 3563 rows containing missing values (geom_point).
> plot + xlim(1,2) + ylim(0,30)
Warning message:
Removed 60332 rows containing missing values (geom_point).
> x_bin24 <- subset(temp, gran_ratio > 1 & gran_ratio < 3)
> plot <- ggplot(x_bin24, aes(x=gran_ratio, y=isolation_bias, fill=bin, size=Contig_length)) + geom_point(alpha=0.5,
shape=21)
> plot
> x_bin24 <- subset(x_bin24, isolation_bias < 1)
> plot <- ggplot(x_bin24, aes(x=gran_ratio, y=isolation_bias, fill=bin, size=Contig_length)) + geom_point(alpha=0.5,
shape=21)
> plot
> x_bin24 <- subset(x_bin24, isolation_bias < 0.5)
> x_bin24 <- subset(x_bin24, isolation_bias > 0.01)
> plot <- ggplot(x_bin24, aes(x=gran_ratio, y=isolation_bias, fill=bin, size=Contig_length)) + geom_point(alpha=0.5,
shape=21)
> plot
> x_bin24 <- subset(x_bin24, isolation_bias < 0.4)
> plot <- ggplot(x_bin24, aes(x=isolation_bias, y=Sequencing_depth, fill=bin, size=Contig_length)) +
geom_point(alpha=0.5, shape=21)
> plot
> plot + ylim(0,20)
Warning message:
Removed 566 rows containing missing values (geom_point).
> x_bin24 <- subset(x_bin24, Sequencing_depth > 4)
> plot <- ggplot(x_bin24, aes(x=gran_ratio, y=isolation_bias, fill=bin, size=Contig_length)) + geom_point(alpha=0.5,
shape=21)
>
> plot
> plot <- ggplot(x_bin24, aes(x=gran_ratio, y=Sequencing_depth, fill=bin, size=Contig_length)) +
geom_point(alpha=0.5, shape=21)
> plot
> plot + ylim(0,20)
Warning message:
Removed 566 rows containing missing values (geom_point).
> x_bin24 <- subset(x_bin24, Sequencing_depth > 6)
> rda <- rda(x_bin24[,13:268], data=x_bin24[,1:12], scale=T)
> X_scores <- scores(rda, choices = 1:5)$sites
> x_bin24
> x_bin24_all <- cbind(x_bin24, X_scores)
> pairs(x_bin24_all[,269:273], upper.panel=NULL)
> plot <- ggplot(x_bin24_all, aes(x=PC1, y=PC2, fill=bin, size=Contig_length)) + geom_point(alpha=0.5, shape=21)
> plot
> plot <- ggplot(x_bin24_all, aes(x=PC1, y=Sequencing_depth, fill=bin, size=Contig_length)) +
geom_point(alpha=0.5, shape=21)
> plot
> plot + ylim(0,40)
Warning message:
```

```
Removed 147 rows containing missing values (geom_point).
> x_bin24_all <- subset(x_bin24_all, PC1 < 0.2)
> plot <- ggplot(x_bin24_all, aes(x=PC1, y=PC2, fill=bin, size=Contig_length)) + geom_point(alpha=0.5, shape=21)
> plot
> x_bin24_all <- subset(x_bin24_all, PC2 > -0.5)
> x_bin24_all <- subset(x_bin24_all, PC1 > -0.5)
> plot <- ggplot(x_bin24_all, aes(x=PC1, y=Sequencing_depth, fill=bin, size=Contig_length)) +
geom_point(alpha=0.5, shape=21)
> plot
> plot + ylim(0,40)
Warning message:
Removed 62 rows containing missing values (geom_point).
> plot + ylim(0,20)
Warning message:
Removed 259 rows containing missing values (geom_point).
> x_bin24_all <- subset(x_bin24_all, Sequencing_depth > 8)
> x_bin24 <- x_bin24_all[-c(269:273)]
> rda <- rda(x_bin24[,13:268], data=x_bin24[,1:12], scale=T)
> X_scores <- scores(rda, choices = 1:5)$sites
> x_bin24_all <- cbind(x_bin24, X_scores)
> pairs(x_bin24_all[,269:273], upper.panel=NULL)
> plot <- ggplot(x_bin24_all, aes(x=PC1, y=PC2, fill=bin, size=Contig_length)) + geom_point(alpha=0.5, shape=21)
> plot
> x_bin24_all <- subset(x_bin24_all, PC2 < -0.4)
> x_bin24_all <- subset(x_bin24_all, PC1 < 0.5)
> plot <- ggplot(x_bin24_all, aes(x=gran_ratio, y=isolation_bias, fill=bin, size=Contig_length)) +
geom_point(alpha=0.5, shape=21)
> plot
> x_bin24_all <- subset(x_bin24_all, isolation_bias < 0.2)
> plot <- ggplot(x_bin24_all, aes(x=GC_content, y=Sequencing_depth, fill=bin, size=Contig_length)) +
geom_point(alpha=0.5, shape=21)
> plot
> x_bin24 <- x_bin24_all[-c(269:273)]
> rda <- rda(x_bin24[,13:268], data=x_bin24[,1:12], scale=T)
> X_scores <- scores(rda, choices = 1:5)$sites
> x_bin24_all <- cbind(x_bin24, X_scores)
> pairs(x_bin24_all[,269:273], upper.panel=NULL)
> plot <- ggplot(x_bin24_all, aes(x=PC1, y=PC2, fill=bin, size=Contig_length)) + geom_point(alpha=0.5, shape=21)
> plot
> plot <- ggplot(x_bin24_all, aes(x=PC3, y=PC2, fill=bin, size=Contig_length)) + geom_point(alpha=0.5, shape=21)
> plot
> x_bin24_all <- subset(x_bin24_all, PC2 < 1)
> x_bin24_all <- subset(x_bin24_all, PC3 > -5)
> plot <- ggplot(x_bin24_all, aes(x=PC3, y=PC2, fill=bin, size=Contig_length)) + geom_point(alpha=0.5, shape=21)
> plot
> x_bin24_all <- subset(x_bin24_all, PC3 < 1.8)
> x_bin24_all <- subset(x_bin24_all, PC2 < 0.7)
> x_bin24_all <- subset(x_bin24_all, PC3 > -1.8)
> plot <- ggplot(x_bin24_all, aes(x=PC3, y=PC2, fill=bin, size=Contig_length)) + geom_point(alpha=0.5, shape=21)
> plot
> plot <- ggplot(x_bin24_all, aes(x=GC_content, y=Sequencing_depth, fill=bin, size=Contig_length)) +
geom_point(alpha=0.5, shape=21)
> plot
> plot <- ggplot(x_bin24_all, aes(x=gran_ratio, y=isolation_bias, fill=bin, size=Contig_length)) +
```

```
geom_point(alpha=0.5, shape=21)
> plot
> plot <- ggplot(x_bin24_all, aes(x=GC_content, y=isolation_bias, fill=bin, size=Contig_length)) +
geom_point(alpha=0.5, shape=21)
> plot
> x_bin24_all <- subset(x_bin24_all, gran_ratio < 2.3)
> plot <- ggplot(x_bin24_all, aes(x=GC_content, y=isolation_bias, fill=bin, size=Contig_length)) +
geom_point(alpha=0.5, shape=21)
> plot
> temp <- temp[!(temp$Contig %in% x_bin24_all$Contig), ]
> y_bin25 <- subset(temp, gran_ratio > 1 & gran_ratio < 3)
> y_bin25 <- subset(y_bin25, Sequencing_depth > 4)
> y_bin25 <- subset(y_bin25, Sequencing_depth > 6)
> rda <- rda(y_bin25[,13:268], data=y_bin25[,1:12], scale=T)
> X_scores <- scores(rda, choices = 1:5)$sites
> y_bin25_all <- cbind(y_bin25, X_scores)
> pairs(y_bin25_all[,269:273], upper.panel=NULL)
> plot <- ggplot(y_bin25_all, aes(x=PC1, y=PC2, fill=bin, size=Contig_length)) + geom_point(alpha=0.5, shape=21)
> plot
> plot <- ggplot(y_bin25_all, aes(x=PC1, y=Sequencing_depth, fill=bin, size=Contig_length)) +
geom_point(alpha=0.5, shape=21)
> plot
> plot + ylim(0,20)
Warning message:
Removed 598 rows containing missing values (geom_point).
> y_bin25_all <- subset(y_bin25_all, PC1 > 0.4)
> plot <- ggplot(y_bin25_all, aes(x=gran_ratio, y=isolation_bias, fill=bin, size=Contig_length)) +
geom_point(alpha=0.5, shape=21)
> plot
> y_bin25_all <- subset(y_bin25_all, isolation_bias < 0.5)
> plot <- ggplot(y_bin25_all, aes(x=gran_ratio, y=isolation_bias, fill=bin, size=Contig_length)) +
geom_point(alpha=0.5, shape=21)
> plot
> y_bin25_all <- subset(y_bin25_all, isolation_bias < 0.3)
> y_bin25_all <- subset(y_bin25_all, gran_ratio < 2.5)
> y_bin25 <- y_bin25_all[-c(269:273)]
> rda <- rda(y_bin25[,13:268], data=y_bin25[,1:12], scale=T)
> X_scores <- scores(rda, choices = 1:5)$sites
> y_bin25_all <- cbind(y_bin25, X_scores)
> pairs(y_bin25_all[,269:273], upper.panel=NULL)
> plot <- ggplot(y_bin25_all, aes(x=PC1, y=PC2, fill=bin, size=Contig_length)) + geom_point(alpha=0.5, shape=21)
> plot
> y_bin25_all <- subset(y_bin25_all, PC2 > -0.5)
> y_bin25_all <- subset(y_bin25_all, PC1 < 0.5)
> plot <- ggplot(y_bin25_all, aes(x=gran_ratio, y=isolation_bias, fill=bin, size=Contig_length)) +
geom_point(alpha=0.5, shape=21)
> plot
> plot <- ggplot(y_bin25_all, aes(x=GC_content, y=Sequencing_depth, fill=bin, size=Contig_length)) +
geom_point(alpha=0.5, shape=21)
> plot
> plot +ylim(0,20)
Warning message:
Removed 49 rows containing missing values (geom_point).
> y_bin25 <- y_bin25_all[-c(269:273)]
```

```
> rda <- rda(y_bin25[,13:268], data=y_bin25[,1:12], scale=T)
> X_scores <- scores(rda, choices = 1:5)$sites
> y_bin25_all <- cbind(y_bin25, X_scores)
> pairs(y_bin25_all[,269:273], upper.panel=NULL)
> plot <- ggplot(y_bin25_all, aes(x=PC1, y=PC2, fill=bin, size=Contig_length)) + geom_point(alpha=0.5, shape=21)
> plot
> plot <- ggplot(y_bin25_all, aes(x=PC3, y=PC2, fill=bin, size=Contig_length)) + geom_point(alpha=0.5, shape=21)
> plot
> y_bin25_all <- subset(y_bin25_all, PC3 > -0.5)
> y_bin25_all <- subset(y_bin25_all, PC2 > -0.5)
> plot <- ggplot(y_bin25_all, aes(x=PC3, y=PC2, fill=bin, size=Contig_length)) + geom_point(alpha=0.5, shape=21)
> plot
> plot <- ggplot(y_bin25_all, aes(x=PC2, y=Sequencing_depth, fill=bin, size=Contig_length)) +
geom_point(alpha=0.5, shape=21)
> plot
> plot + ylim(0,20)
Warning message:
Removed 30 rows containing missing values (geom_point).
> plot <- ggplot(y_bin25_all, aes(x=PC3, y=Sequencing_depth, fill=bin, size=Contig_length)) +
geom_point(alpha=0.5, shape=21)
> plot + ylim(0,20)
Warning message:
Removed 30 rows containing missing values (geom_point).
> y_bin25_all <- subset(y_bin25_all, PC3 > 0 | Sequencing_depth > 10)
> plot <- ggplot(y_bin25_all, aes(x=PC3, y=PC2, fill=bin, size=Contig_length)) + geom_point(alpha=0.5, shape=21)
> plot
> plot <- ggplot(y_bin25_all, aes(x=GC_content, y=Sequencing_depth, fill=bin, size=Contig_length)) +
geom_point(alpha=0.5, shape=21)
> plot
> plot <- ggplot(y_bin25_all, aes(x=PC1, y=PC2, fill=bin, size=Contig_length)) + geom_point(alpha=0.5, shape=21)
> plot
> y_bin25_all <- subset(y_bin25_all, PC2 > 0)
> plot <- ggplot(y_bin25_all, aes(x=PC1, y=PC2, fill=bin, size=Contig_length)) + geom_point(alpha=0.5, shape=21)
> plot
> plot <- ggplot(y_bin25_all, aes(x=GC_content, y=Sequencing_depth, fill=bin, size=Contig_length)) +
geom_point(alpha=0.5, shape=21)
> plot
> y_bin25 <- y_bin25_all[-c(269:273)]
> rda <- rda(y_bin25[,13:268], data=y_bin25[,1:12], scale=T)
> X_scores <- scores(rda, choices = 1:5)$sites
> y_bin25_all <- cbind(y_bin25, X_scores)
> pairs(y_bin25_all[,269:273], upper.panel=NULL)
> plot <- ggplot(y_bin25_all, aes(x=PC1, y=PC2, fill=bin, size=Contig_length)) + geom_point(alpha=0.5, shape=21)
> plot
> plot <- ggplot(y_bin25_all, aes(x=PC2, y=isolation_bias, fill=bin, size=Contig_length)) + geom_point(alpha=0.5,
shape=21)
> plot
> plot <- ggplot(y_bin25_all, aes(x=PC1, y=isolation_bias, fill=bin, size=Contig_length)) + geom_point(alpha=0.5,
shape=21)
> plot
> y_bin25_all <- subset(y_bin25_all, PC1 < 2)
> plot <- ggplot(y_bin25_all, aes(x=PC2, y=gran_ratio, fill=bin, size=Contig_length)) + geom_point(alpha=0.5,
shape=21)
> plot
```

```
> y_bin25_all <- subset(y_bin25_all, PC2 < 1.5)
> plot <- ggplot(y_bin25_all, aes(x=GC_content, y=Sequencing_depth, fill=bin, size=Contig_length)) +
geom_point(alpha=0.5, shape=21)
> plot
> plot <- ggplot(y_bin25_all, aes(x=PC1, y=PC2, fill=bin, size=Contig_length)) + geom_point(alpha=0.5, shape=21)
> plot
> plot <- ggplot(y_bin25_all, aes(x=isolation_bias, y=gran_ratio, fill=bin, size=Contig_length)) +
geom_point(alpha=0.5, shape=21)
> plot
> y_bin25_all <- subset(y_bin25_all, isolation_bias < 0.25)
> plot <- ggplot(y_bin25_all, aes(x=GC_content, y=Sequencing_depth, fill=bin, size=Contig_length)) +
geom_point(alpha=0.5, shape=21)
> plot
> plot <- ggplot(y_bin25_all, aes(x=isolation_bias, y=gran_ratio, fill=bin, size=Contig_length)) +
geom_point(alpha=0.5, shape=21)
> plot
> temp <- temp[!(temp$Contig %in% y_bin25_all$Contig), ]
> plot <- ggplot(temp, aes(x=isolation_bias, y=Sequencing_depth, fill=bin, size=Contig_length)) +
geom_point(alpha=0.5, shape=21)
> plot
> plot + xlim(0,2) + ylim(0,50)
Warning message:
Removed 388 rows containing missing values (geom_point).
> plot + xlim(0,2) + ylim(0,20)
Warning message:
Removed 1554 rows containing missing values (geom_point).
> plot + xlim(0.5,2) + ylim(0,20)
Warning message:
Removed 79565 rows containing missing values (geom_point).
> plot <- ggplot(temp, aes(x=gran_ratio, y=Sequencing_depth, fill=bin, size=Contig_length)) + geom_point(alpha=0.5,
shape=21)
> plot
> plot + xlim(0,5)
Warning message:
Removed 822 rows containing missing values (geom_point).
> plot + xlim(0,2) + ylim(0),40)
Error: unexpected ',' in "plot + xlim(0,2) + ylim(0),"
> plot + xlim(0,2) + ylim(0,40)
Warning message:
Removed 8971 rows containing missing values (geom_point).
> plot + xlim(0,3) + ylim(0,40)
Warning message:
Removed 3260 rows containing missing values (geom_point).
> plot + xlim(0,1) + ylim(0,20)
Warning message:
Removed 36900 rows containing missing values (geom_point).
> X_rest <- subset(temp, Sequencing_depth > 10)
> plot <- ggplot(X_rest, aes(x=isolation_bias, y=gran_ratio, fill=bin, size=Contig_length)) + geom_point(alpha=0.5,
shape=21)
> plot
> plot <- ggplot(X_rest, aes(x=Average_coverage_ctab, y=Average_coverage_kit, fill=bin, size=Contig_length)) +
geom_point(alpha=0.5, shape=21)
> plot
> plot <- ggplot(X_rest, aes(x=Average_coverage_gran, y=Average_coverage_total, fill=bin, size=Contig_length)) +
```

```
geom_point(alpha=0.5, shape=21)
> plot
> plot + xlim(0,100) + ylim(0,100)
Warning message:
Removed 28 rows containing missing values (geom_point).
> plot
> plot + xlim(0,50) + ylim(0,50)
Warning message:
Removed 82 rows containing missing values (geom_point).
> rda <- rda(X_rest[,13:268], data=X_rest[,1:12], scale=T)
> X_scores <- scores(rda, choices = 1:5)$sites
> X_rest_all <- cbind(X_rest,X_scores)
> pairs(X_rest_all[269:273], upper.panel=NULL)
> plot <- ggplot(X_rest_all, aes(x=PC1, y=PC2, fill=bin, size=Contig_length)) + geom_point(alpha=0.5, shape=21)
> plot
```
